# Reconfiguration of Structural and Functional Connectivity Coupling in Patient Subgroups With Adolescent Depression

**DOI:** 10.1001/jamanetworkopen.2024.1933

**Published:** 2024-03-12

**Authors:** Ming Xu, Xuemei Li, Teng Teng, Yang Huang, Mengqi Liu, Yicheng Long, Fajin Lv, Dongmei Zhi, Xiang Li, Aichen Feng, Shan Yu, Vince Calhoun, Xinyu Zhou, Jing Sui

**Affiliations:** 1 Brainnetome Center and National Laboratory of Pattern Recognition, Institute of Automation, Chinese Academy of Sciences, Beijing, China; 2 School of Artificial Intelligence, University of Chinese Academy of Sciences, Beijing, China; 3 Department of Psychiatry, The First Affiliated Hospital of Chongqing Medical University, Chongqing, China; 4 Department of Radiology, The First Affiliated Hospital of Chongqing Medical University, Chongqing, China; 5 Department of Psychiatry and National Clinical Research Center for Mental Disorders, The Second Xiangya Hospital of Central South University, Hunan, China; 6 International Data Group (IDG)/McGovern Institute for Brain Research, State Key Laboratory of Cognitive Neuroscience and Learning, Beijing Normal University, Beijing, China; 7 Tri-institutional Center for Translational Research in Neuroimaging and Data Science (TReNDS), Georgia Institute of Technology, Emory University and Georgia State University, Atlanta, Georgia

## Abstract

**Importance:**

Adolescent major depressive disorder (MDD) is associated with serious adverse implications for brain development and higher rates of self-injury and suicide, raising concerns about its neurobiological mechanisms in clinical neuroscience. However, most previous studies regarding the brain alterations in adolescent MDD focused on single-modal images or analyzed images of different modalities separately, ignoring the potential role of aberrant interactions between brain structure and function in the psychopathology.

**Objective:**

To examine alterations of structural and functional connectivity (SC-FC) coupling in adolescent MDD by integrating both diffusion magnetic resonance imaging (MRI) and resting-state functional MRI data.

**Design, Setting, and Participants:**

This cross-sectional study recruited participants aged 10 to 18 years from January 2, 2020, to December 28, 2021. Patients with first-episode MDD were recruited from the outpatient psychiatry clinics at The First Affiliated Hospital of Chongqing Medical University. Healthy controls were recruited by local media advertisement from the general population in Chongqing, China. The sample was divided into 5 subgroup pairs according to different environmental stressors and clinical characteristics. Data were analyzed from January 10, 2022, to February 20, 2023.

**Main Outcomes and Measures:**

The SC-FC coupling was calculated for each brain region of each participant using whole-brain SC and FC. Primary analyses included the group differences in SC-FC coupling and clinical symptom associations between SC-FC coupling and participants with adolescent MDD and healthy controls. Secondary analyses included differences among 5 types of MDD subgroups: with or without suicide attempt, with or without nonsuicidal self-injury behavior, with or without major life events, with or without childhood trauma, and with or without school bullying.

**Results:**

Final analyses examined SC-FC coupling of 168 participants with adolescent MDD (mean [mean absolute deviation (MAD)] age, 16.0 [1.7] years; 124 females [73.8%]) and 101 healthy controls (mean [MAD] age, 15.1 [2.4] years; 61 females [60.4%]). Adolescent MDD showed increased SC-FC coupling in the visual network, default mode network, and insula (Cohen *d* ranged from 0.365 to 0.581; false discovery rate [FDR]–corrected *P* < .05). Some subgroup-specific alterations were identified via subgroup analyses, particularly involving parahippocampal coupling decrease in participants with suicide attempt (partial η^2^ = 0.069; 90% CI, 0.025-0.121; FDR-corrected *P* = .007) and frontal-limbic coupling increase in participants with major life events (partial η^2^ ranged from 0.046 to 0.068; FDR-corrected *P* < .05).

**Conclusions and Relevance:**

Results of this cross-sectional study suggest increased SC-FC coupling in adolescent MDD, especially involving hub regions of the default mode network, visual network, and insula. The findings enrich knowledge of the aberrant brain SC-FC coupling in the psychopathology of adolescent MDD, underscoring the vulnerability of frontal-limbic SC-FC coupling to external stressors and the parahippocampal coupling in shaping future-minded behavior.

## Key Points

**Question:**

How does the relationship between brain structure and function change in patients with adolescent major depressive disorder (MDD) and its subgroups?

**Findings:**

In this cross-sectional study of 168 participants with adolescent MDD and 101 healthy controls, common and unique structural and functional connectivity (SC-FC) coupling changes were identified in hub regions of the default mode network, visual network, and frontal-limbic circuit.

**Meaning:**

Findings of this study enrich knowledge of the aberrant SC-FC coupling in the psychopathology of adolescent MDD and underscore the SC-FC coupling vulnerability to external stressors and interactions with adverse behavior.

## Teaser

This cross-sectional study uses neuroimaging to assess changes in structural and functional connectivity coupling in adolescent patients with major depressive disorder.

## Introduction

Major depressive disorder (MDD) onset occurs more frequently during adolescence than any other age group due to various factors, such as environmental stressors and hormonal changes,^1,2^ and MDD has been a leading cause of disability worldwide.^3,4^ Compared with adult-onset MDD, adolescent MDD has more destructive developmental implications, including serious social functioning impairments, poor school achievements, increased risk of self-injurious behavior, and suicide attempts.^5,6^ Additionally, most children and adolescents with MDD have mental disorder comorbidities and high symptomatic heterogeneity,^7,8^ highlighting the need to better understand the neurobiological basis of adolescent MDD and its subgroups for appropriate treatment options.

Previous findings have suggested that the neurobiological basis of depressive symptom profiles in adolescents is associated with complex interactions between environment and multimodal brain development. For example, functional magnetic resonance imaging (fMRI) studies have revealed altered activities of neural circuits implicated in emotion regulation, cognitive control, and reward processing in adolescent MDD, particularly involving dysregulation of the limbic system, default mode network (DMN), and frontoparietal network.^9,10,11,12,13^ Moreover, brain morphological and microstructural changes have been observed in adolescent MDD, such as surface area reductions via structural MRI,^14^ hippocampal and frontal white matter volume reductions,^12,15^ and altered microstructure in uncinate fasciculi and corpus callosum via diffusion MRI.^16,17^ More recently, considering the implications of diverse environmental exposures for brain development, interest has increased in the neural substrates associated with environmental risk factors and typical clinical profiles in adolescent MDD.^18,19^

However, beyond the functional or structural impairment in cortex or connectivity, it remains underexplored whether the structural connectivity (SC) and functional connectivity (FC) coupling is disrupted in adolescent MDD and how such disruption is associated with clinical characteristics and environmental stressors among MDD subgroups. Convergent evidence has revealed that brain structure and function are inherently intertwined and coupled. Expanding from animal model studies,^20,21^ MRI studies in humans have demonstrated correspondence between FC and SC at various spatiotemporal scales, suggesting the anatomical basis of brain functional organizations.^22^ Emerging studies have also indicated aberrant brain structure-function interactions in the pathology of psychiatric disorders.^23,24,25^ To this end, the primary goal of the present study was to examine the alterations of SC-FC coupling in adolescent MDD by integrating both diffusion MRI and resting-state fMRI data. This study aimed to provide a novel insight into the neurobiological basis of adolescent MDD with different clinical characteristics by integrating multimodal brain images.

## Methods

The Chongqing Medical University Ethics Committee approved this cross-sectional study. All participants and their parent or guardian received information on study procedures and signed an informed consent form to participate. We followed the Strengthening the Reporting of Observational Studies in Epidemiology (STROBE) reporting guideline.

The study design is shown in eFigure 1 in Supplement 1. Intuitively, the SC-FC coupling describes the extent to which FC can be inferred from the corresponding SC, and a higher SC-FC coupling indicates anatomical constraints for functional communications (eFigure 1A in Supplement 1). We compared MDD as a whole (eFigure 1B in Supplement 1) and its subgroup variations on SC-FC coupling (eFigure 1C in Supplement 1) by partitioning participants into 5 subgroup pairs according to different clinical characteristics and environmental stressors: with or without suicide attempt, with or without nonsuicidal self-injury (NSSI) behavior, with or without major life event (MLE), with or without childhood trauma, and with or without school bullying. We evaluated the association between SC-FC coupling and depressive and anxiety symptoms by correlation analyses (eFigure 1D in Supplement 1).

### Participants

Patients aged 10 to 18 years with MDD and who were first-episode drug naive and receiving first-episode antipsychotic medication were recruited from outpatient psychiatry clinics at The First Affiliated Hospital of Chongqing Medical University in Chongqing, China. Healthy controls aged 10 to 18 years were recruited from the general population through local media advertisement. All participants were recruited from January 2, 2020, to December 28, 2021.

Adolescent MDD was diagnosed with the *Diagnostic and Statistical Manual of Mental Disorders* (Fifth Edition) (*DSM-5*). Depression symptom and anxiety symptom severities were assessed using the 17-item Hamilton Depression Rating Scale (HAMD-17; score range: 0-34, with the highest score indicating the most severe depression symptom) and 14-item Hamilton Anxiety Rating Scale (HAMA; score range: 0-36, with the highest score indicating the most severe anxiety symptom), respectively. Childhood trauma experiences were assessed using the self-reported Childhood Trauma Questionnaire (eMethods in Supplement 1). Suicide attempts and NSSI behavior as well as MLEs and school bullying were assessed through clinical interviews (eMethods in Supplement 1).

Inclusion criteria for participants with MDD were (1) MDD diagnosis according to *DSM-5* criteria and HAMD-17 total score higher than 7 points, (2) first-episode depression, and (3) right-handedness. Healthy controls were required to have a HAMD-17 total score of 7 points or lower. Exclusion criteria for all participants were (1) the presence or history of severe medical, neurological, or psychiatric disorders; (2) substance use disorder, head trauma, or loss of consciousness; and (3) any condition that was not suitable for MRI scanning.

### Image Acquisition and Preprocessing

T1-weighted images, resting-state fMRI, and diffusion tensor imaging (DTI) were acquired using a 3T scanner (Magnetom Skyra; Siemens); image acquisition parameters are provided in the eMethods in Supplement 1. All available fMRI and DTI were preprocessed using fMRIPrep 20.2.5^26^ and QSIPrep 0.14.3,^27^ respectively. Preprocess of T1-weighted images included bias correction, skull-stripping, and normalizing to MNI (Montreal Neurological Institute) standard space. Preprocess of resting-state fMRI included motion correction, slice-timing correction, susceptibility distortion correction, and registration. For DTI data, the preprocess included denoising, B1-field correction, eddy current correction, DTI–T1-weighted alignment, model fitting, and region-to-region probabilistic tractography based on the Brainnetome Atlas (region definition in eTable 1 in Supplement 1),^28^ finally yielding a 246-by-246 streamline count matrix. Image preprocessing, tractography, and quality control details are provided in the eMethods and eFigure 2 in Supplement 1.

### Network Construction

The fMRI frames that exceeded a threshold of 0.5-mm framewise displacement or 1.5 standardized DVARS (spatial SD of successive difference images) were scrubbed. The scrubbed preprocessed BOLD (blood oxygenation level dependent) image was then linearly detrended, band-pass filtered (0.01-0.08 Hz), confounder regressed (total of 36 confound regressors),^29^ and standardized using the nilearn.image.clean_image function. After preprocessing and denoising, functional connectivity between each pair of brain regions was calculated as the Fisher *z*-transformed Pearson correlation coefficient between the mean regional residual BOLD time series, resulting in a 246-by-246 weighted adjacent matrix for each participant.

Streamline counts were normalized by the mean volumes of the seed and target region, resulting in a normalized connection weight matrix. Consistency-based thresholding (coefficient of variation below the 25th percentile) was used to mitigate potentially spurious and false-positive anatomical connections.^30^ The structural connectivity matrix was symmetrized by calculating the mean of the upper triangular part and lower triangular part (they were highly correlated) to get an undirected structural connectivity.

The sparse structural connectivity matrix was transformed into fully weighted matrices based on a suite of communication models (eMethods in Supplement 1) that incorporated both centralized and decentralized processes, topological similarities, and spatial embeddings.^31^ A total of 34 adjacent matrices were included.

### Regional SC-FC Coupling

Principal component analysis was performed on all flattened structure–related networks (SC plus 34 SC-based communication models plus the euclidean distance between each region pair) to yield orthogonal anatomical predictors for each participant. The top-n (first n with the greatest variance) principal components that collectively accounted for more than 80% of variance across participants were used in a multilinear regression model for predicting regional FC profiles (eFigure 1A in Supplement 1). The dependent variable of the multilinear regression model was the FC profile of 1 region (1 column or row of the FC). The independent variables were the corresponding regional structural profiles extracted from the principal components. All dependent variables were *z*-scored. The Pearson correlation coefficient *r_i_* that quantifies the correspondence between structural and functional profiles of brain region *i* was defined as the SC-FC coupling at region *i*.

### Statistical Analysis

All statistical analyses were performed from January 10, 2022, to February 20, 2023, using R, version 4.2.2 (R Project for Statistical Computing) and MATLAB R2018b (MathWorks). Multiple comparisons were controlled using the false discovery rate (FDR; Benjamini-Hochberg method), and the FDR-corrected *P* values were explicitly stated. For all models, diagnostic statistics and graphic outputs were examined to assess model assumptions.

For sociodemographic and clinical data, 2-sided Wilcoxon rank sum test (*z*) and Kruskal-Wallis test (*H*) were used to analyze differences of continuous variables between 2 or more groups. The χ^2^ test was performed to analyze interactions between groups and categorical variables when each count was 5 or higher, such as sex, environmental risk exposure, and behavioral characteristics; otherwise, Fisher exact test was performed.

To examine the differences in regional SC-FC coupling between participants with adolescent MDD and healthy controls, a linear model was established with SC-FC coupling of each brain region as the dependent variable and with group indicator as the independent variable. To minimize confounding (eg, potential procedural factors affecting image quality or nonlinear or sex-differed brain development during youth), we included age, sex, age^2^, age by sex, age^2^ by sex, body mass index (BMI; calculated as weight in kilograms divided by height in meters squared), total intracranial volume (ICV), treatment history, and in-scanner head motion (root-mean-squared framewise displacement) as covariates.^32,33,34^ Cohen *d* was calculated as the measure of effect size for group differences in each brain region, and 2-sided *P* < .05 indicated statistical significance. To further control for sample heterogeneity due to demographic variables and assess the robustness of results, we repeated the group comparison analysis in an age-, sex-, BMI-matched subset of participants; this subset was generated by propensity score matching methods (eMethods in Supplement 1).

Subgroup analyses were conducted to compare SC-FC coupling among 5 types of MDD subgroups and healthy controls by the linear model, including age, sex, age^2^, age by sex, age^2^ by sex, BMI, total ICV, treatment history, and in-scanner head motion as covariates. We primarily analyzed the MDD subgroups with or without suicide attempt, NSSI behavior, MLE, childhood trauma, and school bullying. Partial η^2^ and 90% CI were calculated as the measure of subgroup variations in each brain region, and 1-sided *P* < .05 indicated statistical significance. Post hoc 2-sample, 2-tailed *t* tests and Cohen *d* were calculated according to the estimated marginal means of each brain region showing significant subgroup variations to identify the pairwise SC-FC coupling differences at 95% CI (eTables 9-19 in Supplement 1).

Considering the non-gaussian distribution of clinical metrics, we calculated the partial Spearman correlation coefficient (partial *r*), with adjustment for age, sex, age^2^, age by sex, age^2^ by sex, BMI, total ICV, treatment history, and in-scanner head motion (eFigure 1D in Supplement 1). Furthermore, enrichment analysis via spin-based permutation testing was used to evaluate the distribution of the associations across the cerebral cortex (eMethods in Supplement 1).

## Results

After exclusion and image-quality control, we included 168 participants with adolescent MDD (mean [mean absolute deviation (MAD)] age, 16.0 [1.7] years; 124 females [73.8%], 44 males [26.2%]) and 101 healthy controls (mean [MAD] age, 15.1 [2.4] years; 61 females [60.4%], 40 males [39.6%]). Sociodemographic and clinical characteristics of all participants are summarized in the Table and eTables 2 to 6 in Supplement 1.

**Table.** Sociodemographic and Clinical Characteristics of the Sample

Characteristic	Healthy controls (n = 101), No. (%)	Participants with adolescent MDD (n = 168), No. (%)	Statistics	*P* value
Demographic variables				
Age, median (MAD), y	15.1 (2.4)	16.0 (1.7)	*z* = 3.242	.002
Sex				
Male	40 (39.6)	44 (26.2)	χ^2^_1_ = 5.285	.02
Female	61 (60.4)	124 (73.8)		
BMI, median (MAD)	21.0 (3.9)	20.9 (4.1)	*z* = −0.567	.57
Environmental variables				
Divorced parents				
With	5 (5.0)	31 (18.5)	OR (95% CI) = 4.34 (1.59-14.76)	.001
Without	96 (95.0)	137 (81.5)		
Childhood trauma^^a^^				
With	10 (9.9)	106 (63.1)	χ^2^_1_ = 72.772	<.001
Without	91 (90.1)	62 (36.9)		
School bullying^^a^^				
With	8 (7.9)	70 (41.7)	χ^2^_1_ = 34.889	<.001
Without	93 (92.1)	98 (58.3)		
MLE^^a^^				
With	14 (13.9)	61 (36.3)	χ^2^_1_ = 15.808	<.001
Without	87 (76.1)	107 (63.7)		
CTQ score, median (MAD)	34.0 (4.0)	42.0 (6.0)	*z* = 7.110	<.001
Behavioral variables				
Suicide attempt^^a^^				
With	0	55 (32.7)	OR (95% CI) = ∞ (12.37-∞); χ^2^_1_ = 41.564	<.001
Without	101 (100.0)	113 (67.3)		
NSSI behavior^^a^^				
With	2 (2.0)	103 (61.3)	OR (95% CI) = 78.43 (19.65-669.38); χ^2^_1_ = 93.302	<.001
Without	99 (98.0)	65 (38.7)		
Smoking				
Yes	3 (3.0)	17 (10.1)	OR (95% CI) = 3.68 (1.02-20.02)	.03
No	98 (97.0)	151 (89.9)		
Drinking				
Yes	10 (9.9)	20 (11.9)	χ21 = 0.256	.61
No	91 (90.1)	148 (88.1)		
Clinical variables				
HAMA score, median (MAD)	1.0 (1.0)	14.0 (5.0)	*z* = 13.482	<.001
HAMD-17 score, median (MAD)	1.0 (1.0)	18.0 (4.0)	*z* = 13.665	<.001

Abbreviations: BMI, body mass index (calculated as weight in kilograms divided by height in meters squared); CTQ, Childhood Trauma Questionnaire; HAMA, 14-item Hamilton Anxiety Rating Scale; HAMD-17, 17-item Hamilton Depression Rating Scale; MAD, mean absolute deviation; MLE, major life event; MDD, major depressive disorder; NSSI, nonsuicidal self-injury; OR, odds ratio.

^a^ Factors used for dividing patient subgroups.

As expected, participants with adolescent MDD experienced higher rates of behavioral problems and adversity exposure compared with healthy controls. Specifically, 114 participants (67.9%) had a history of suicide attempt (55 [32.7%]; χ^2^_1_ = 41.564; *P* < .001) or NSSI behavior (103 [61.3%]; χ^2^_1_ = 93.302; *P* < .001), and 44 (26.2%) had both. Additionally, 156 participants (92.9%) with adolescent MDD had exposure to childhood trauma (106 [63.1%]; χ^2^_1_ = 72.772; *P* < .001), school bullying (70 [41.7%]; χ^2^_1_ = 34.889; *P* < .001), or MLEs (61 [36.3%]; χ^2^_1_ = 15.808; *P* < .001). Patient subgroups with different clinical characteristics did not differ in history of medication, whereas significant age and sex differences were observed among healthy controls and MDD subgroups (eTables 2-6 in Supplement 1). For example, both age (*H*_2266_ = 11.021; *P* = .004) and sex (χ^2^_2_ = 10.448; *P* = .005) differed among healthy controls and participants with and without suicide attempt (eTable 5 in Supplement 1).

### Aberrant SC-FC Coupling Between Participants With Adolescent MDD and Healthy Controls

The top 4 structural principal components were used for calculating SC-FC coupling, which could collectively account for more than 80% of the variance of 36 structural-related networks in each participant (eFigure 3 in Supplement 1). The composition and explanation for the 4 principal components are shown in eFigures 4 to 7 in Supplement 1.

**Figure 1.**
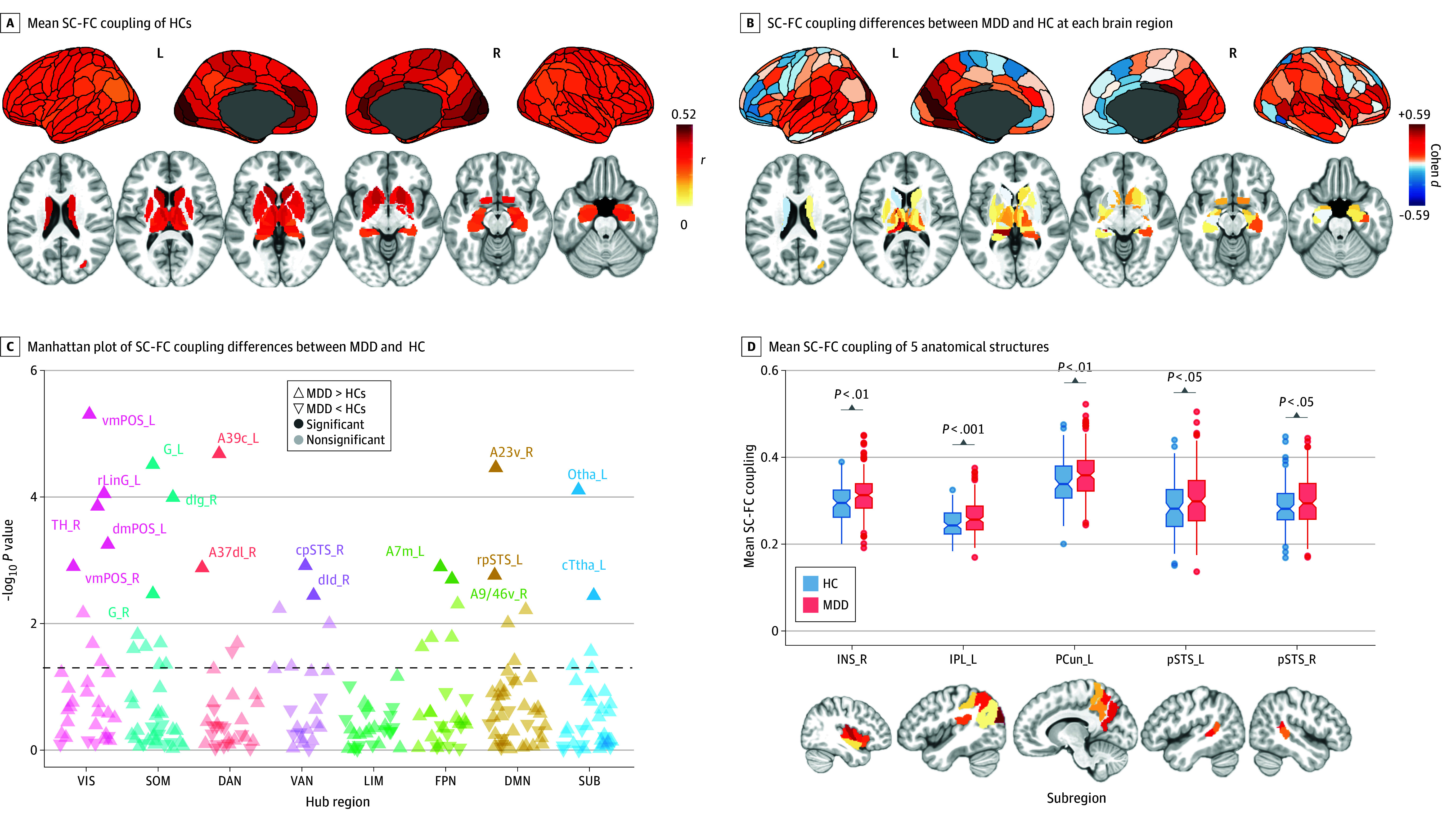
Structural and Functional Connectivity (SC-FC) Coupling Differences Between Participants With Adolescent Major Depressive Disorder (MDD) and Healthy Controls (HCs) A, Primary sensory cortex, cingulate cortex, and basal ganglia exhibited relatively high SC-FC coupling. Inferior parietal lobe (IPL), lateral temporal areas, precuneus (PCun), and hippocampus exhibited relatively low SC-FC coupling. B, Compared with HCs, participants with adolescent MDD presented significantly increased SC-FC coupling in subregions of postcingulate cortex and ventral occipital lobule. C, Regions with within-group differences in SC-FC coupling (false discovery rate [FDR]–corrected *P* < .05; 246 tests) are labeled. Dashed horizontal line indicates −log_10_ uncorrected *P* = .05. D, Mean SC-FC coupling significantly increased in participants with adolescent MDD (FDR-corrected *P* < .05; 48 tests). The upper and lower bounds of the boxes represents the first and third quartile, respectively; horizontal lines, median values; whiskers, 1.5 × of upper and lower bounds of IQRs; and circles above and below boxes, outliers. DAN, dorsal attention network; DMN, default mode network; FPN, frontoparietal network; INS, insula; L, left; LIM, limbic network; pSTS, posterior superior temporal sulcus; R, right; SOM, somatosensory network; SUB, subcortical network; VAN, ventral attention network; VIS, visual network. eTable 1 in Supplement 1 provides details on brain subregions.

Considerable correspondence between the SC-predicted regional FC and the actual regional FC was observed in heathy brain (mean [SD] SC-FC coupling, 0.337 [0.056]). As expected, the magnitude of this correspondence varied widely across the brain (Figure 1A). The maximum mean (SD) SC-FC coupling located at right caudal cuneus gyrus was 0.518 (0.100), and the minimum mean (SD) SC-FC coupling located at left rostroventral area of inferior parietal lobe was 0.207 (0.056). Contributions of each principal component to predicting the regional FCs in healthy brain were also analyzed (eFigure 8 in Supplement 1).

Compared with healthy controls, participants with adolescent MDD exhibited significantly increased SC-FC coupling at 18 subregions (Figure 1B and C; eTable 7 in Supplement 1). These subregions were primarily involved in visual network (VIS) and DMN (defined by Yeo et al^35^ plus subcortical areas; eTable 8 in Supplement 1), including bilateral ventromedial parieto-occipital sulcus (left: Cohen *d* = 0.581 [95% CI, 0.332-0.830, FDR-corrected *P* = .001]; right: Cohen *d* = 0.406 [95% CI, 0.159-0.652, FDR-corrected *P* = .03]), left rostral lingual gyrus (Cohen *d* = 0.496; 95% CI, 0.248-0.743; FDR-corrected *P* = .004), and right ventral postcingulate gyrus (Cohen *d* = 0.525; 95% CI, 0.276-0.773; FDR-corrected *P* = .002). The mean SC-FC coupling of 48 anatomical structures (24 per hemisphere; eTable 1 in Supplement 1) was also compared between participants with adolescent MDD and healthy controls (Figure 1D). Significantly increased mean SC-FC coupling was observed in right insula (Cohen *d* = 0.489; 95% CI, 0.241-0.736; FDR-corrected *P* = .002), left precuneus (Cohen *d* = 0.443; 95% CI, 0.196-0.690; FDR-corrected *P* = .007), left inferior parietal lobule (Cohen *d* = 0.545; 95% CI, 0.296-0.793; FDR-corrected *P* < .001), and bilateral posterior superior temporal sulcus (left: Cohen *d* = 0.365 [95% CI, 0.119-0.611, FDR-corrected *P* = .04]; right: Cohen *d* = 0.373 [95% CI, 0.127-0.619, FDR-corrected *P* = .04]).

A control analysis was conducted using age-, sex-, and BMI-matched healthy controls (n = 101) and participants with adolescent MDD (n = 101) (eTable 9 in Supplement 1). Twenty-two subregions showed significant SC-FC coupling increase after FDR correction, including bilateral ventral postcingulate gyrus and bilateral ventromedial parieto-occipital sulcus (Cohen *d* ranged from 0.424 to 0.645). Additionally, the distribution of these regions was highly similar to the distribution in the original analysis (eFigure 9 in Supplement 1).

### Disrupted SC-FC Coupling in MDD Subgroups

**Figure 2.**
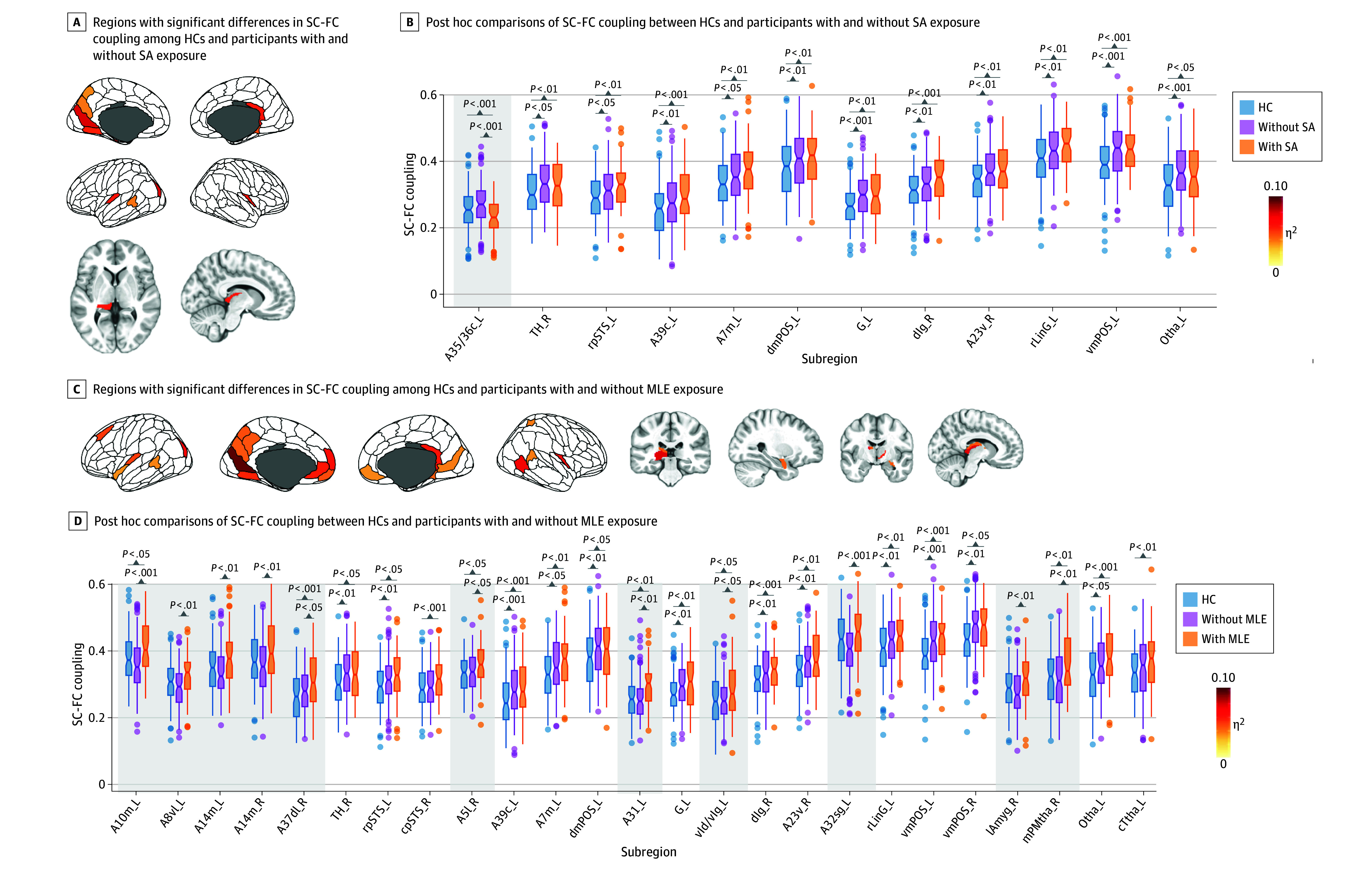
Regional Structural and Functional Connectivity (SC-FC) Coupling Differences Among Clinical Subgroups The upper and lower bounds of the boxes represents the first and third quartile, respectively; horizontal lines, median values; whiskers, 1.5 × of upper and lower bounds of IQRs; and circles above and below boxes, outliers. Partial η^2^ of analysis of variance was mapped on the brain cerebral cortex, thresholding at false discovery rate–corrected *P* < .05. Shaded areas indicate brain regions with subgroup-specific changes in SC-FC coupling. eTable 1 in Supplement 1 provides details on brain subregions. MLE indicates major life event.

To investigate the SC-FC coupling changes associated with different clinical characteristics, we grouped patients according to 2 behavioral symptoms (suicide attempt and NSSI behavior) and 3 adversity exposure (MLEs, childhood trauma, and school bullying) (Figure 2; eFigures 10-11 in Supplement 1). As expected, the increased SC-FC coupling of medioventral occipital cortex, thalamus, precuneus, and cingulate could be replicated well in the 5 adolescent MDD subgroups (eTables 10-19 in Supplement 1). Notably, MDD subgroup–specific SC-FC coupling alterations were detected in bilateral insula, frontal gyrus, parahippocampus, and thalamus.

Specifically, compared with participants without suicide attempts and healthy controls, those with suicide attempts exhibited unique SC-FC coupling decreases in subregions of parahippocampus (partial η^2^ = 0.069; 90% CI, 0.025-0.121; FDR-corrected *P* = .007) (Figure 2A and B; eTables 10-11 in Supplement 1). Compared with participants with NSSI behavior and healthy controls, unique SC-FC coupling increases in subregions of right insula (partial η^2^ = 0.060; 90% CI, 0.019-0.110; FDR-corrected *P* = .01) and left thalamus (partial η^2^ = 0.061; 90% CI, 0.019-0.111; FDR-corrected *P* = .01) were observed in those without NSSI behavior (eFigure 11E-F and eTables 12-13 in Supplement 1). Subgroup variations of SC-FC coupling were most prominent in MDD subgroups with MLE exposure (eFigure 10 in Supplement 1), whereby MLE was associated with significantly higher SC-FC coupling vs without MLE, in 11 brain regions (Figure 2C and D; eTable 17 in Supplement 1), particularly involving frontal-limbic circuit (partial η^2^ ranged from 0.046 to 0.068; FDR-corrected *P* < .05). Moreover, 6 of 11 regions exhibited unique SC-FC coupling increase in MLEs; that is, MDD with MLE exhibited higher regional SC-FC coupling than both MDD without MLE and healthy controls. Collectively, these results suggest that SC-FC coupling delineates subgroup differences precisely in participants with adolescent MDD and complex clinical profiles.

### Correlation Between SC-FC Coupling and MDD Symptoms

**Figure 3.**
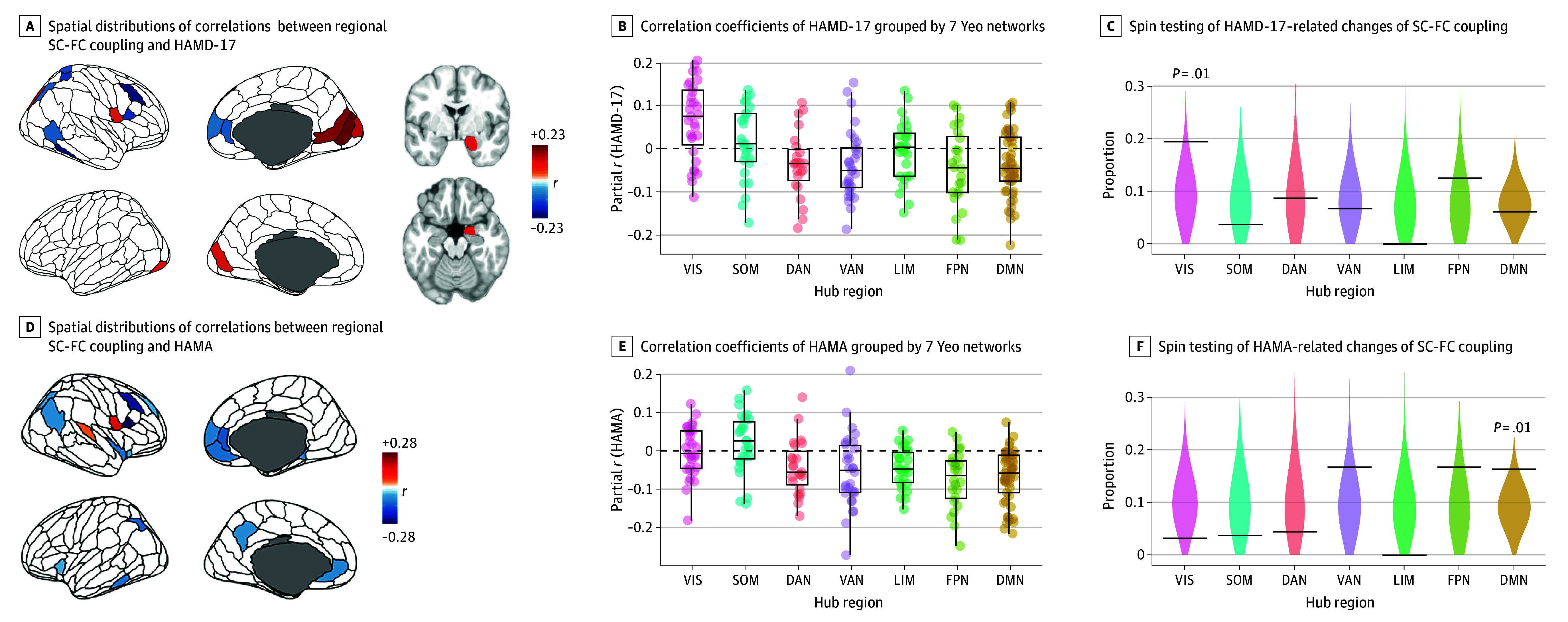
Exploratory Analysis of Associations Between Structural and Functional Connectivity (SC-FC) Coupling and Clinical Symptom Measures Violins represent null distributions of test statistics; horizontal lines in violin plots, empirical test statistics; The upper and lower bounds of the boxes represents the first and third quartile, respectively; horizontal lines, median values; whiskers, 1.5 × of upper and lower bounds of IQRs; and circles above and below boxes, outliers. DAN indicates dorsal attention network; DMN, default mode network; FPN, frontoparietal network; HAMA, 14-item Hamilton Anxiety Rating Scale; HAMD-17, 17- item Hamilton Depression Rating Scale; LIM, limbic network; SOM, somatosensory network; VAN, ventral attention network; VIS, visual network.

The SC-FC coupling changes were correlated with clinical MDD symptoms in HAMD-17 and HAMA (eTables 20-21 in Supplement 1). Using partial Spearman correlation, we found that the maximum direct correlation with HAMD-17 symptoms was in rostral cuneus gyrus (*r* = 0.208; *P* = .008) and the maximum inverse association was in superior frontal gyrus (*r* = −0.223; *P* = .005) (Figure 3A). For HAMA symptoms, the maximum direct association was found in right precentral gyrus (*r* = 0.208; *P* = .008) and the maximum inverse association was found in right ventral inferior frontal gyrus (*r* = −0.271; *P* < .001) (Figure 3D). However, none of the regional correlations were significant after multiple comparison corrections. Spin-based enrichment analysis found that regions with significant symptom associations tended to concentrate in VIS (19.4%; *P* = .01) (Figure 3B and C) and DMN (16.3%; *P* = .01) (Figure 3E and F).

### Sensitivity Analysis

To verify the reproducibility of the findings and examine the association with medication status and sex, we repeated the group difference analyses for participants who were drug-naive and medicated (eFigure 12 in Supplement 1) as well as for male and female participants (eFigure 13 in Supplement 1) separately. The pattern of SC-FC coupling was similar and did not change significantly. Furthermore, we separately repeated the group difference analyses by excluding participants with divorced parents, current smoking status, or current drinking status (eFigure 14 in Supplement 1). The robustness of the main findings to the choice of SC-related matrices and covariate sets were tested (eFigures 15-19 in Supplement 1). As expected, highly consistent results were obtained in most cases.

## Discussion

In this study, we found that adolescent MDD exhibited higher SC-FC coupling, particularly in precuneus, inferior parietal gyrus, insula, and medioventral occipital gyrus as well as pulvinar of thalamus. Subgroup analyses with rigorous sensitivity testing further consolidated the main findings and revealed that participants with adolescent MDD and a suicide attempt showed subgroup-specific SC-FC decoupling in parahippocampal gyrus, whereas participants with adolescent MDD and MLEs exhibited increased SC-FC coupling in the frontal-limbic circuit. To our knowledge, these findings have not been reported, providing insight into the neurobiological basis of adolescent MDD.

### Aberrant Development of SC-FC Coupling in Adolescent MDD

The results highlighted the common increased SC-FC coupling in multiple hub regions of VIS, DMN, and salient network in adolescent MDD. Mounting evidence has supported the altered functional connectivity and topological properties in subregions of the VIS, DMN, and insula cortex in patients with MDD,^36,37,38^ and so did the volumetric and surface morphological alterations in these regions.^14,39^ Unlike previous single-modal imaging studies, the current study integrated 2 different connectional modalities, and the findings suggest that the functional communications in VIS, DMN, and insula cortex are more tethered by anatomical pathways in MDD. This study focused on first-episode adolescent MDD, a reminder that the abnormal developments of anatomical constraints could emerge at an early phase of the depression course. Additional enrichment analysis also supported the association between depressive symptoms and abnormal SC-FC coupling in the VIS and DMN. The disrupted structure-function relationships in sensory and transmodal areas are possibly due to the abnormal progression of myelination in adolescent MDD,^40,41^ which in turn biases the perceptual processing of socially and emotionally relevant visual stimuli.^42,43,44^ Future research could involve examining whether the abnormal SC-FC coupling is a premorbid neurological risk factor of adolescent MDD and establishing effective prognostic markers for subclinical MDD with multimodal brain imaging.

The results also support a crucial anatomically grounded perspective that depression is associated with impaired cross-network dynamics and functional flexibility. Convergent evidence has shown the substantial alignment between SC-FC coupling and hierarchies of cortical function, cytoarchitecture, and evolutionary expansion, suggesting that flexible functional reconfiguration and effective multisensory integration rely on less-anatomical bounded functional networks.^32,45,46,47^ In contrast, excessive anatomical constraints for functional communication could induce difficulties in functional reconfigurations and abnormal recruitment of cognitive resources.^32,47,48^ Patients with MDD require greater cognitive resources to modulate the activity of VIS^49,50^ and exhibit aberrant cross-network dynamics among DMN, central executive network, and salient network.^51^ The impaired functional dynamics in adolescent MDD may be attributed to the disrupted structure-function relationships in insula, as it is a hub region responsible for modulating cross-network interactions.^52,53^ More specifically, the excessive anatomical constraints for functional communication between insula and other regions may play a role in additional switching costs for adolescent MDD to cycle out of internal emotional states to attend to salient task-relevant stimuli.^51^ However, the association between SC-FC coupling increase and inflexible functional reconfigurations in adolescent MDD warrants further examination.

### SC-FC Coupling Alterations in Adolescent MDD Subgroups

We identified some subgroup-specific SC-FC coupling changes via extensive and dedicated subgroup analyses. Particularly, participants with a suicide attempt exhibited unique SC-FC decoupling in parahippocampal gyrus compared with participants without a suicide attempt and healthy controls. Similar discovery was reported between SC-FC decoupling and suicide attempt in patients with bipolar disorder with a current major depressive episode.^23^ The results of the present study further suggest that the parahippocampal SC-FC decoupling could be a prominent neurophysiological feature for depression-related suicidal behaviors. Given the critical role of parahippocampal gyrus in human prospective brain,^54^ we conjecture that the disrupted structure-function relationship in parahippocampal gyrus may play a role in the impaired personal future representation in adolescent MDD,^54,55,56^ which reinforces the preference toward less future-minded behavior during a suicidal crisis.^57^

Additionally, MDD with MLE exhibited the most unique pattern of SC-FC coupling changes. Compared with no MLE, more distinctive SC-FC coupling increases were identified in regions implicated in the frontal-limbic circuit in MLE, including orbitofrontal gyrus and amygdala. Frontal-limbic circuit is responsible for emotion regulation and plays a central role in the psychopathology of depression.^13,58^ From this perspective, the findings have expanded on previous research on the aberrant frontal-limbic function in depression and have underscored the profound implications of exposure to stressful life events for frontal-limbic development during adolescence.^59^ Future studies may explore the neuropathological heterogeneity of adolescent MDD by integrating multimodal brain features with more medication status and external stressors.

### Limitations

Several limitations should be noted. First, although the study included relatively large multimodal neuroimaging data of adolescent MDD, these data might still be insufficient to fully reveal the subgroup-specific variance of SC-FC coupling given the high co-occurrence of the examined clinical characteristics and lack of a control group with risk exposure in these samples. Additionally, considering the differences in age and sex between participants with MDD and healthy controls, the samples may have healthy volunteer bias^60,61^; to what extent this bias might affect the generalizability of the findings requires further examination.^62^ Second, accurately resolving the structure-function relationship of human brain is challenging, particularly given the precision limit of whole-brain diffusion imaging.^63^ The definition of regional SC-FC coupling in the present study provides a concise way to map the structure-function relationship, but it may sacrifice the fMRI temporal resolution and the edge resolution of traditional connectivity analysis.^46,48^ Future research can incorporate novel deep learning approaches to adaptively map fine-grained structure-function relationship. Third, although we identified the pattern of abnormal SC-FC coupling in adolescent MDD, it remains unclear whether this pattern is a premorbid neurophysiological risk factor for the emergence of depression. Future studies could address this gap by evaluating the developmental changes of SC-FC coupling in a longitudinal sample composed of individuals at low or high risk for depression.

## Conclusions

This cross-sectional study found commonly increased SC-FC coupling in adolescent MDD that primarily involved hub regions of the DMN and VIS as well as subregions in insula. Subgroup analyses highlighted the heterogeneous SC-FC coupling alterations in adolescent MDD, especially in participants with suicide attempt and MLE exposure. These results enrich knowledge of the aberrant brain SC-FC coupling in the psychopathology of adolescent MDD, underscoring the vulnerability of frontal-limbic SC-FC coupling to external stressors and the parahippocampal coupling in shaping future-minded behavior.

## Notes

Supplement 1. **eFigure 1.** Study Design and Analytical Procedure **eFigure 2.** The Pipeline of Defining Seed and Target Regions for Probabilistic Tractography **eFigure 3.** Principle Component Analysis and the Consistency of Components Across Participants **eFigure 4.** The Pattern and Composition of PC1 **eFigure 5.** The Pattern and Composition of PC2 **eFigure 6.** The Pattern and Composition of PC3 **eFigure 7.** The Pattern and Composition of PC4 **eFigure 8.** Average Contributions of Each PC for Predicting Regional FC in Healthy Subjects **eFigure 9.** Control Analysis Using a Matched Healthy and MDD Sample **eFigure 10.** Subgroup Differences in SC−FC Coupling at Each Brain Region **eFigure 11.** Regional SC−FC Coupling Differences Among Subgroups With Different Clinical Characteristics **eFigure 12.** Sensitivity Analysis of the Effects Owing to the Medication Status on the Main Group Comparison Results **eFigure 13.** Sensitivity Analysis of the Effects Owing to the Gender on the Main Group Comparison Results **eFigure 14.** Sensitivity Analysis of the Effects Owing to Other Behavioral and Environmental Factors on the Main Group Comparison Results **eFigure 15.** Distributions of the SC−FC Coupling Calculated by Different SC−Related Matrices **eFigure 16.** Distributions of the SC−FC Coupling Calculated by Selected Combinations of SC−Related Matrices **eFigure 17.** Correlations Between the Group Differences of SC−FC Coupling Calculated Based on All SC−Related Matrices and on Single Matrix **eFigure 18.** Correlations Between the Group Effect Sizes of SC−FC Coupling Calculated Based on All SC−Related Matrices and on Selected Subsets of Matrices **eFigure 19.** The Effect of Different Covariate Sets on the Group-Differed SC-FC Coupling Patterns
eMethods.
**eTable 1.** The Regions That Defined in Brainnetome Atlas at Different Levels **eTable 2.** Statistical Analysis of Sociodemographic and Clinical Characteristics Among Heathy Controls Without Childhood Trauma (HCs), Adolescent MDD With and Without Childhood Trauma (CT+/CT−) **eTable 3.** Statistical Analysis of Sociodemographic and Clinical Characteristics Among Heathy Controls Without School Bullying (HCs), Adolescent MDD With and Without School Bullying (SB+/SB−) **eTable 4.** Statistical Analysis of Sociodemographic and Clinical Characteristics Among Heathy Controls Without Major Life Events (HCs), Adolescent MDD With and Without Major Life Events (MLE+/MLE−) **eTable 5.** Statistical Analysis of Sociodemographic and Clinical Characteristics Among Heathy Controls Without Suicidal Attempt (HCs), Adolescent MDD With and Without Suicide Attempt (SA+/SA−) **eTable 6.** Statistical Analysis of Sociodemographic and Clinical Characteristics Among Heathy Controls Without Non−Suicidal Self−Injurious Behavior (HC), Adolescent MDD With and Without Non−Suicidal Self−Injurious Behavior (NSSI+/NSSI−) **eTable 7.** Statistical Analysis of Differences Between MDD and HCs **eTable 8.** The Network Definition in Brainnetome Atlas **eTable 9.** Demographic Characteristics of the Matched Sample **eTable 10.** Statistical Analyses of SC−FC Coupling Differences Among Healthy Controls, Adolescent MDD With and Without Suicidal Attempt (SA+ and SA−) **eTable 11.** Post−Hoc Comparisons of SC−FC Coupling Differences Among Healthy Controls, Adolescent MDD With and Without Suicidal Attempt (SA+ and SA−) **eTable 12.** Statistical Analyses of SC−FC Coupling Differences Among Healthy Controls, Adolescent MDD With and Without Non−Suicidal Self−Injurious Behaviors (NSSI+ and NSSI−) **eTable 13.** Post−Hoc Comparisons of SC−FC Coupling Differences Among Healthy Controls, Adolescent MDD With and Without Non−Suicidal Self−Injurious Behaviors (NSSI+ and NSSI−) **eTable 14.** Statistical Analyses of SC−FC Coupling Differences Among Healthy Controls, Adolescent MDD With and Without Childhood Trauma (CT+ and CT−) **eTable 15.** Post−Hoc Comparisons of SC−FC Coupling Differences Among Healthy Controls, Adolescent MDD With and Without Childhood Trauma (CT+ and CT−) **eTable 16.** Statistical Analyses of SC−FC Coupling Differences Among Healthy Controls, Adolescent MDD With and Without Major Life Events (MLE+ and MLE−) **eTable 17.** Post−Hoc Comparisons of SC−FC Coupling Differences Among Healthy Controls, Adolescent MDD With and Without Major Life Events (MLE+ and MLE−) **eTable 18.** Statistical Analyses of SC−FC Coupling Differences Among Healthy Controls, Adolescent MDD With and Without Major School Bullying (SB+ and SB−) **eTable 19.** Post−Hoc Comparisons of SC−FC Coupling Differences Among Healthy Controls, Adolescent MDD With and Without School Bullying (SB+ and SB−) **eTable 20.** Partial Spearman Correlation Coefficients Between SC−FC Coupling and HAMD-17 **eTable 21.** Partial Spearman Correlation Coefficients Between SC−FC Coupling and HAMA

Supplement 2.
Data Sharing Statement
