# Supplementary material for: Reconfiguration of Structural and Functional Connectivity Coupling in Patient Subgroups With Adolescent Depression
Source: JAMA Netw Open. 2024 Mar 12;7(3):e241933. doi: 10.1001/jamanetworkopen.2024.1933 (PMC10933730; doi:10.1001/jamanetworkopen.2024.1933)
Supplement: Supplement 1. — eFigure 1. Study Design and Analytical Procedure eFigure 2. The Pipeline of Defining Seed and Target Regions for Probabilistic Tractography eFigure 3. Principle Component Analysis and the Consistency of Components Across Participants eFigure 4. The Pattern and Composition of PC1 eFigure 5. The Pattern and Composition of PC2 eFigure 6. The Pattern and Composition of PC3 eFigure 7. The Pattern and Composition of PC4 eFigure 8. Average Contributions of Each PC for Predicting Regional FC in Healthy Subjects eFigure 9. Control Analysis Using a Matched Healthy and MDD Sample eFigure 10. Subgroup Differences in SC−FC Coupling at Each Brain Region eFigure 11. Regional SC−FC Coupling Differences Among Subgroups With Different Clinical Characteristics eFigure 12. Sensitivity Analysis of the Effects Owing to the Medication Status on the Main Group Comparison Results eFigure 13. Sensitivity Analysis of the Effects Owing to the Gender on the Main Group Comparison Results eFigure 14. Sensitivity Analysis of the Effects Owing to Other Behavioral and Environmental Factors on the Main Group Comparison Results eFigure 15. Distributions of the SC−FC Coupling Calculated by Different SC−Related Matrices eFigure 16. Distributions of the SC−FC Coupling Calculated by Selected Combinations of SC−Related Matrices eFigure 17. Correlations Between the Group Differences of SC−FC Coupling Calculated Based on All SC−Related Matrices and on Single Matrix eFigure 18. Correlations Between the Group Effect Sizes of SC−FC Coupling Calculated Based on All SC−Related Matrices and on Selected Subsets of Matrices eFigure 19. The Effect of Different Covariate Sets on the Group-Differed SC-FC Coupling Patterns eMethods. eTable 1. The Regions That Defined in Brainnetome Atlas at Different Levels eTable 2. Statistical Analysis of Sociodemographic and Clinical Characteristics Among Heathy Controls Without Childhood Trauma (HCs), Adolescent MDD With and Without Childhood Trauma (CT+/CT−) eTable 3. Statistical A [file jamanetwopen-e241933-s001.pdf]

# Supplemental Online Content

Xu M, Li X, Teng T, et al. Reconfiguration of structural and functional connectivity coupling in patient subgroups with adolescent depression. *JAMA Netw Open*. 2024;7(3):e241933. doi:10.1001/jamanetworkopen.2024.1933

**eFigure 1.** Study Design and Analytical Procedure
**eFigure 2.** The Pipeline of Defining Seed and Target Regions for Probabilistic Tractography
**eFigure 3.** Principle Component Analysis and the Consistency of Components Across Participants
**eFigure 4.** The Pattern and Composition of PC1
**eFigure 5.** The Pattern and Composition of PC2
**eFigure 6.** The Pattern and Composition of PC3
**eFigure 7.** The Pattern and Composition of PC4
**eFigure 8.** Average Contributions of Each PC for Predicting Regional FC in Healthy Subjects
**eFigure 9.** Control Analysis Using a Matched Healthy and MDD Sample
**eFigure 10.** Subgroup Differences in SC−FC Coupling at Each Brain Region
**eFigure 11.** Regional SC−FC Coupling Differences Among Subgroups With Different Clinical Characteristics
**eFigure 12.** Sensitivity Analysis of the Effects Owing to the Medication Status on the Main Group Comparison Results
**eFigure 13.** Sensitivity Analysis of the Effects Owing to the Gender on the Main Group Comparison Results
**eFigure 14.** Sensitivity Analysis of the Effects Owing to Other Behavioral and Environmental Factors on the Main Group Comparison Results
**eFigure 15.** Distributions of the SC−FC Coupling Calculated by Different SC−Related Matrices
**eFigure 16.** Distributions of the SC−FC Coupling Calculated by Selected Combinations of SC−Related Matrices
**eFigure 17.** Correlations Between the Group Differences of SC−FC Coupling Calculated Based on All SC−Related Matrices and on Single Matrix
**eFigure 18.** Correlations Between the Group Effect Sizes of SC−FC Coupling Calculated Based on All SC−Related Matrices and on Selected Subsets of Matrices
**eFigure 19.** The Effect of Different Covariate Sets on the Group-Differed SC-FC Coupling Patterns
**eMethods.**
**eTable 1.** The Regions That Defined in Brainnetome Atlas at Different Levels
**eTable 2.** Statistical Analysis of Sociodemographic and Clinical Characteristics Among Heathy Controls Without Childhood Trauma (HCs), Adolescent MDD With and Without

© 2024 Xu M et al. *JAMA Network Open*

Childhood Trauma (CT+/CT−)
**eTable 3.** Statistical Analysis of Sociodemographic and Clinical Characteristics Among Heathy Controls Without School Bullying (HCs), Adolescent MDD With and Without School Bullying (SB+/SB−)
**eTable 4.** Statistical Analysis of Sociodemographic and Clinical Characteristics Among Heathy Controls Without Major Life Events (HCs), Adolescent MDD With and Without Major Life Events (MLE+/MLE−)
**eTable 5.** Statistical Analysis of Sociodemographic and Clinical Characteristics Among Heathy Controls Without Suicidal Attempt (HCs), Adolescent MDD With and Without Suicide Attempt (SA+/SA−)
**eTable 6.** Statistical Analysis of Sociodemographic and Clinical Characteristics Among Heathy Controls Without Non−Suicidal Self−Injurious Behavior (HC), Adolescent MDD With and Without Non−Suicidal Self−Injurious Behavior (NSSI+/NSSI−)
**eTable 7.** Statistical Analysis of Differences Between MDD and HCs
**eTable 8.** The Network Definition in Brainnetome Atlas
**eTable 9.** Demographic Characteristics of the Matched Sample
**eTable 10.** Statistical Analyses of SC−FC Coupling Differences Among Healthy Controls, Adolescent MDD With and Without Suicidal Attempt (SA+ and SA−)
**eTable 11.** Post−Hoc Comparisons of SC−FC Coupling Differences Among Healthy Controls, Adolescent MDD With and Without Suicidal Attempt (SA+ and SA−)
**eTable 12.** Statistical Analyses of SC−FC Coupling Differences Among Healthy Controls, Adolescent MDD With and Without Non−Suicidal Self−Injurious Behaviors (NSSI+ and NSSI−)
**eTable 13.** Post−Hoc Comparisons of SC−FC Coupling Differences Among Healthy Controls, Adolescent MDD With and Without Non−Suicidal Self−Injurious Behaviors (NSSI+ and NSSI−)
**eTable 14.** Statistical Analyses of SC−FC Coupling Differences Among Healthy Controls, Adolescent MDD With and Without Childhood Trauma (CT+ and CT−)
**eTable 15.** Post−Hoc Comparisons of SC−FC Coupling Differences Among Healthy Controls, Adolescent MDD With and Without Childhood Trauma (CT+ and CT−)
**eTable 16.** Statistical Analyses of SC−FC Coupling Differences Among Healthy Controls, Adolescent MDD With and Without Major Life Events (MLE+ and MLE−)
**eTable 17.** Post−Hoc Comparisons of SC−FC Coupling Differences Among Healthy Controls, Adolescent MDD With and Without Major Life Events (MLE+ and MLE−)
**eTable 18.** Statistical Analyses of SC−FC Coupling Differences Among Healthy Controls, Adolescent MDD With and Without Major School Bullying (SB+ and SB−)
**eTable 19.** Post−Hoc Comparisons of SC−FC Coupling Differences Among Healthy Controls, Adolescent MDD With and Without School Bullying (SB+ and SB−)
**eTable 20.** Partial Spearman Correlation Coefficients Between SC−FC Coupling and HAMD-17
**eTable 21.** Partial Spearman Correlation Coefficients Between SC−FC Coupling and HAMA
© 2024 Xu M et al. *JAMA Network Open*

This supplemental material has been provided by the authors to give readers additional information about their work.

© 2024 Xu M et al. *JAMA Network Open*

**eFigure 1.** Study design and analytical procedure

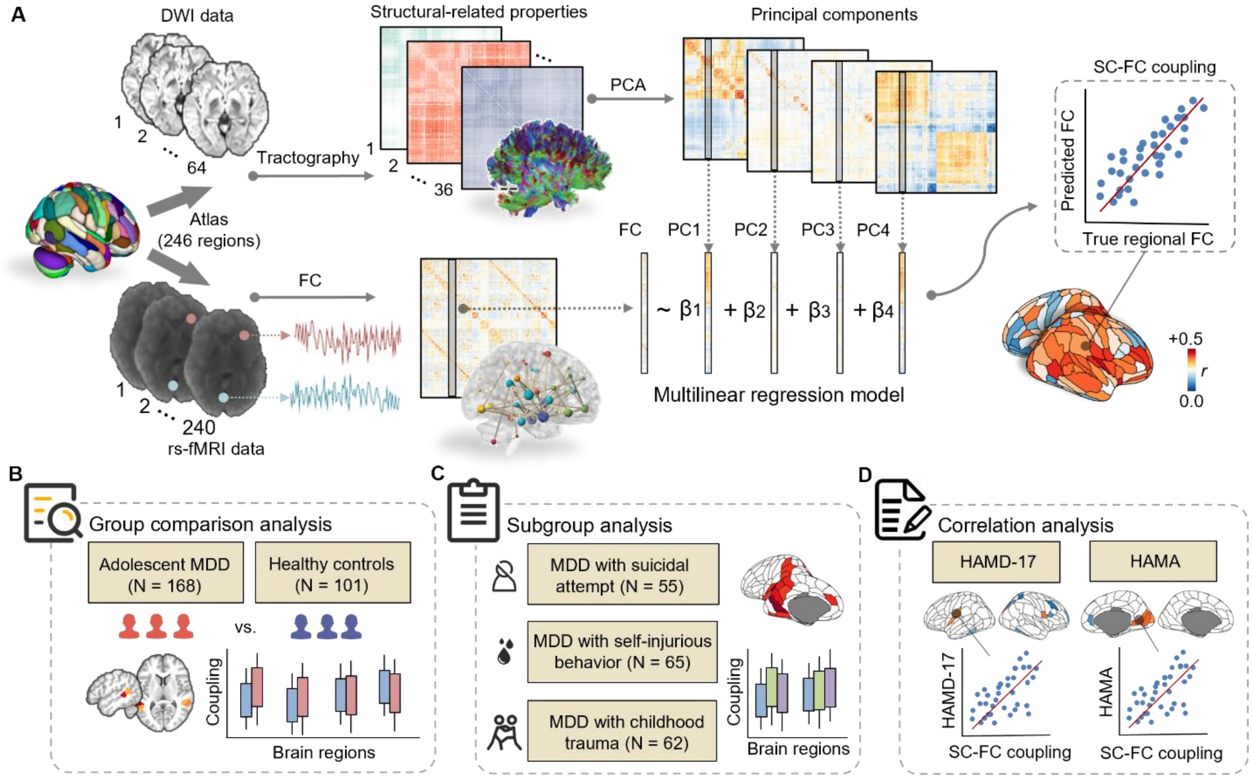


**eFigure 1. Study design and analytical procedure.** (**A**) The pipeline for calculating the SC-FC coupling of each brain region. First, function connectivity and structure connectivity with 246 brain parcels were calculated for each participant. Second, a suite of structural properties was calculated based on the structure connectivity of each participant. Third, PCA was performed on all 36 structural properties, and the top-n components that collectively accounted for more than 80% of variance were selected. Finally, a multilinear regression model was established based on regional functional profiles and structural profiles of the top-n components, and the SC-FC coupling was measured as the Pearson correlation coefficient of the multilinear model. (**B**) The group difference analysis of the differences of SC-FC coupling in each brain region between adolescent MDD and healthy controls. (**C**) Analysis of the SC-FC coupling variations between MDD subgroups with different clinical characters and healthy controls. (**D**) Correlation analysis between SC-FC coupling and clinical measures, *i.e.*, HAMD-17, HAMA. HAMD-17, 17-item Hamilton Depression Scale; HAMA, Hamilton Anxiety Scale; rs-fMRI, resting-state functional magnetic resonance imaging; DWI, diffusion-weighted imaging; PCA, principal component analysis.

© 2024 Xu M et al. *JAMA Network Open*

**eFigure 2.** The pipeline of defining seed and target regions for probabilistic tractography

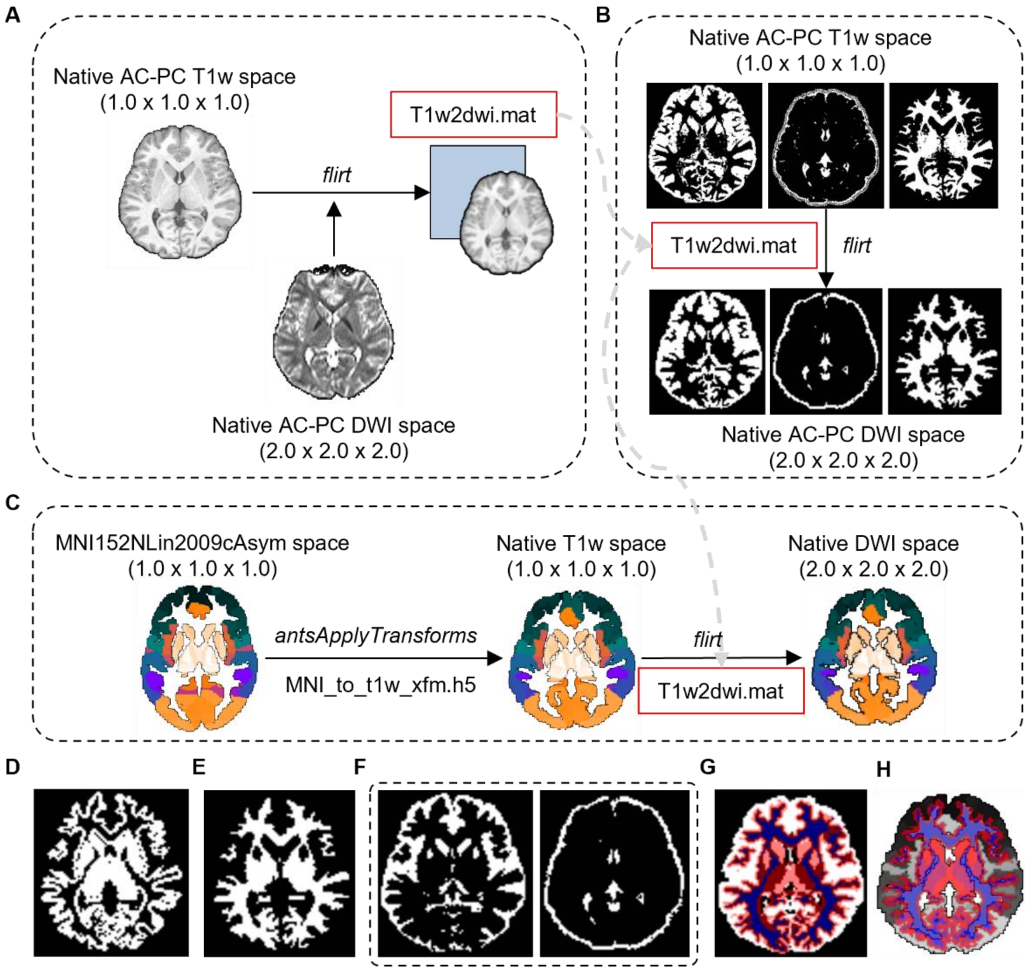


**eFigure 2. The pipeline of defining seed and target regions for probabilistic tractography.** (**A**) The preprocessed T1w images are registered to the resampled preprocessed DWI reference image ($b$ = 0) through FSL's *flirt*, resulting a transformation matrix, *i.e.*, T1w2dwi.mat (from native T1w space to native DWI space). (**B**) GM mask, WM mask, CSF mask are registered to native DWI space through FSL's *flirt* with initialized transformation matrix, *i.e.*, T1w2dwi.mat, thresholding at 0.5. (**C**) The brain atlas is registered to native DWI space through a two−step manner. (**D**) The whole−brain seed mask (in native DWI space) for ROI−by−ROI tractography. (**E**) An example of WM mask in native DWI space. (**F**) Examples of superficial gray mask (the left) and CSF mask (the right). (**G**) Overlay the seed mask on GM mask and WM mask. The red regions correspond to seed mask, the white regions correspond to GM mask, and the blue regions correspond to WM mask. (**H**) Overlay lay the seed mask on the Brainnetome atlas, illustrating the seed voxels of each brain regions.

© 2024 Xu M et al. *JAMA Network Open*

**eFigure 3.** Principle component analysis and the consistency of components across participants

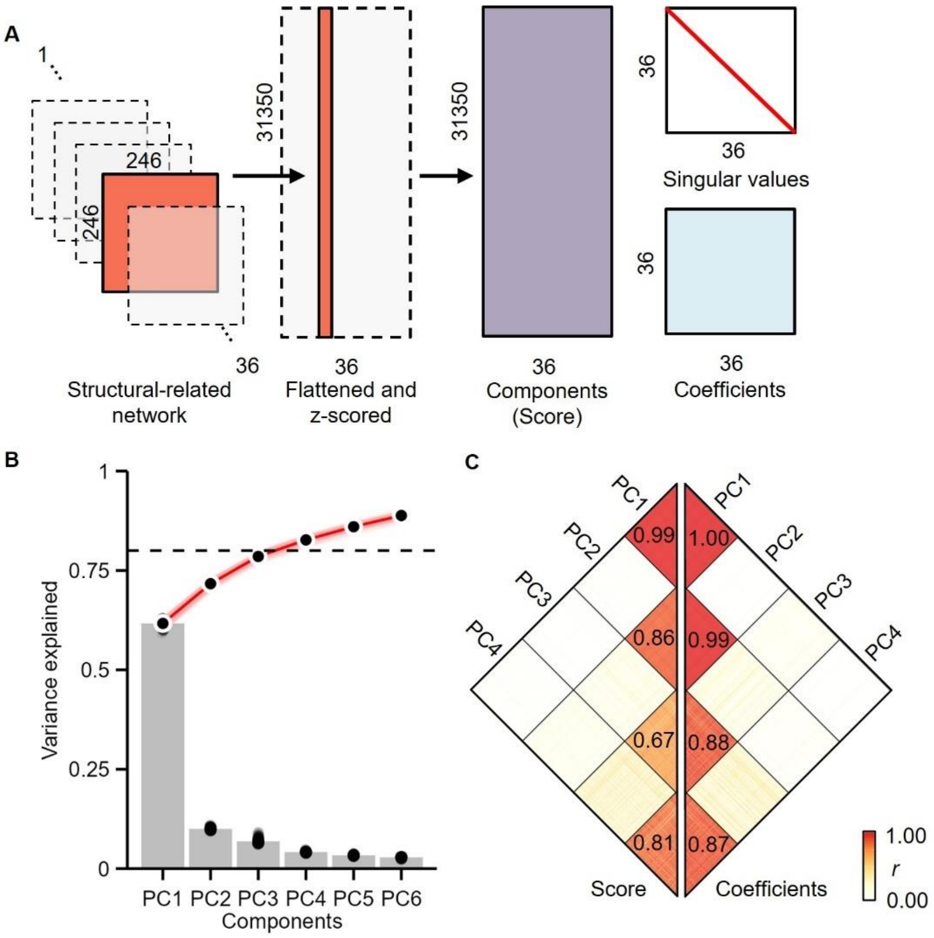


**eFigure 3. Principle component analysis and the consistency of components across participants.** (**A**) Principal component analysis is performed on flattened 36 structural−related networks. Specifically, each 246×246 symmetrical structural−related matrix is flattened and z−scored, forming a 31350×36 structural feature matrix for each subject. Then, this individual feature matrix is decomposed into (31350×36) principal components (PCs), (36×36) coefficients, (36×36 diagonal) singular values. (**B**) the variance explained of the top 6 principal components. The saturated red line illustrates the mean accumulative variance explained, while light red lines illustrate accumulative explained variance for each participant. The horizontal black dashed line indicates expected 80% variance. Grey bars correspond to the mean variance accounted by each PC, while dark dots correspond to the variance explained by each PC for each participant. Top 4 PCs explain 61.6 ± 0.7, 10.0 ± 0.3, 6.8 ± 0.5, 4.2 ± 0.2 percent of variance, respectively, and collectively explained more than 80% of variance across all participants. (**C**) Similarity analysis of the top 4 PCs. The left part corresponds to the similarity of top 4 PCs, while the right part corresponds to the similarity of coefficients from the top 4 PCs. The similarity is measured as the absolute Pearson correlational coefficient. PC, principal components.

**eFigure 4.** The pattern and composition of PC1

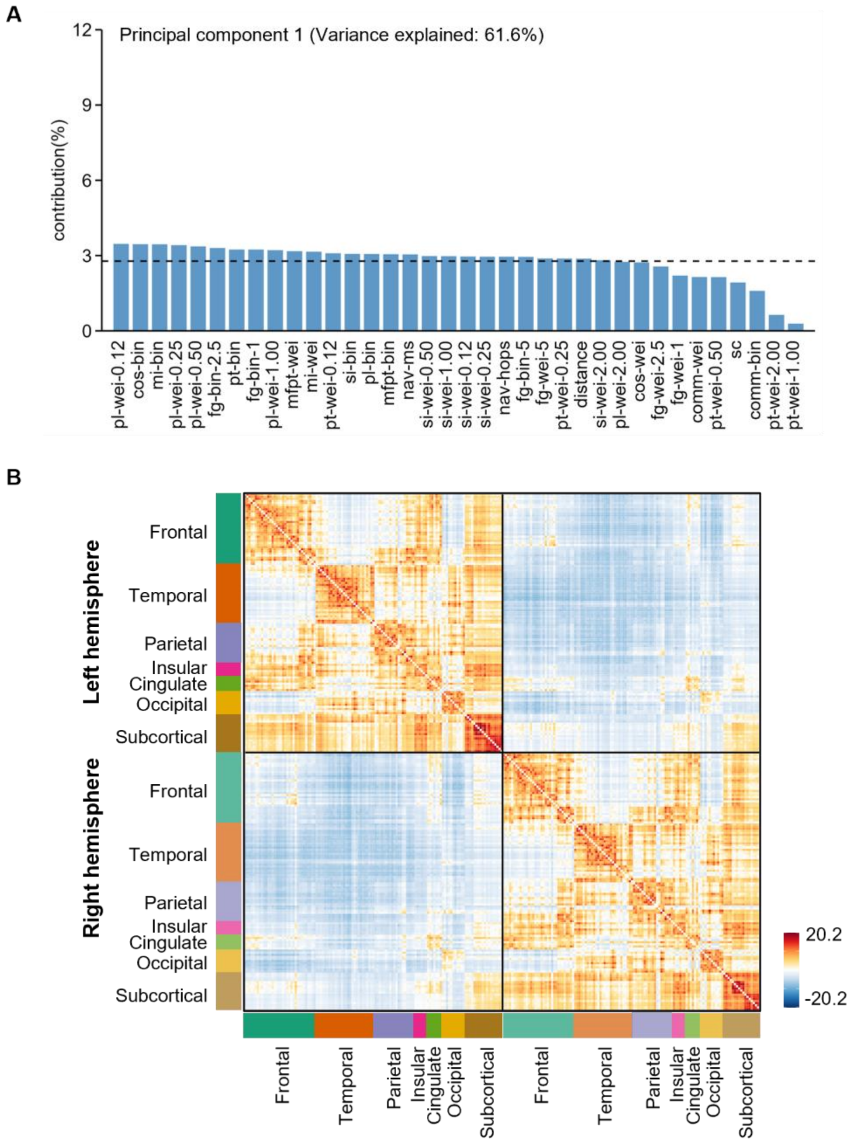


**eFigure 4. The pattern and composition of PC1.** (**A**) Average contributions of 36 structural–related matrices to PC1**.**The contribution is measured as normalized absolute value of PCA coefficients. The dashed line indicates the expected average contributions (1/36). For details about SC-related matrices, please refer to eMethods. Although each structural feature largely contributes equally to PC1, weighted path length and binarized topological similarities (*i.e.*, cosine similarity and matching index), all of which heavily depend on the skeleton and strength of structure connectivity, are contributed slightly more, since their contributions are all ranked in the top 5. (**B**) The connectivity patterns of PC1. Interestingly, PC1 presents a prominent lobular-level modularized organization, that is, regions that belong to the same lobe are more connected. Therefore, we suggest PC1 reflects the general modularized organization of brain, which are mainly driven by shortest anatomical path (that is, the path with most strongly inter-connected nodes) and node similarity.

© 2024 Xu M et al. *JAMA Network Open*

**eFigure 5.** The pattern and composition of PC2

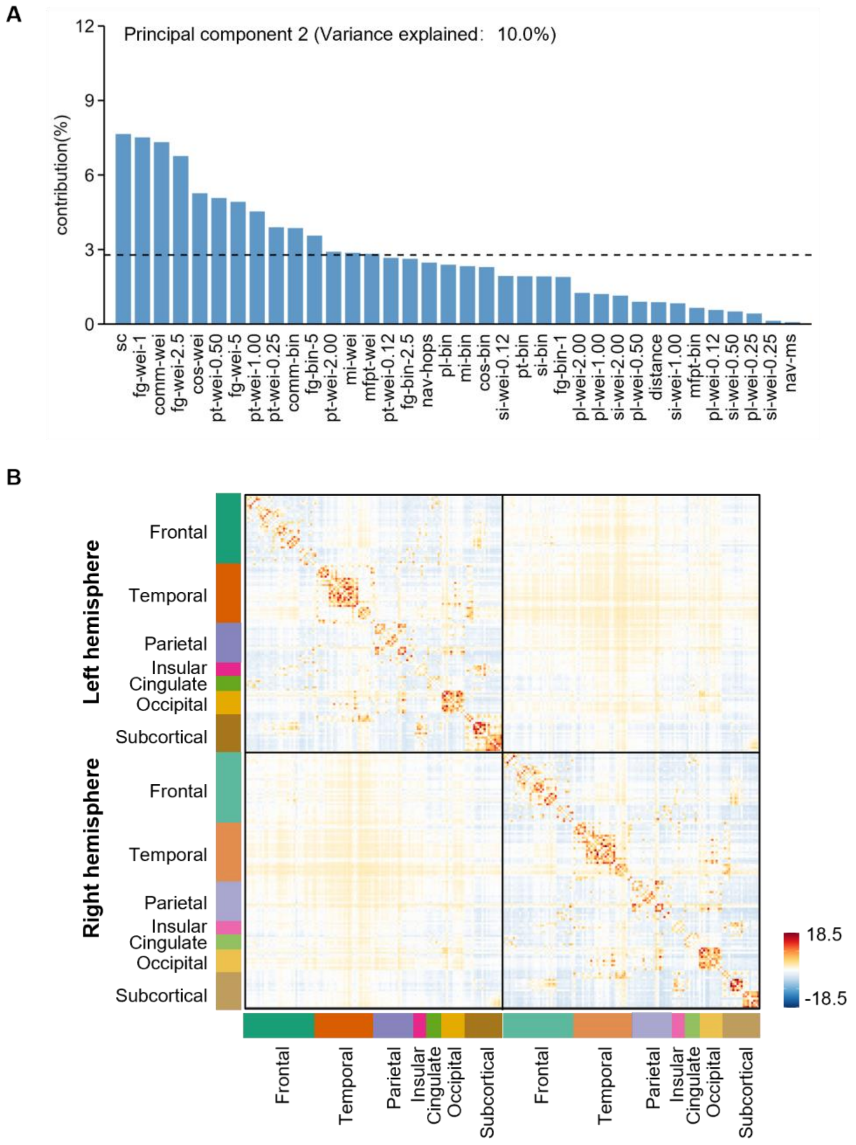


**eFigure 5. The pattern and composition of PC2.** (**A**) Average contributions of 36 structural–related matrices to PC2**.**The contribution is measured as normalized absolute value of PCA coefficients. The dashed line indicates the expected average contributions (1/36). For details about SC-related matrices, please refer to eMethods. The most contributed structural features are connectivity strength, communicability, and flow graph with short time range (*i.e.*, *t* = 1.0). (**B**) The connectivity patterns of PC2. Differing from PC1 significantly, PC2 presents a diagonal distribution, indicating a high locality connectivity. Accordingly, we consider PC2 mainly captures the local signal transmission pattern among brain regions dominated by direct anatomical connection.

**eFigure 6.** The pattern and composition of PC3

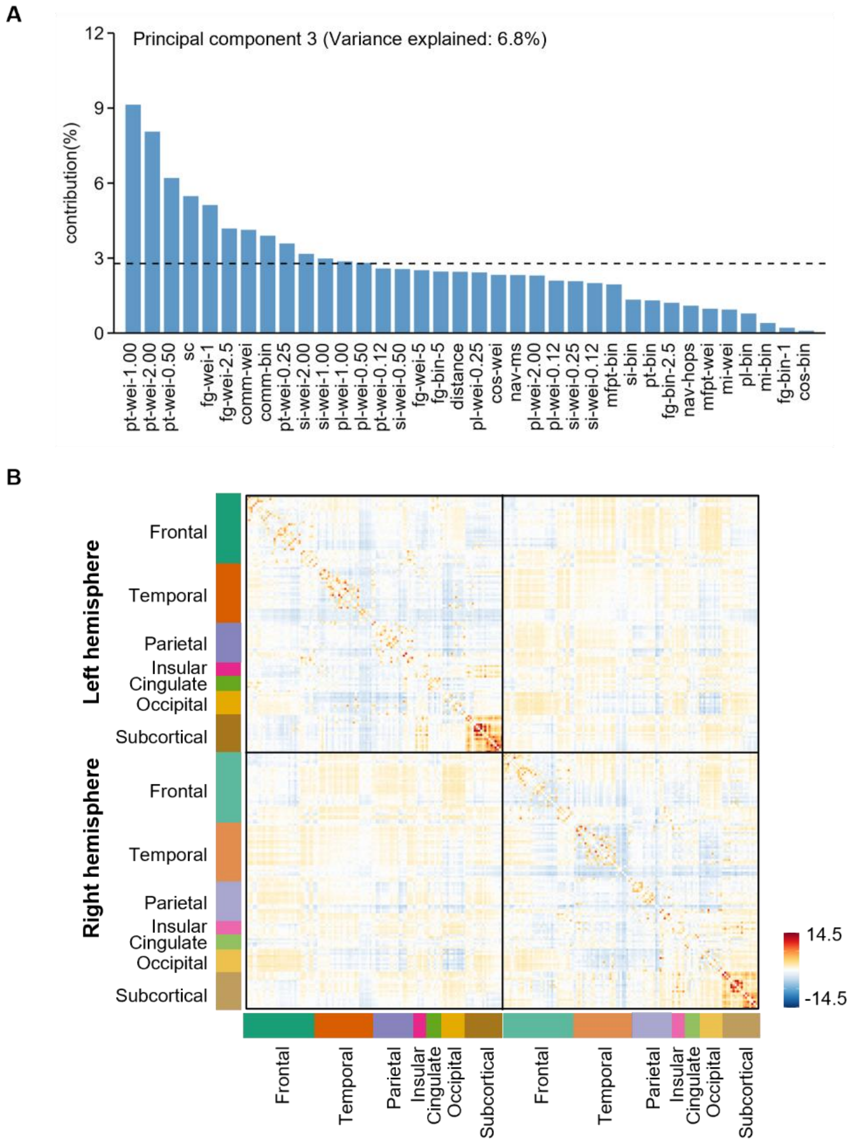


**eFigure 6. The pattern and composition of PC3.** (**A**) Average contributions of 36 structural–related matrices to PC3**.**The contribution is measured as normalized absolute value of PCA coefficients. The dashed line indicates the expected average contributions (1/36). For details about SC-related matrices, please refer to eMethods. The most contributed structural features for PC3 are path transitivity, which measure the density of local detours along the shortest paths[1]. (**B**) The connectivity patterns of PC3. PC3 is quite similar with PC2, with only subtle differences along the diagonal, *i.e.*, the weights between the source region and several near-most region is weaker than PC2. Therefore, we consider that PC3 captures another local information passing strategy driven by path transitivity, which may act as a compensation way for the local information communication relying on direct anatomical connections.

**eFigure 7.** The pattern and composition of PC4

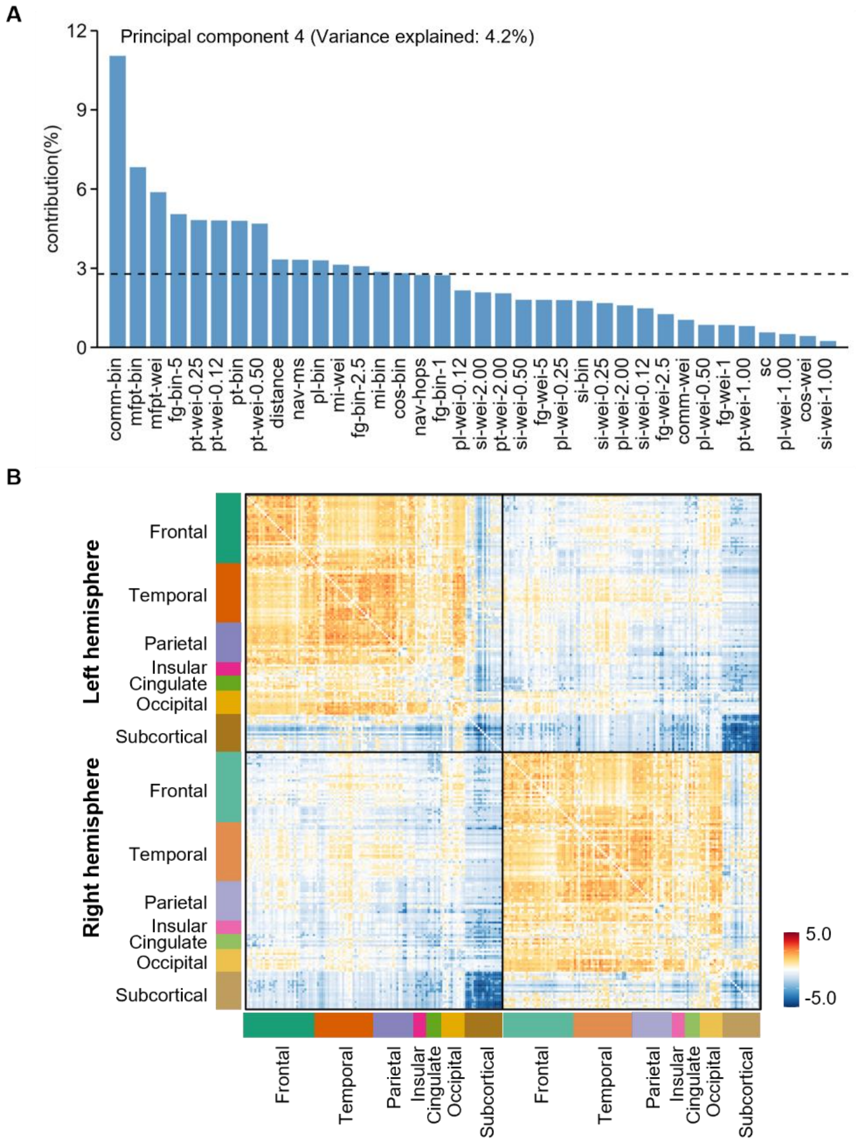


**eFigure 7. The pattern and composition of PC4.** (**A**) Average contributions of 36 structural–related matrices to PC4**.**The contribution is measured as normalized absolute value of PCA coefficients. The dashed line indicates the expected average contributions (1/36). For details about SC-related matrices, please refer to eMethods. The most contributed structural features for PC4 are communicability and mean first passage time based on the binarized structural connectivity. (**B**) The connectivity patterns of PC4. As PC4 largely relies on the binarized structural connectivity (*i.e.*, the information about the strength of direct anatomical connectivity is deprecated), the modularity within hemisphere and the local connectivity of each brain region are not prominent as PC1-3. However, we can find the weights of PC4 are differed among cortico-cortical connection, cortico-subcortical connection, and inter-hemisphere subcortical connection. Therefore, we consider that PC4 captures some patterns for long-range/inter-hemisphere macroscale communication in human brain, which may largely rely on subcortical structure to relay and modulate the incoming information.

© 2024 Xu M et al. *JAMA Network Open*

**eFigure 8.** Average contributions of each PC for predicting regional FC in healthy subjects

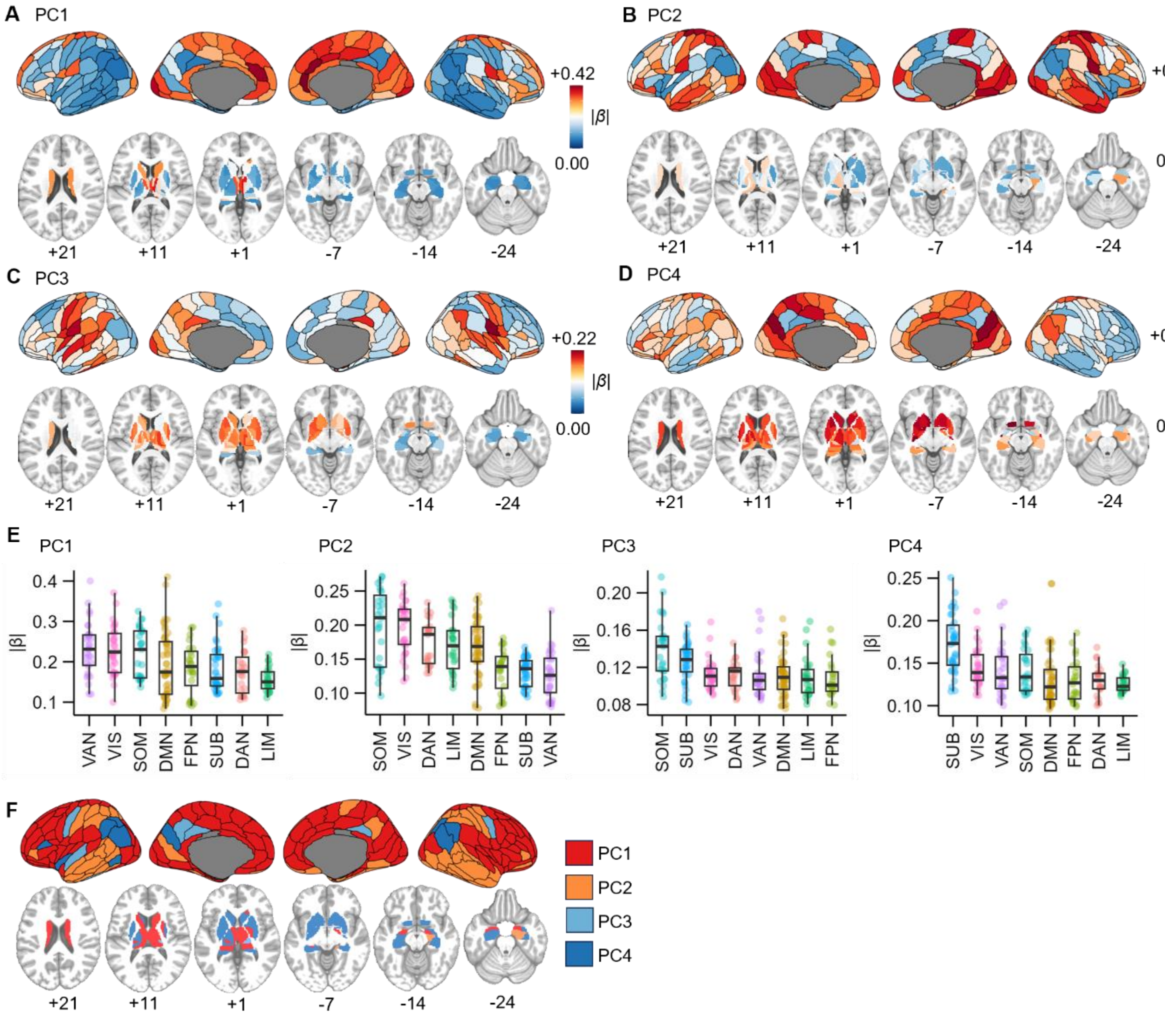


**eFigure 8. Average contributions of each PC for predicting regional FC in healthy subjects.** The contribution is measured as the absolute value of standardized beta weights in multilinear regression models which were built for fitting regional FC. Overall, the contributions of each PC for FC prediction are heterogenous. (**A**) Contributions of PC1 for predicting regional FC. PC1 contributes most for predicting the FC of frontal and superior parietal areas around the cortex middle line. (**B**) Contributions of PC2 for predicting regional FC. PC2 contributes relatively large for predicting the FC of paracentral areas and temporal areas. (**C**) Contributions of PC3 for predicting regional FC. PC3 contributes relatively large for predicting the FC of paracentral areas and insular areas. (**D**) Contributions of PC4 for predicting regional FC. PC4 contributes most for inferior parietal lobe and subcortical areas. (**E**) The distribution of contributions at the network level. Each point corresponds to a brain region. In each boxplot, the "box" indicates interquartile range (IQR), the horizontal bar indicated the median value, and the whiskers include points that are within 1.5×IQR of upper and lower bounds of the IQR. The contributions of the top 4 PCs are varied across brain networks (One-way ANOVA; PC1: $F_{(7,238)} = 5.56$, $p < 0.001$; PC2 : $F_{(7,238)} = 16.89$, $p < 0.001$; PC3 : $F_{(7,238)} = 7.06$, $p < 0.001$ ; PC4 : $F_{(7,238)} = 10.16$, $p < 0.001$). Specifically, PC1 contributes most for predicting FCs of regions in ventral attention network; PC2 and PC3 both contribute largely for predicting FCs of regions in somatomotor network; PC4 contributes largely for predicting FCs of subcortical regions. All PCs contribute largely for predicting FCs of regions in visual network. (**F**) The most contributive

PC for each brain region. The results suggest that the PC1 contributes the FC prediction largest for most brain regions, while other PCs have some region-specific contributions for FC prediction.

© 2024 Xu M et al. *JAMA Network Open*

**eFigure 9.** Control analysis using matched healthy and MDD sample

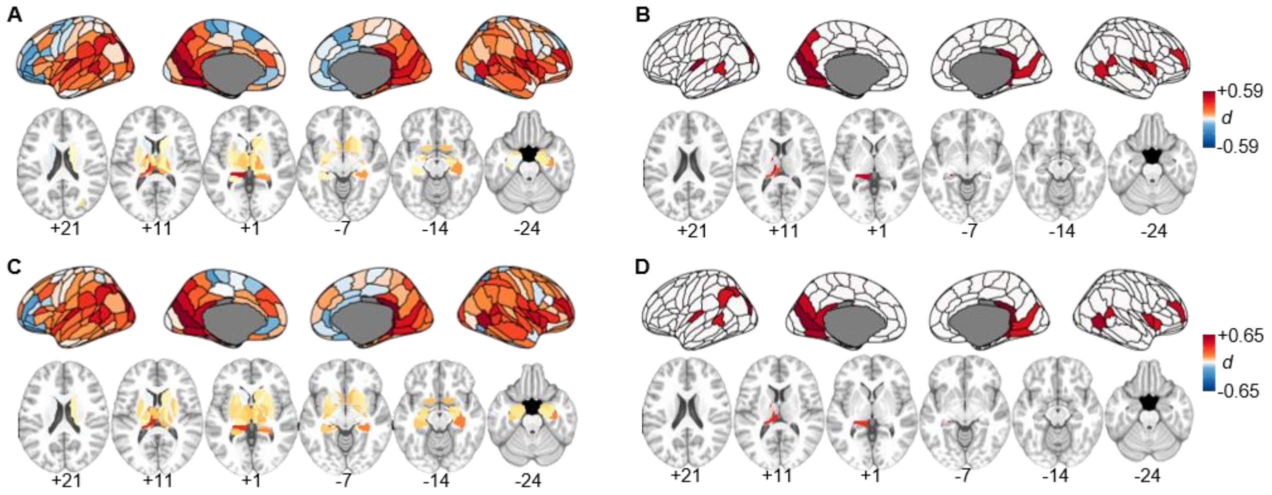


**eFigure 9. SC−FC coupling differences between adolescent MDD and HCs after sample matching.** (**A**) The differences of SC−FC coupling (measured as Cohen's *d*) between adolescent MDD (*n* = 168) and HCs (*n* = 101) at each brain region in the original cohort. (**B**) The SC−FC coupling of eighteen subregions show significant differences between adolescent MDD and HCs in the original cohort. (**C**) The differences of SC−FC coupling (measured as Cohen's *d*) between adolescent MDD (*n* = 101) and HCs (*n* = 101) at each brain region in the age−, gender− and BMI−matched sample. (D) The SC−FC coupling of twenty−two subregions show significant differences between adolescent MDD and HCs in the age−, gender− and BMI−matched sample.

© 2024 Xu M et al. *JAMA Network Open*

**eFigure 10.** Subgroup differences in SC−FC coupling at each brain region

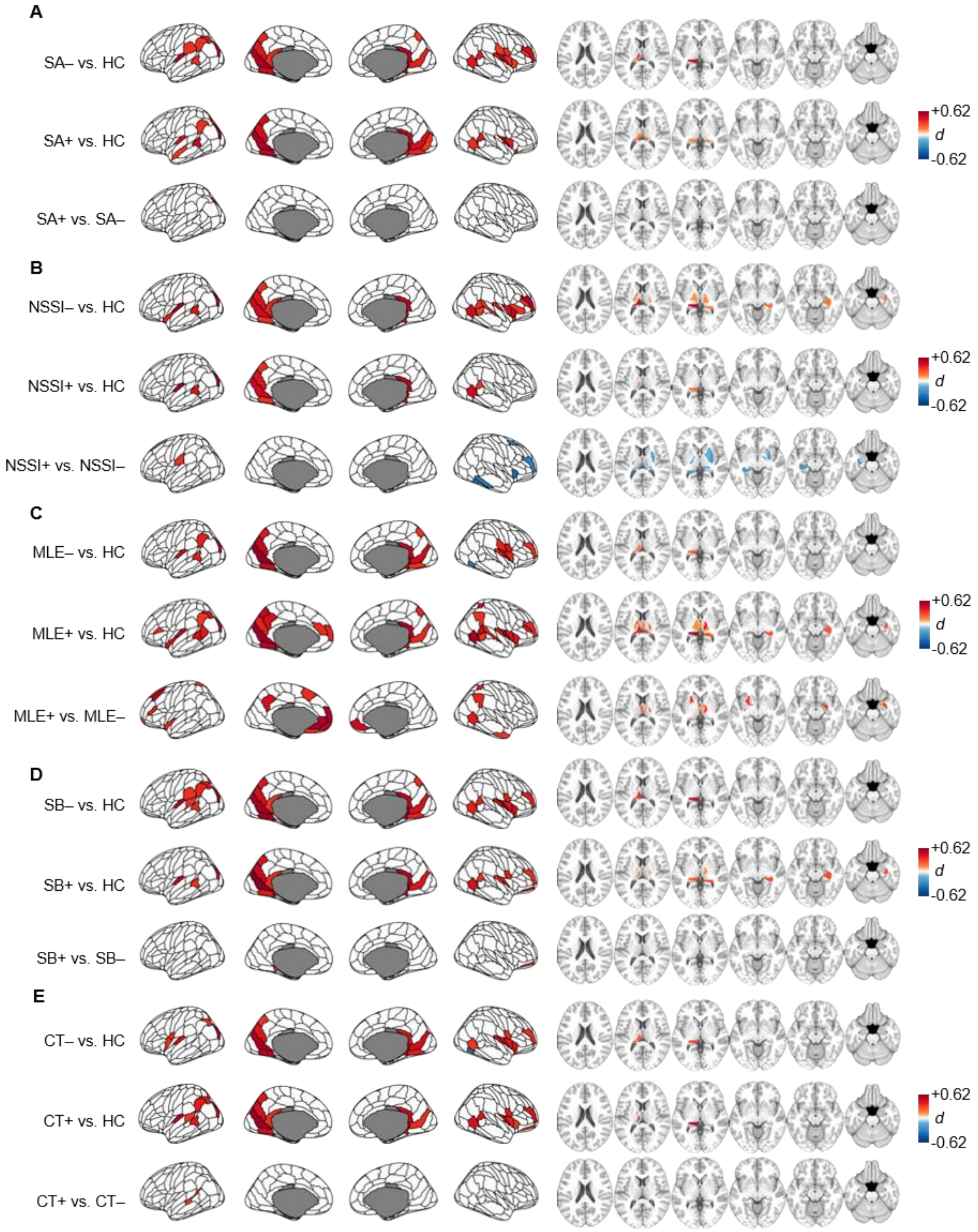


**eFigure 10. Subgroup differences in SC−FC coupling.** The Cohen's *d* is projected onto the brain, thresholding at $p < .05$. (**A**) Subgroup analysis of adolescent MDD with or without suicide attempt (SA+ and SA−). (**B**) Subgroup analysis of adolescent MDD with or without non−suicidal self−injury (NSSI+ and NSSI−). (**C**) Subgroup analysis of adolescent MDD with or without major life events (MLE+ and MLE−). (**D**) Subgroup analysis of adolescent MDD with or without

© 2024 Xu M et al. *JAMA Network Open*

school bullying (SB+ and SB−). (**E**) Subgroup analysis of adolescent MDD with or without childhood trauma (CT+ and CT−).

© 2024 Xu M et al. *JAMA Network Open*

**eFigure 11.** Regional SC−FC coupling differences among subgroups with different clinical characteristics

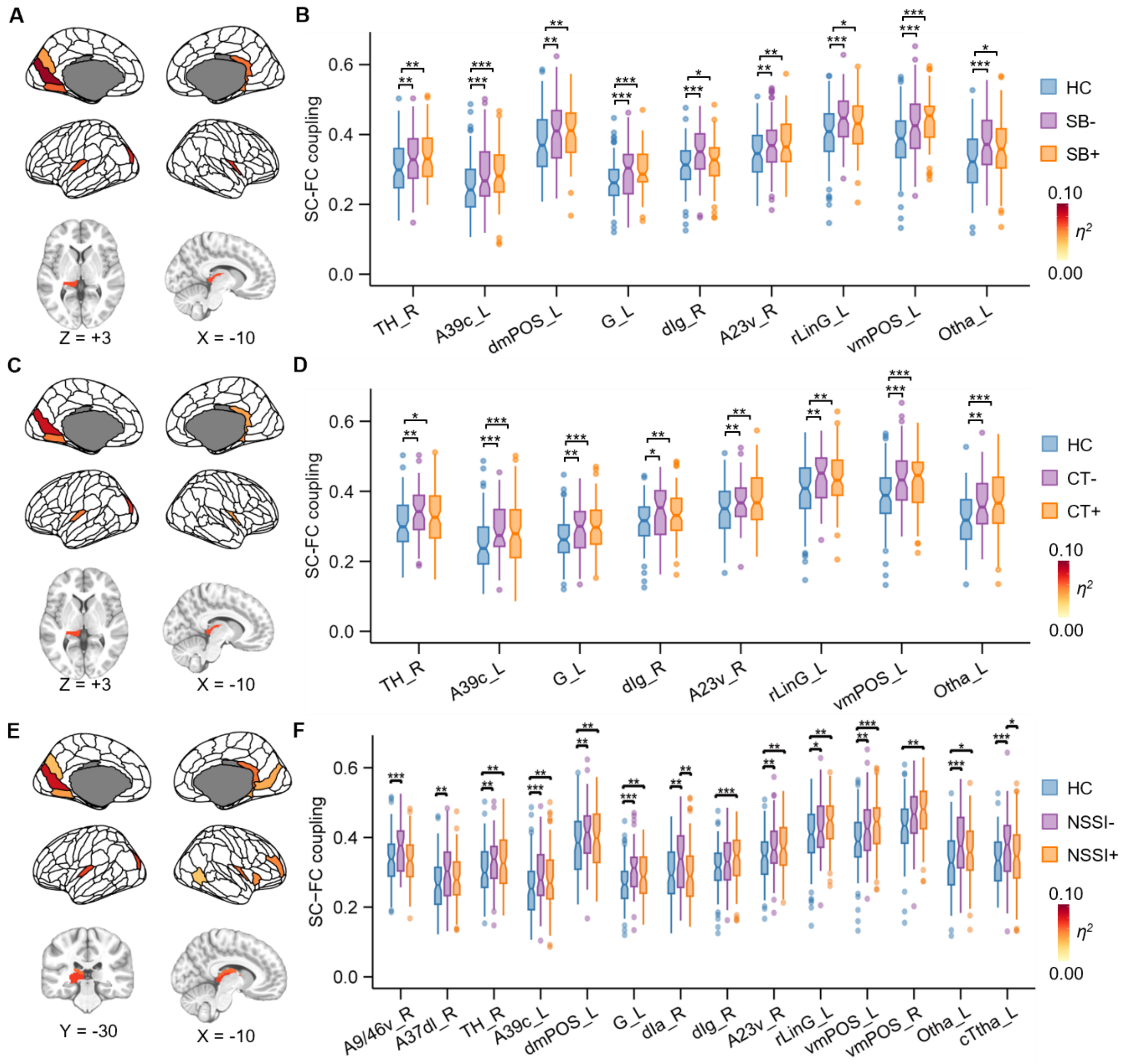


**eFigure 11. Regional SC−FC coupling differences among subgroups with different clinical characteristics.** In each boxplot, the "box" indicates interquartile range (IQR), the horizontal bar indicates the median value, and the whiskers include points that are within 1.5×IQR of upper and lower bounds of the IQR. (**A**) Regions with significant differences of SC−FC coupling among HCs, CT+ and CT−. Partial $\eta^2$ is mapped on the brain, thresholding at FDR−corrected $p < .05$. (**B**) *Post−hoc* comparisons of SC−FC coupling among HCs, CT+ and CT−. (**C**) Regions with significant differences of SC−FC coupling among HCs, SB+ and SB−. Partial $\eta^2$ is mapped on the brain, thresholding at FDR−corrected $p < .05$. (**D**) *Post−hoc* comparisons of SC−FC coupling among HCs, SB+ and SB−. (**E**) Regions with significant differences of SC-FC coupling among HCs, NSSI+ and NSSI−. (**F**) *Post-hoc* comparisons of SC-FC coupling among HCs, NSSI+ and NSSI−. Compared to HCs and NSSI+, NSSI− exhibited higher SC-FC coupling in subregions of right middle frontal gyrus (A9/46v), left thalamus (cTtha), right middle temporal gyrus (A37dl) and right insula (dIa).

© 2024 Xu M et al. *JAMA Network Open*

**eFigure 12.** Sensitivity analysis of the effects owing to the medication status on the main group comparison results

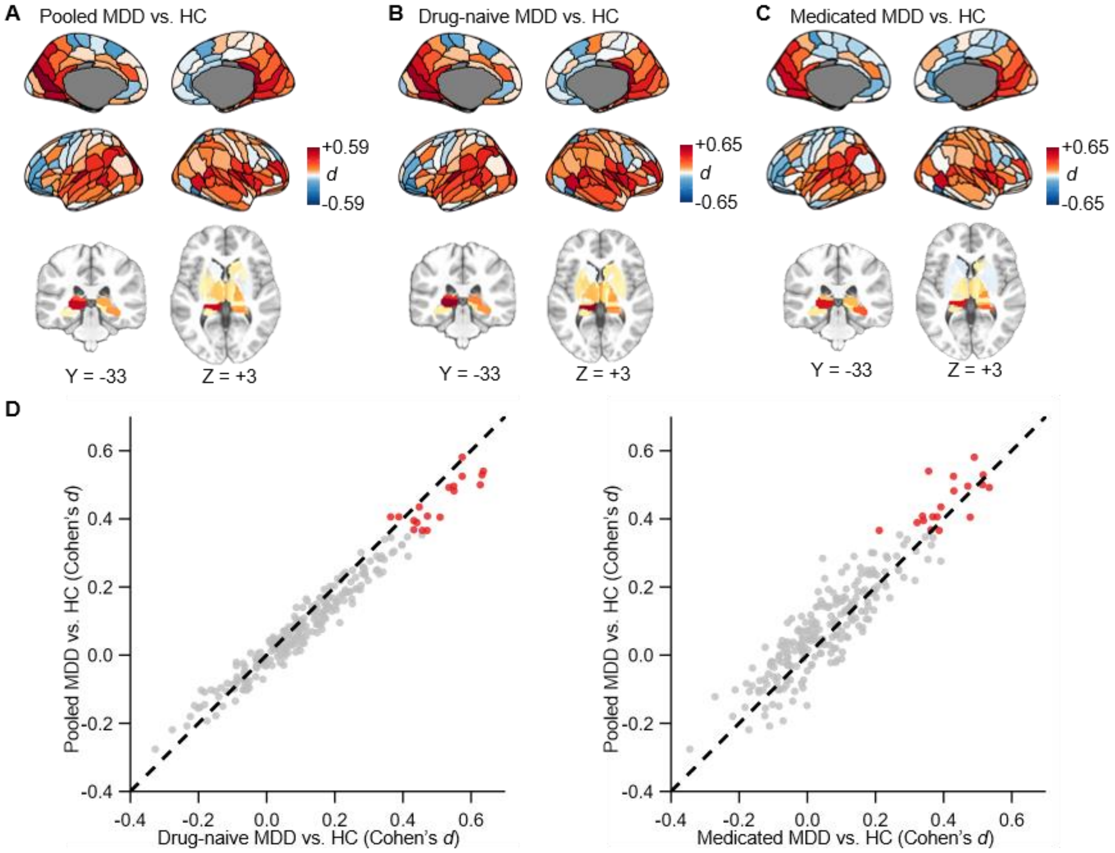


**eFigure 12. Sensitivity analysis of the effects owing to the medication status on the main group comparison results.** Confounders including age, sex, age$^2$, age × sex, age$^2$ × sex, BMI, total intracranial volume (ICV), and in−scanner head motion are regressed. (**A**) The differences of SC−FC coupling (measured as Cohen's *d*) between pooled adolescent MDD ($n$ = 168) and HCs ($n$ = 101). (**B**) The differences of SC−FC coupling (measured as Cohen's d) between drug−naïve adolescent MDD ($n$ = 98) and HCs ($n$ = 101). (**C**) The differences of SC−FC coupling (measured as Cohen's *d*) between medicated adolescent MDD ($n$ = 70) and HCs ($n$ = 101). (**D**) The SC−FC coupling alteration patterns are largely consensus regarding the medication−status.

© 2024 Xu M et al. *JAMA Network Open*

**eFigure 13.** Sensitivity analysis of the effects owing to the gender on the main group comparison results

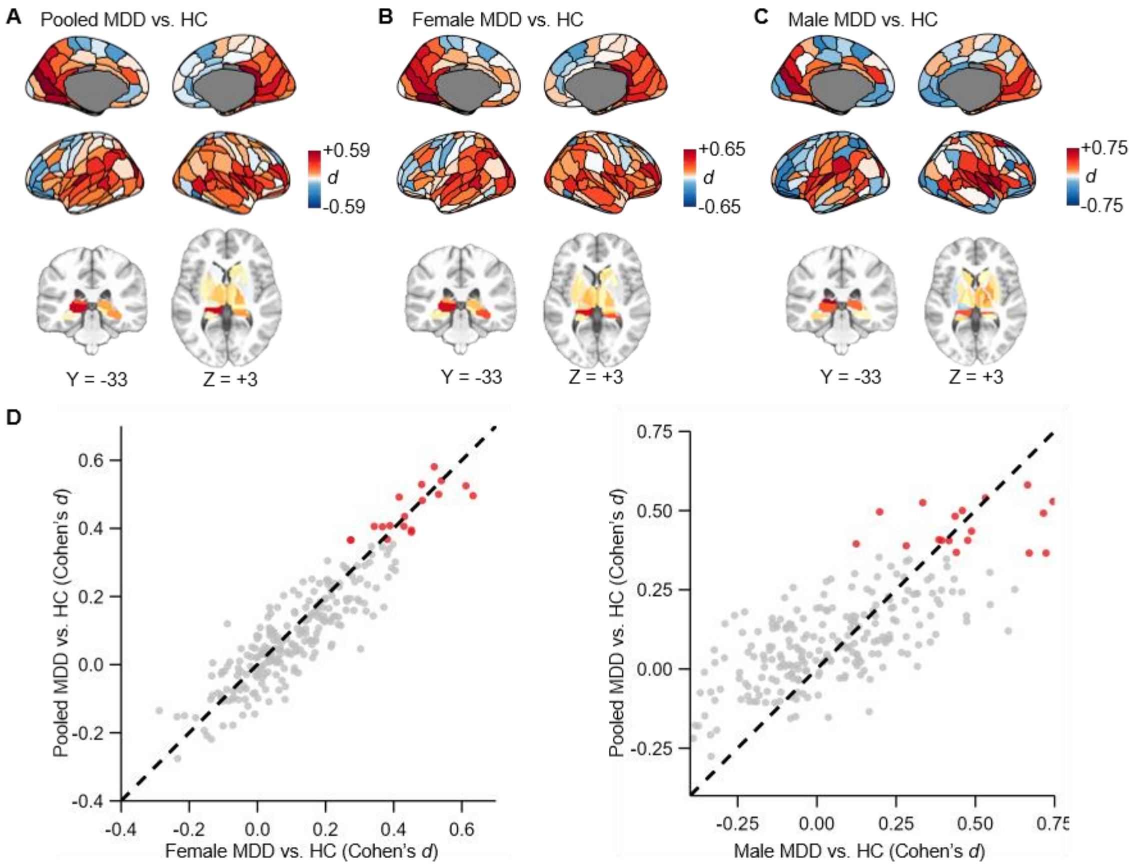


**eFigure 13. Sensitivity analysis of the effects owing to the gender on the main group comparison results.** Confounders including age, age$^2$, BMI, total intracranial volume (ICV), and in−scanner head motion are regressed. (**A**) The differences of SC−FC coupling (measured as Cohen's *d*) between pooled adolescent MDD (*n* = 168) and HCs (*n* = 101). (**B**) The differences of SC−FC coupling (measured as Cohen's *d*) between female adolescent MDD (*n* = 124) and female HCs (*n* = 61). (**C**) The differences of SC−FC coupling (measured as Cohen's *d*) between male adolescent MDD (*n* = 44) and male HCs (*n* = 40). (**D**) The SC−FC coupling alteration patterns are largely consensus across male and female subgroups.

© 2024 Xu M et al. *JAMA Network Open*

**eFigure 14.** Sensitivity analysis of the effects owing to other behavioral and environmental factors on the main group comparison results

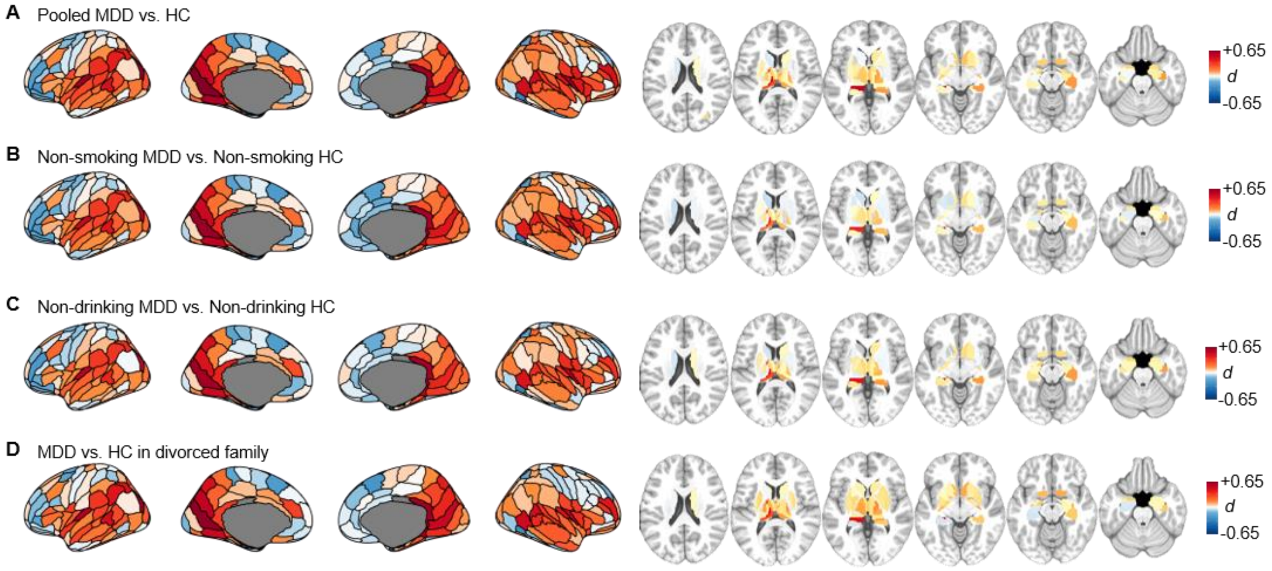


**eFigure 14. Sensitivity analysis of the effects owing to other behavioral and environmental factors on the main group comparison results.** Confounders including age, sex, age$^2$, age × sex, age$^2$ × sex, treatment history, BMI, total intracranial volume (ICV), and in−scanner head motion are regressed. (**A**) The differences of SC−FC coupling (measured as Cohen's *d*) between pooled adolescent MDD (*n* = 168) and HCs (*n* = 101). (**B**) The differences of SC−FC coupling (measured as Cohen's *d*) between non−smoking adolescent MDD (*n* = 151) and non−smoking HCs (*n* = 98). (**C**) The differences of SC−FC coupling (measured as Cohen's *d*) between non−drinking adolescent MDD (*n* = 148) and non−drinking HCs (*n* = 91). (**D**) The differences of SC−FC coupling (measured as Cohen's *d*) between adolescent MDD (*n* = 137) and HCs (*n* = 96) in non−divorced family.

© 2024 Xu M et al. *JAMA Network Open*

**eFigure 15.** Distributions of the SC−FC coupling calculated by different SC−related matrices

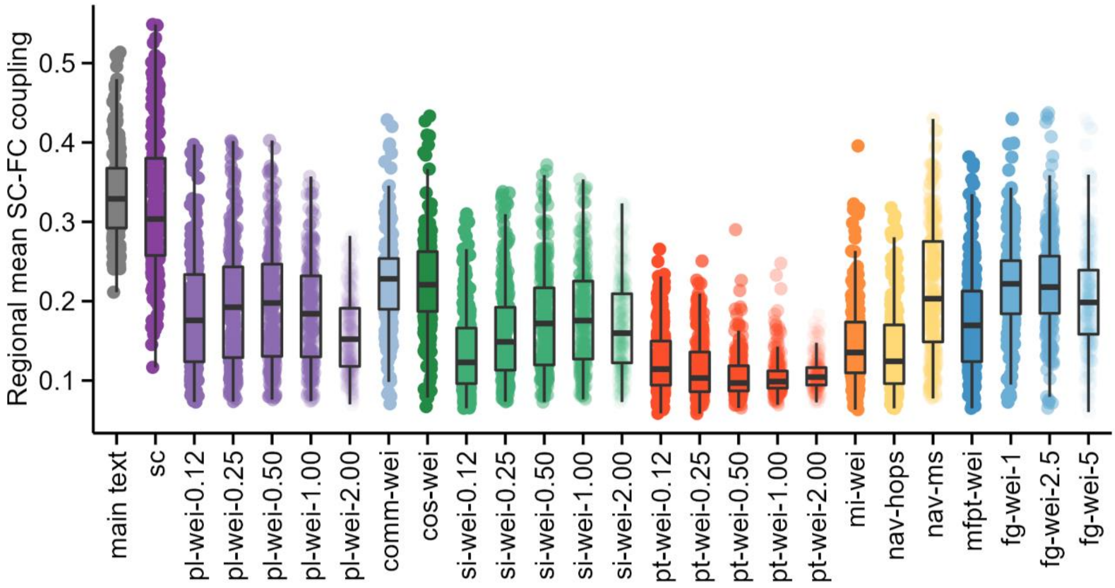


**eFigure 15. Distributions of the SC−FC coupling calculated by different SC−related matrices.** Each point represents the mean regional SC−FC coupling strength of a brain region across healthy participants. Compared with single predictor models, the SC−FC coupling strengths calculated in the main text were higher. For details about SC-related matrices, please refer to eMethods.

**eFigure 16.** Distributions of the SC−FC coupling calculated by selected combinations of SC−related matrices

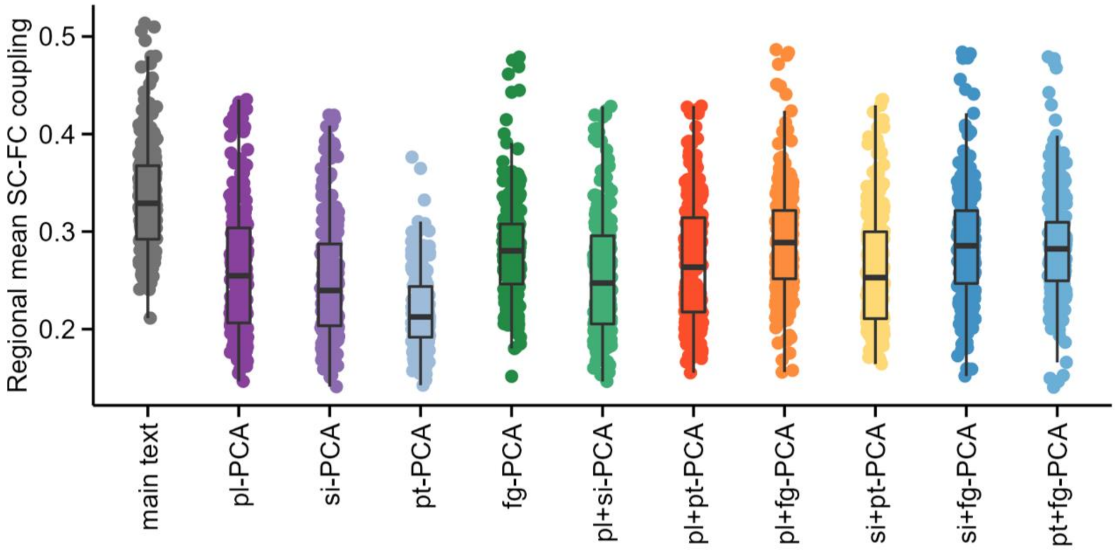


**eFigure 16. Distributions of the SC−FC coupling calculated by selected combinations of SC−related matrices.** In order to validate whether a subset of predictors could achieve comparable SC−FC coupling strength, we repeatedly calculated the SC−FC coupling strength based on ten selected combinations of predictors. Similarly, PCA was applied to generate three orthonormal bases which could collectively explain more than 80% of variance for all selected combinations. As the figure shown, the SC−FC coupling strengths calculated in the main text which utilized all predictors were higher than selected subsets of predictors. Each point represents the mean regional SC−FC coupling strength of a brain region across healthy participants. pl−PCA, PCA was performed on all path length−related matrices; si−PCA, PCA was performed on all search information−related matrices; pt−PCA, PCA was performed on all path transitivity−related matrices; fg−PCA, PCA was performed on all flow graph−related matrices; pl+si−PCA, PCA was performed on all path length−related matrices and search information−related matrices; pl+pt−PCA, PCA was performed on path length−related matrices and path transitivity−related matrices; si+pt−PCA, PCA was performed on search information−related matrices and path transitivity−related matrices; si+fg−PCA, PCA was performed on search information−related matrices flow grapth−related matrices; pt+fg−PCA, PCA was performed on path transitivity−related matrices and flow graph−related matrices.

**eFigure 17.** Correlations between the group differences of SC−FC coupling calculated based on all SC−related matrices and on single matrix

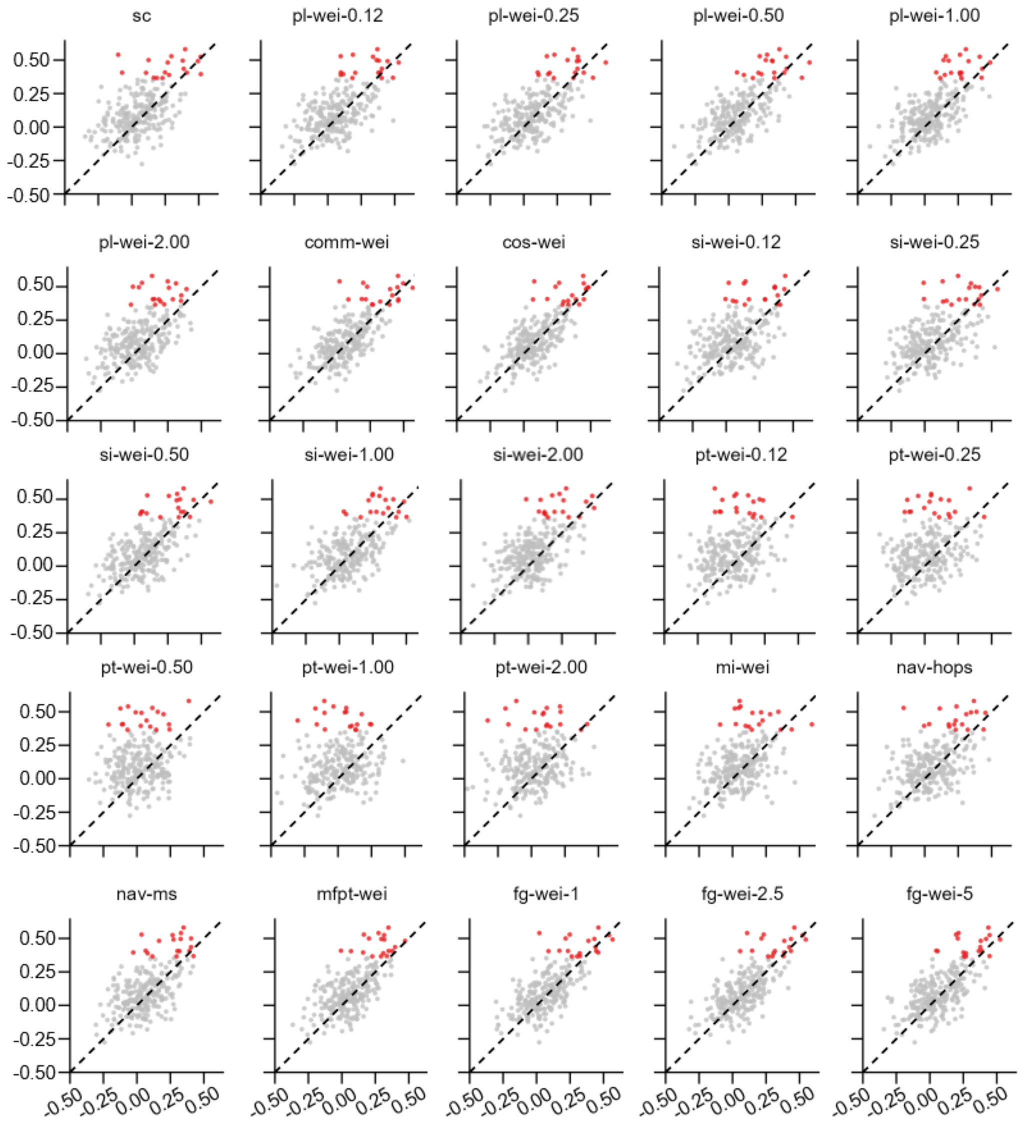


**eFigure 17. Correlations between the group effect sizes of SC−FC coupling calculated based on all SC−related matrices and on single matrix.** The red dots represent those abnormal subregions found in the main text. The distributions of the group differences of SC−FC coupling based on single predictor are largely in accordance with the results reported in the main text. Additionally, the SC−FC coupling calculated based on all predictors could achieve relatively higher effect sizes (the red dots tend to cluster at the right upside corner and above the diagonal line). For details about SC-related matrices, please refer to eMethods.

© 2024 Xu M et al. *JAMA Network Open*

**eFigure 18.** Correlations between the group effect sizes of SC−FC coupling calculated based on all SC−related matrices and on selected subsets of matrices

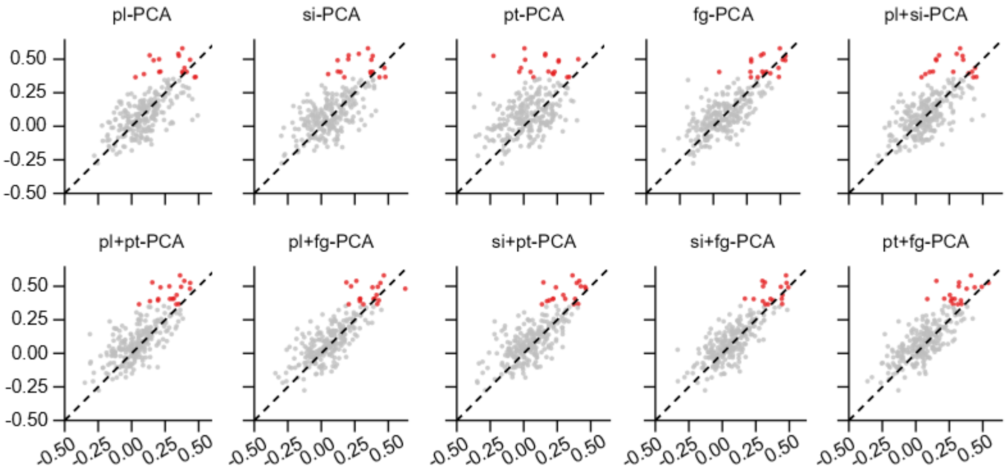


**eFigure 18. Correlations between the group effect sizes of SC−FC coupling calculated based on all SC−related matrices and on selected subsets of matrices.** The red dots represent those abnormal subregions found in the main text. The distributions of the group differences of SC−FC coupling based on selected subsets of predictors are largely in accordance with the results reported in the main text. Additionally, the SC−FC coupling calculated based on all predictors could achieve relatively higher effect sizes (the red dots tend to cluster at the right upside corner and above the diagonal line). pl−PCA, PCA was performed on all path length−related matrices; si−PCA, PCA was performed on all search information−related matrices; pt−PCA, PCA was performed on all path transitivity−related matrices; fg−PCA, PCA was performed on all flow graph−related matrices; pl+si−PCA, PCA was performed on all path length−related matrices and search information−related matrices; pl+pt−PCA, PCA was performed on path length−related matrices and path transitivity−related matrices; si+pt−PCA, PCA was performed on search information−related matrices and path transitivity−related matrices; si+fg−PCA, PCA was performed on search information−related matrices flow grapth−related matrices; pt+fg−PCA, PCA was performed on path transitivity−related matrices and flow graph−related matrices.

**eFigure 19.** The effect of different covariate sets on the group-differed SC-FC coupling patterns

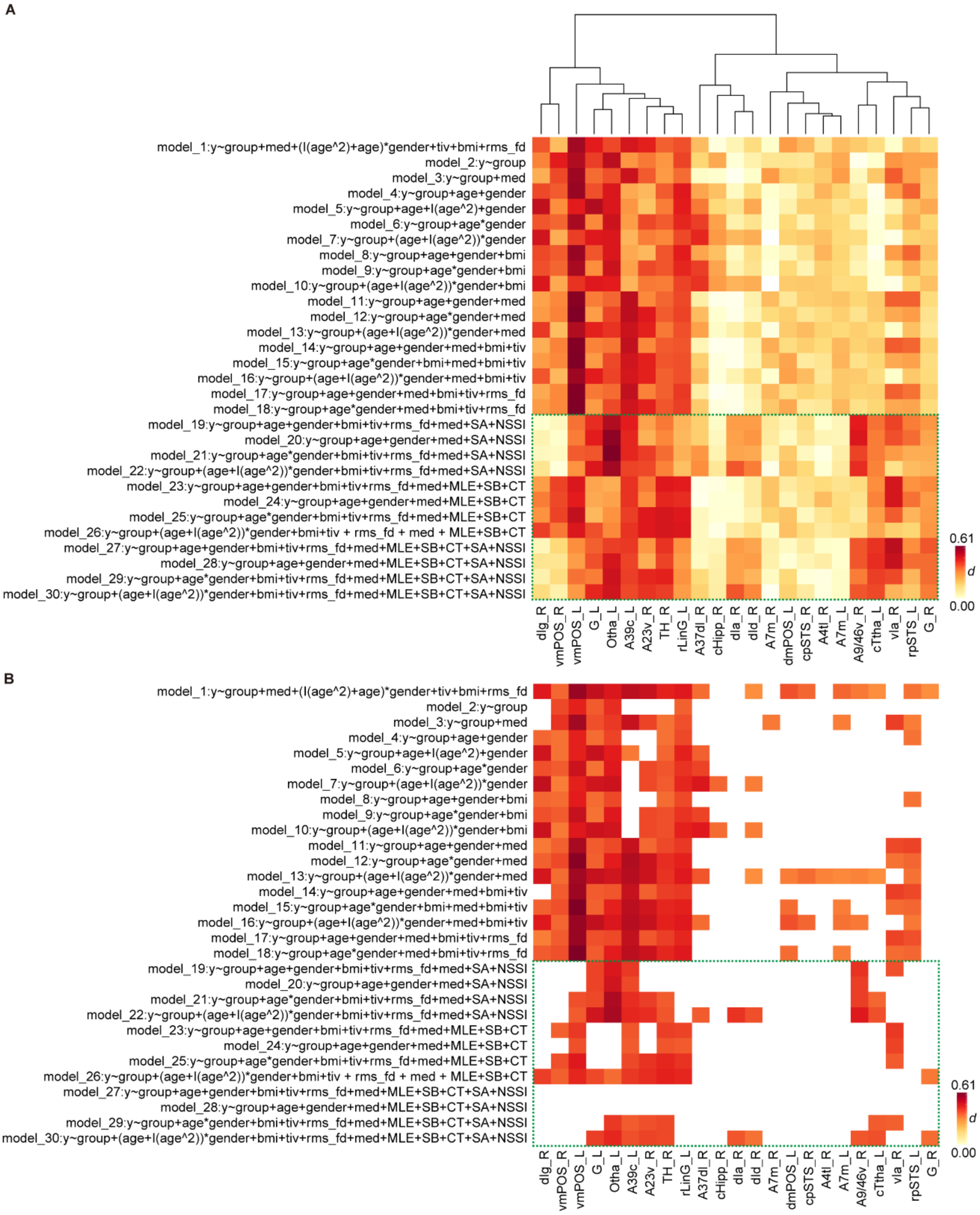


**eFigure 19. The effect of different covariate sets on the group-differed SC-FC coupling patterns.** Covariates sets were constructed by different combinations of demographic variables (*i.e.*, age, gender), procedural factors (*i.e.*, in-scanner head motion), anatomical variables (*i.e.*, BMI, ICV), clinical variable (*i.e.*, treatment history), and environmental/behavioral variables (*i.e.*, childhood trauma, suicide attempt, *etc.*). The green bounding box indicated the covariate sets including environmental/behavioral factos. (**A**) The effect sizes (Cohen's *d*) of brain regions with significant group differences (FDR corrected) obtained by different linear regression models. Only regions with significant group differences (FDR corrected) in least one model were presented. (**B**) The effect sizes (Cohen's *d*) of

© 2024 Xu M et al. *JAMA Network Open*

brain regions with significant group differences (FDR corrected) in each linear regression model. Overall, SC-FC coupling increases in default mode network, insula and visual network in adolescent depression could be robustly identified in most models; and inclusions of environmental/behavioral variables will cancel some regions with significant group differences identified by other models.

© 2024 Xu M et al. *JAMA Network Open*

## eMethods

### Assessment of childhood trauma

The Childhood Trauma Questionnaire (CTQ) is a 28-item assessment used to measure a history of childhood trauma. It includes five subscales that assess different types of traumas, such as emotional abuse, physical abuse, sexual abuse, emotional neglect, and physical neglect. Individuals with 1 or more subscales exceeded recommended cutoffs[2-4] were considered to have childhood trauma (cutoff scores: emotional abuse, 13; physical abuse, 10; sexual abuse, 8; emotional neglect, 15; physical neglect, 10).

### Assessment of suicide attempt

The assessment of suicide attempt was based on the following six questions[5]:

1. *Have you wished you were dead or wished you could go to sleep and not wake up?*
2. *Have you had any actual thoughts of killing yourself?*
3. *Have you been thinking about how you might do this?*
4. *Have you had these thoughts and had some intention of acting on them?*
5. *Have you started to work out or worked out the details of how you would kill yourself? Do you intend to carry out this plan?*
6. *Have you ever done anything, started to do anything, or prepared to do anything to end your life?*

If the answers to questions 1 and 2 were both negative, the participants were considered to have no suicidal ideation. Otherwise, we continued to ask questions 3-6. If the participants have already taken some actions towards killing themselves (*i.e.*, the answer to question 6 was positive), they were considered to have suicide attempt. Otherwise, the participants were considered to only have suicidal ideation but without suicide attempt.

### Assessment of non-suicidal self-injurious behavior

To assess non-suicidal self-injurious behavior, we asked participants if they had ever had an instance of directly and intentionally hurting themselves, such as scratching/cutting/pinching/biting/hitting themselves, pulling hair, *etc*. Participants who answered “No” were categorized as having no self-injurious behaviors. For those who answered “Yes”, we further inquired for their intention of the self-injurious behavior. Participants who confirmed engaging in self-injury without any intention to taking their own life (non-suicidal intention like stress relief, emotional improvement and seeking sympathy, *etc.*) were considered to have non-suicidal self-injurious behavior.

### Assessment of major life events and school bullying

For assessment of major life events, we asked participants whether they had experienced a major stressful life event or not[6], such as life-threatening illnesses, the death or illness of a close friend or family member, parental divorce, financial difficulties, car accidents, and conflicts with romantic partners, *etc*. Participants who gave a confirmed answer were considered to have experienced major life events. Similarly for school bullying, we asked participants if they had experienced school bullying, such as verbal abuse, verbal threats, bodily injury, property damage, and sexual violence, or no school bullying experiences. Those who answered “Yes” were considered to have exposed to school bullying.

### Image acquisition

Multimodal brain images were acquired using a Siemens Magnetom Skyra 3T scanner with a 32−channel head coil. Each participant was instructed to lie down and relax, to keep their eye closed and to stay awake and to avoid performing

© 2024 Xu M et al. *JAMA Network Open*

specific cognitive task. The high resolution T1-weighted (T1w) structural images were acquired using magnetization−prepared rapid gradient−echo (MPRAGE) sequence with the following parameters: repetition time (TR) = 2000 ms, echo time (TE) = 2.56 ms, inversion time (TI) = 900 ms, flip angle = 9°, matrix size = 256 × 256, field of view (FOV) = 256 mm × 256 mm, slice thickness = 1 mm, slices per slab = 192, and voxel size was 1.0 mm × 1.0 mm × 1.0 mm. The resting−state fMRI was acquired using the gradient echo EPI sequence with following parameters: TR = 2000 ms, TE = 30 ms, flip angle = 90°, FOV = 220 mm× 220 mm, slice number = 36, and slice thickness = 3.0 mm. Totally 240 EPI volumes were acquired. The reconstructed voxel size was 3.4 mm × 3.4 mm × 3.0 mm, and the layer thickness was 3.0 mm. The parameters of diffusion−weighted MRI (DWI) were as follows: TR = 7000 ms, TE = 89.0 ms, acquisition matrix = 128 × 128, FOV = 256 mm × 256 mm, slice thickness = 3.0 mm, number of slices = 50, voxel resolution = 2.0 mm × 2.0 mm × 3.0 mm, flip angle = 90°, number of diffusion gradient directions = 64, b = 0 and 1000 s/mm2, number of excitations = 1.

## Multimodal imaging data preprocessing

All available functional images and diffusion tensor images (DTI) were preprocessed using fMRIPrep 20.2.5 (RRID: SCR_016216)[7] and QSIPrep 0.14.3 (RRID: SCR_002502)[8], respectively. Both tools are based on Nipype 1.6.1 (RRID: SCR_002502)[9]. The following descriptions of fMRIPrep's and QSIPrep's preprocessing workflows are both based on boilerplate distributed with the software covered by 'no rights reserved' (CC0) license. Internal operations of fMRIPrep use Nilearn 0.6.2 [RRID: SCR_001362][10], ANTs 2.3.3 (RRID: SCR_004757), FSL 5.0.9 (RRID: SCR_002823), AFNI 20160207 (RRID: SCR_005927), and FreeSurfer 6.0.1 (RRID: SCR_001847). Internal operations of QSIPrep use Nilearn 0.8.1, ANTs 2.3.1, FSL 6.0.3, Dipy 1.3.0 (RRID: SCR_000029)[11], MRtrix 3.0 (RRID: SCR_006971)[12]. For more details of these two pipelines, please refer to the section corresponding to workflows in fMRIPrep's and QSIPrep's documentations.

**Anatomical T1-weighted MRI preprocessing**

T1w images were corrected for intensity non-uniformity (INU) using *N4BiasFieldCorrection*[13] distributed with ANTs and were used as T1-references. The T1w-reference was then skull-stripped with a Nipype implementation of the *antsBrainExtraction.sh* workflow, using the OASIS30ANTs as the target template. Brain tissue segmentation of cerebrospinal fluid (CSF), white-matter (WM) and gray-matter (GM) was performed on the brain-extracted T1w using *fast*[14]. Volume-based spatial normalization to the ICBM 152 Nonlinear Asymmetrical template version 2009c (MNI152NLin2009cAsym, RRID:SCR_008796)[15] was performed through nonlinear registration with *antsRegistration*[16], using brain-extracted versions of both T1w volume and the T1w template.

**Resting state fMRI preprocessing**

Functional images were motion corrected with respect to a BOLD reference volume (which had firstly been generated via a custom methodology of fMRIPrep) using FSL's *mcflirt*[17] and slice-time corrected using AFNI's *3dTshift*[18]. A deformation field to correct for susceptibility distortions was estimated using fMRIPrep's fieldmap-less approach[19,20]. Based on the estimated susceptibility distortion, a corrected EPI (echo-planar imaging) reference was calculated for a more accurate co-registration with the anatomical reference. The BOLD reference was then co-registered to the T1w image using FSL's *flirt*[21] with the boundary-based registration[22] cost-function. Co-registration was configured with nine degrees of freedom to account for distortions remaining in the BOLD reference. Motion correcting transformations, susceptibility-distortion-correcting warp, BOLD-to-T1w transformation and T1w-to-template (MNI152NLin2009cAsym) warp were concatenated and applied in a single step using *antsApplyTransforms* with Lanczos interpolation[23]. Several confounding time-series were calculated based on the preprocessed BOLD: framewise

© 2024 Xu M et al. *JAMA Network Open*

displacement (FD), DVARS and three region-wise global signals. FD and DVARS were calculated for each functional run, both using their implementations in Nipype[24]. The three global signals were extracted within the CSF, the WM, and the whole-brain masks. The first ten volumes were discarded for signal stabilization.

**DTI preprocessing**

All DTI images were denoised with Marchenko-Pastur principal components analysis (MP-PCA) by MRtrix3's *dwidenoise*[25]. The B1 field inhomogeneity was then corrected using *dwibiascorrect* from MRtrix3 with the *N4* algorithm[13]. After B1 bias correction, the mean intensity of the DWI series was adjusted so all the mean intensity of the $b$ = 0 images matched across each separate DWI scanning sequence. Head motion and eddy currents were further corrected using FSL's *eddy* with outlier replacement[26]. The $b$ = 0 image from the preprocessed DWI data was registered to a skull-stripped, anterior commissure-posterior commissure (AC-PC) aligned T1w anatomical image. Finally, the DWI time-series were resampled into the AC-PC space with 2 mm isotropic voxels.

## Diffusion model fitting

A ball–and–sticks diffusion model was fitted to each subject's resampled preprocessed DTI data using FSL's *bedpostx*, which runs Markov Chain Monte Carlo sampling to build up distributions on diffusion parameters at each voxel, and enables to model crossing fibers within each voxels[27]. After model fitting, probabilistic tractography was performed using FSL's *probtrackx2_gpu*, which respectively samples voxel-wise fiber orientation and diffusion parameter distributions to model anatomical trajectories between specified seed and target regions[27].

## Seed and target regions for tractography

Seed and target regions were defined in native DTI space after co-registering the Brainnetome atlas to the resampled preprocessed $b$ = 0 reference DWI image. The pipeline of defining the seed and target regions for probabilistic tractography was presented in **eFigure 2**. First, the preprocessed T1w anatomical images, brain tissue masks (GM, WM, CSF) and the brain atlas were all registered to the native DTI (AC-PC) space. Specifically, the preprocessed T1w images were registered to the resampled preprocessed DWI reference image ($b$ = 0) through FSL's *flirt*, resulting a transformation matrix, *i.e.*, T1w2dwi.mat (**eFigure 2A**). Then, GM mask, WM mask, CSF mask were registered to native DWI space through FSL's *flirt* with T1w2dwi.mat as initialized transformation matrix, and the registered images were binarized at 0.5 to get the registered masks (**eFigure 2B**).

Second, the brain atlas was registered to the native DWI space via a two-step manner (**eFigure 2C**). Specifically, the brain atlas (in MNI space) was registered to native T1w space through *antsApplyTransforms*. Notably, the transforms (*i.e.*, MNI_to_t1w_xfm.h5) were automatically generated by the anatomical preprocessing workflow of fMRIprep. Then, FSL's *flirt* with initialized transformation matrix (T1w2dwi.mat) was applied to register the atlas generated from the former step to native DWI space. In both steps, the nearest neighborhood interpolation method was used.

Finally, the seed masks were defined in the native DTI space (**eFigure 2D**). For cortical regions, seed mask only included voxels in the ribbon along the gray-white boundary. For subcortical regions, seed mask included all voxels in each brain region defined by atlas. The gray-white boundary was defined by using *fslmaths –edge* function on the white matter segmentation mask in native DWI space, and the ribbon image was generated by dilating the gray-white matter boundary image by 2 mm. **eFigure 2H** illustrates the seed voxels of each brain regions defined by the Brainnetome atlas.

Each region was selected as a seed region and its connectivity strength to other 245 regions was calculated using probabilistic tractography. For each seed region, 2000×$n$ streamlines were sampled, where $n$ is the number of voxels in that region. Specifically, for cortical regions, only voxels in a ribbon along the gray-whiter boundary (**eFigure 2D**) were

© 2024 Xu M et al. *JAMA Network Open*

selected as seed voxels, while all voxels in each subcortical region were selected as seed voxels due to its much smaller structure compared to cortical structures. For each streamline, the tracking parameters were as follows: step length = 0.5 mm, maximum steps = 2000, curvature threshold = 0.2 (*i.e.*, approximately correspond to 10° for maximum turning angle). Loop check was also enabled to avoid a streamline looped back itself. To increase the biological plausibility of white matter pathways reconstructed with probabilistic tractography, WM mask and CSF mask generated from fMRIprep's structural preprocessing workflow were also registered to native DWI spaced and were defined as waypoint mask and exclusion mask respectively, *i.e.*, only streamlines that passed through the WM mask would be considered valid and would be discarded if they entered cerebral-spinal fluid (**eFigure 2E-F**). Additionally, a superficial gray mask was generated by subtracting the seed mask from the GM mask and was defined as termination mask, *i.e.*, streamlines would be terminated as soon they entered superficial gray matter (**eFigure 2F**). This tractography process yielded a 246×246 weighted matrix, where each entry corresponded to the count of streamlines that passing through the seed and target region.

## Image quality control

Data were assessed using visual reports generated from fMRIprep and QSIprep, and were also visually inspected for whole–brain field of view coverage. 3 participants were excluded due to failed preprocessing of DWI image. 4 participants were excluded due to incomplete MRI volume coverage or because of inadequate signal in several brain regions. Functional data with more than 25% of the frames exceeded 0.2 mm FD were considered to be contaminated by excessive head motion, resulting exclusions of 21 participants. Another 10 participants were excluded due to incomplete data collection, *e.g.*, lack of functional data or diffusion data.

## SC-based communication models

As mentioned in the main text, the sparse structural connectivity matrix was transformed into 34 fully-weighted matrices based on a suite of communication strategies incorporating both centralized and decentralized processes, topological similarities, and spatial embeddings[28]. Each entry in these matrices described a putative functional linkage strength between the source and target regions. These 34 matrices included six path length matrices parameterized at different weight-to-cost transformations, two communicability matrices[29], two cosine similarity matrices, six search information matrices parameterized at different weight-to-cost transformations[1], six path transitivity matrices parameterized at different weight-to-cost transformations[1], two matching index matrices[30], two navigation matrices[31], two mean first passage time matrices[32], and six flow graphs parameterized at different time scales[33,34]. Additionally, Euclidean distance between two brain regions was also included as a predictor for functional connectivity. The followings presented the definition and calculation process of these matrices.

**Path length.** For weighted structural connectivity, the edge weight can be transformed to a cost. The path length $l_{ij}$ can then be defined as the minimized total travel cost between brain region *i* and *j*. For binarized structural connectivity, the cost metric is identical to edge weight. In present study, the negative power of an edge weight $w_{ij}$ was used for cost metric, *i.e.*, $c_{ij} = w_{ij}{}^{-\gamma}$, where $\gamma = 0.12, 0.25, 0.50, 1.00, 2.00$. Together, these matrices were denoted as *pl-bin* and *pl-wei-* followed by $\gamma$ values.

**Communicability.** Communicability between a pair of brain regions is the weighted sum of the length of all possible walks[29]. For weighted structural connectivity, the communicability matrix is calculated as $C_{wei} = e^{A'}$, where $A'$ is the normalized connectivity matrix, *i.e.*, $A' = D^{-1/2}AD^{1/2}$. For binarized network, the communicability is directly calculated as $C_{wei} = e^{A}$. These matrices were denoted as *comm-bin* and *comm-wei* in present study.

**Cosine similarity.** The cosine similarity is defined as the angle between two vectors, *i.e.*, $S_{ij} = w_i w_j / (\|w_i\| \|w_j\|)$. In present study, $w_i$ and $w_j$ corresponded to the structural connectivity profiles (the row of structural connectivity) of regions *i* and *j*. These metrices were denoted as *cos-wei* and *cos-bin* for weighted and binarized structural connectivity respectively.

**Search information.** Search information quantifies the information (in bits) required to access the shortest path linking a pair of brain regions[1]. For a given shortest path between brain region *s* and *t*, $\Omega_{s\to t} = \{s, i, j, \ldots, k, t\}$ denotes the region sequence along the path, and $\pi_{s\to t} = \{w_{si}, w_{ij}, \ldots, w_{kt}\}$ is the edge weight sequence along the path, the possibility of taking this path can be calculated as $p(\pi_{s\to t}) = p_{s\to i} \times p_{i\to j} \times \ldots \times p_{k\to t}$, where $p_{i\to j} = \frac{A_{ij}}{\sum_j A_{ij}}$. Then, the search information $SI_{st}$ between region *s* and *t* can then be calculated as $-\log_2 p(\pi_{s\to t})$. In present study, search information matrices were calculated based on path length matrices and were denoted as *si-bin* and *si-wei-* followed by corresponding $\gamma$ values.

**Matching index.** Matching index quantifies the similarity between a pair of brain regions based on the overlap of their adjacent regions (excluding themselves) [1,30]. The adjacent region set $N_i$ includes those regions with direct connections to region *i*, *i.e.*, $N_i = \{j | A_{ij} > 0\}$. The matching index can then be obtained by $MI_{ij} = \frac{|(N_i - \{j\}) \cap (N_j - \{i\})|}{|(N_i - \{j\}) \cup (N_j - \{i\})|}$. In present study, we denoted these matrices as *mi-bin* and *mi-wei* for binarized and weighted structural connectivity respectively.

**Path transitivity.** Path transitivity between the source region *s* and target region *t* measures the density of local detours available along the shortest path[1], and is calculated as $PT_{\pi(s\to t)} = \frac{2\sum_{i\in\Omega}\sum_{j\in\Omega} MI_{ij}}{|\Omega|(|\Omega|-1)}$. In present study, as path transitivity matrices were calculated based on path length matrices, these matrices were denoted as *pt-bin* and *pt-wei-* followed by corresponding γ values.

**Navigation.** Navigation describes an information routing strategy atop the network of anatomical pathways, *i.e.*, neural information always propagates from one region to another nearest region in some metric space. In present study, we calculated two matrices, *nav-ms* and *nav-hops*, based on navigation process atop the structural connectivity in Euclidean space. *nav-ms* corresponded to the total navigation length between each pair of brain regions, while *nav-hops* corresponded to the total number of hops between each pair of brain regions. Notably, navigation process doesn't guarantee a complete path from source region to target region. For incomplete navigation paths, the entries were filled with ∞.

**Mean first passage time.** Mean first passage time is defined as the expectation distance of a random walker travels from a source region to a target region. In present study, we denoted the mean first passage time as *mfpt-wei* and *mfpt-bin* for weighted and binarized structural connectivity matrix respectively.

**Flow graphs.** A flow graph[34] transforms an adjacency matrix of a network, $A_{ij}$, into a fully weighted adjacency matrix, $A(t)'_{ij}$, where each entry corresponds to the probabilistic flow of random walkers between regions *i* and *j* at time *t*. For a continuous-time random walkers with dynamics $\dot{p_i} = \sum_j L_{ij} p_j$, the corresponding flow graph is given by $A(t)'_{ij} = (e^{-tL})_{ij} s_j$, where the Laplacian operator $L_{ij} = \delta_{ij} - A_{ij}/s_j$, $s_j = \sum_i A_{ij}$. In present study, flow graphs were calculated based on both binarized (thresholding at 0) and weighted structural connectivity at different time scales, *i.e.*, *t* = 1, 2.5, 5. These matrices were denoted as *fg-bin-* and *fg-wei-* followed by parameter *t*.

**Euclidean distance.** In present study, Euclidean distance was calculated based on the centroid coordinates of brain regions defined by an atlas and was denoted as *distance*.

© 2024 Xu M et al. *JAMA Network Open*

## Matching procedure

In order to create a sample with balanced age, gender and BMI between healthy and depressive group, we used *R* package *MatchIt* [35] (*R* version 4.3.1, package version 4.5.4) to perform subset selection, which implements a wide range of matching methods for improving parametric statistical models. We used default parameters for the matching procedure, *i.e.*, a nearest neighbor matching on the propensity score estimated using logistic regression. The matching was based on age, gender and BMI, and ratio of healthy and depression subjects was 1:1.

## Enrichment analysis via spin-based permutation testing

To evaluate whether the distribution of associations tended to be concentrated within specific brain cortical networks and lobar structure, enrichment analysis was conducted via spin−based permutation testing. The spin-based permutation test is a conservative statistical method with accounting for the different size of each network (lobe) and the spatial autocorrelation of brain structures (www.github.com/frantisekvasa/rotate_parcellation) [36,37]. Specifically, the proportion of brain cortical regions that survived the $p$ thresholding ($p < .05$, uncorrected) of association analysis was calculated as the test statistics; the cortical statistical maps from association analysis were projected onto a sphere, which was rotated 5000 times to create a null distribution of the test statistics[38]. Networks (or lobes) were considered to have significant enrichment if the empirical test statistic was in the top 5% of the null distribution, *i.e.*, $p_{spin} < .05$.

© 2024 Xu M et al. *JAMA Network Open*

**eTable 1.** The regions that defined in Brainnetome atlas at different levels

| Lobe | Gyrus (Macro-anatomy level) | Left and Right Hemisphere (Subregion level) | Label ID.L | Label ID.R | Anatomical and modified Cyto-architectonic descriptions (Subregion level) | lh.MNI(X,Y,Z) | rh.MNI(X,Y,Z) |
|---|---|---|---|---|---|---|---|
| Frontal Lobe | SFG, Superior Frontal Gyrus | SFG_L(R)_7_1 | 1 | 2 | *A8m, medial area 8* | −5 ,15, 54 | 7, 16, 54 |
| | | SFG_L(R)_7_2 | 3 | 4 | *A8dl, dorsolateral area 8* | −18, 24, 53 | 22, 26, 51 |
| | | SFG_L(R)_7_3 | 5 | 6 | *A9l, lateral area 9* | −11, 49, 40 | 13, 48, 40 |
| | | SFG_L(R)_7_4 | 7 | 8 | *A6dl, dorsolateral area 6* | −18, −1, 65 | 20, 4, 64 |
| | | SFG_L(R)_7_5 | 9 | 10 | *A6m, medial area 6* | −6, −5, 58 | 7, −4, 60 |
| | | SFG_L(R)_7_6 | 11 | 12 | *A9m,medial area 9* | −5, 36, 38 | 6, 38, 35 |
| | | SFG_L(R)_7_7 | 13 | 14 | *A10m, medial area 10* | −8, 56, 15 | 8, 58, 13 |
| | MFG, Middle Frontal Gyrus | MFG_L(R)_7_1 | 15 | 16 | *A9/46d, dorsal area 9/46* | −27, 43, 31 | 30, 37, 36 |
| | | MFG_L(R)_7_2 | 17 | 18 | *IFJ, inferior frontal junction* | −42, 13, 36 | 42, 11, 39 |
| | | MFG_L(R)_7_3 | 19 | 20 | *A46, area 46* | −28, 56, 12 | 28, 55, 17 |
| | | MFG_L(R)_7_4 | 21 | 22 | *A9/46v, ventral area 9/46* | −41, 41, 16 | 42, 44, 14 |
| | | MFG_L(R)_7_5 | 23 | 24 | *A8vl, ventrolateral area 8* | −33, 23, 45 | 42, 27, 39 |
| | | MFG_L(R)_7_6 | 25 | 26 | *A6vl, ventrolateral area 6* | −32, 4, 55 | 34, 8, 54 |
| | | MFG_L(R)_7_7 | 27 | 28 | *A10l, lateral area10* | −26, 60, −6 | 25, 61, −4 |
| | IFG, Inferior Frontal Gyrus | IFG_L(R)_6_1 | 29 | 30 | *A44d,dorsal area 44* | −46, 13, 24 | 45, 16, 25 |
| | | IFG_L(R)_6_2 | 31 | 32 | *IFS, inferior frontal sulcus* | −47, 32, 14 | 48, 35, 13 |
| | | IFG_L(R)_6_3 | 33 | 34 | *A45c, caudal area 45* | −53, 23, 11 | 54, 24, 12 |
| | | IFG_L(R)_6_4 | 35 | 36 | *A45r, rostral area 45* | −49, 36, −3 | 51, 36, −1 |
| | | IFG_L(R)_6_5 | 37 | 38 | *A44op, opercular area 44* | −39, 23, 4 | 42, 22, 3 |

© 2024 Xu M et al. *JAMA Network Open*

| | | | | | | | |
|---|---|---|---|---|---|---|---|
| | | IFG_L(R)_6_6 | 39 | 40 | *A44v, ventral area 44* | −52, 13, 6 | 54, 14, 11 |
| | OrG, Orbital Gyrus | OrG_L(R)_6_1 | 41 | 42 | *A14m, medial area 14* | −7, 54, −7 | 6, 47, −7 |
| | | OrG_L(R)_6_2 | 43 | 44 | *A12/47o, orbital area 12/47* | −36, 33, −16 | 40, 39, −14 |
| | | OrG_L(R)_6_3 | 45 | 46 | *A11l, lateral area 11* | −23, 38, −18 | 23, 36, −18 |
| | | OrG_L(R)_6_4 | 47 | 48 | *A11m, medial area 11* | −6, 52, −19 | 6, 57, −16 |
| | | OrG_L(R)_6_5 | 49 | 50 | *A13, area 13* | −10, 18, −19 | 9, 20, −19 |
| | | OrG_L(R)_6_6 | 51 | 52 | *A12/47l, lateral area 12/47* | −41, 32, −9 | 42, 31, −9 |
| | PrG, Precentral Gyrus | PrG_L(R)_6_1 | 53 | 54 | *A4hf, area 4(head and face region)* | −49, −8, 39 | 55, −2, 33 |
| | | PrG_L(R)_6_2 | 55 | 56 | *A6cdl, caudal dorsolateral area 6* | −32, −9, 58 | 33, −7, 57 |
| | | PrG_L(R)_6_3 | 57 | 58 | *A4ul, area 4(upper limb region)* | −26, −25, 63 | 34, −19, 59 |
| | | PrG_L(R)_6_4 | 59 | 60 | *A4t, area 4(trunk region)* | −13, −20, 73 | 15, −22, 71 |
| | | PrG_L(R)_6_5 | 61 | 62 | *A4tl, area 4(tongue and larynx region)* | −52, 0, 8 | 54, 4, 9 |
| | | PrG_L(R)_6_6 | 63 | 64 | *A6cvl, caudal ventrolateral area 6* | −49, 5, 30 | 51, 7, 30 |
| | PCL, Paracentral Lobule | PCL_L(R)_2_1 | 65 | 66 | *A1/2/3ll, area1/2/3 (lower limb region)* | −8, −38, 58 | 10, −34, 54 |
| | | PCL_L(R)_2_2 | 67 | 68 | *A4ll, area 4, (lower limb region)* | −4, −23, 61 | 5, −21, 61 |
| Temporal Lobe | STG, Superior Temporal Gyrus | STG_L(R)_6_1 | 69 | 70 | *A38m, medial area 38* | −32, 14, −34 | 31, 15, −34 |
| | | STG_L(R)_6_2 | 71 | 72 | *A41/42, area 41/42* | −54, −32, 12 | 54, −24, 11 |
| | | STG_L(R)_6_3 | 73 | 74 | *TE1.0 and TE1.2* | −50, −11, 1 | 51, −4, −1 |
| | | STG_L(R)_6_4 | 75 | 76 | *A22c, caudal area 22* | −62, −33, 7 | 66, −20, 6 |
| | | STG_L(R)_6_5 | 77 | 78 | *A38l, lateral area 38* | −45, 11, −20 | 47, 12, −20 |
| | | STG_L(R)_6_6 | 79 | 80 | *A22r, rostral area 22* | −55, −3, −10 | 56, −12, −5 |
| | MTG, Middle Temporal Gyrus | MTG_L(R)_4_1 | 81 | 82 | *A21c, caudal area 21* | −65, −30, −12 | 65, −29, −13 |
| | | MTG_L(R)_4_2 | 83 | 84 | *A21r, rostral area 21* | −53, 2, −30 | 51, 6, −32 |

© 2024 Xu M et al. *JAMA Network Open*

| | | | | | | | |
|---|---|---|---|---|---|---|---|
| | | MTG_L(R)_4_3 | 85 | 86 | *A37dl, dorsolateral area37* | −59, −58, 4 | 60, −53, 3 |
| | | MTG_L(R)_4_4 | 87 | 88 | *aSTS, anterior superior temporal sulcus* | −58, −20, −9 | 58, −16, −10 |
| | ITG, Inferior Temporal Gyrus | ITG_L(R)_7_1 | 89 | 90 | *A20iv, intermediate ventral area 20* | −45, −26, −27 | 46, −14, −33 |
| | | ITG_L(R)_7_2 | 91 | 92 | *A37elv, extreme lateroventral area37* | −51, −57, −15 | 53, −52, −18 |
| | | ITG_L(R)_7_3 | 93 | 94 | *A20r, rostral area 20* | −43, −2, −41 | 40, 0, −43 |
| | | ITG_L(R)_7_4 | 95 | 96 | *A20il, intermediate lateral area 20* | −56, −16, −28 | 55, −11, −32 |
| | | ITG_L(R)_7_5 | 97 | 98 | *A37vl, ventrolateral area 37* | −55, −60, −6 | 54, −57, −8 |
| | | ITG_L(R)_7_6 | 99 | 100 | *A20cl, caudolateral of area 20* | −59, −42, −16 | 61, −40, −17 |
| | | ITG_L(R)_7_7 | 101 | 102 | *A20cv, caudoventral of area 20* | −55, −31, −27 | 54, −31, −26 |
| | FuG, Fusiform Gyrus | FuG_L(R)_3_1 | 103 | 104 | *A20rv, rostroventral area 20* | −33, −16, −32 | 33, −15, −34 |
| | | FuG_L(R)_3_2 | 105 | 106 | *A37mv, medioventral area37* | −31, −64, −14 | 31, −62, −14 |
| | | FuG_L(R)_3_3 | 107 | 108 | *A37lv, lateroventral area37* | −42, −51, −17 | 43, −49, −19 |
| | PhG, Parahippocampal Gyrus | PhG_L(R)_6_1 | 109 | 110 | *A35/36r, rostral area 35/36* | −27, −7, −34 | 28, −8, −33 |
| | | PhG_L(R)_6_2 | 111 | 112 | *A35/36c, caudal area 35/36* | −25, −25, −26 | 26, −23, −27 |
| | | PhG_L(R)_6_3 | 113 | 114 | *TL, area TL (lateral PPHC, posterior parahippocampal gyrus)* | −28, −32, −18 | 30, −30, −18 |
| | | PhG_L(R)_6_4 | 115 | 116 | *A28/34, area 28/34 (EC, entorhinal cortex)* | −19, −12, −30 | 19, −10, −30 |
| | | PhG_L(R)_6_5 | 117 | 118 | *TI, area TI(temporal agranular insular cortex)* | −23, 2, −32 | 22, 1, −36 |
| | | PhG_L(R)_6_6 | 119 | 120 | *TH, area TH (medial PPHC)* | −17, −39, −10 | 19, −36, −11 |
| | | pSTS_L(R)_2_1 | 121 | 122 | *rpSTS, rostroposterior superior temporal sulcus* | −54, −40, 4 | 53, −37, 3 |

© 2024 Xu M et al. *JAMA Network Open*

| | | | | | | | |
|---|---|---|---|---|---|---|---|
| | pSTS, posterior Superior Temporal Sulcus | pSTS_L(R)_2_2 | 123 | 124 | *cpSTS, caudoposterior superior temporal sulcus* | −52, −50, 11 | 57, −40, 12 |
| Parietal Lobe | SPL, Superior Parietal Lobule | SPL_L(R)_5_1 | 125 | 126 | *A7r, rostral area 7* | −16, −60, 63 | 19, −57, 65 |
| | | SPL_L(R)_5_2 | 127 | 128 | *A7c, caudal area 7* | −15, −71, 52 | 19, −69, 54 |
| | | SPL_L(R)_5_3 | 129 | 130 | *A5l, lateral area 5* | −33, −47, 50 | 35, −42, 54 |
| | | SPL_L(R)_5_4 | 131 | 132 | *A7pc, postcentral area 7* | −22, −47, 65 | 23, −43, 67 |
| | | SPL_L(R)_5_5 | 133 | 134 | *A7ip, intraparietal area 7(hIP3)* | −27, −59, 54 | 31, −54, 53 |
| | IPL, Inferior Parietal Lobule | IPL_L(R)_6_1 | 135 | 136 | *A39c, caudal area 39(PGp)* | −34, −80, 29 | 45, −71, 20 |
| | | IPL_L(R)_6_2 | 137 | 138 | *A39rd, rostrodorsal area 39(Hip3)* | −38, −61, 46 | 39, −65, 44 |
| | | IPL_L(R)_6_3 | 139 | 140 | *A40rd, rostrodorsal area 40(PFt)* | −51, −33, 42 | 47, −35, 45 |
| | | IPL_L(R)_6_4 | 141 | 142 | *A40c, caudal area 40(PFm)* | −56, −49, 38 | 57, −44, 38 |
| | | IPL_L(R)_6_5 | 143 | 144 | *A39rv, rostroventral area 39(PGa)* | −47, −65, 26 | 53, −54, 25 |
| | | IPL_L(R)_6_6 | 145 | 146 | *A40rv, rostroventral area 40(PFop)* | −53, −31, 23 | 55, −26, 26 |
| | Pcun, Precuneus | PCun_L(R)_4_1 | 147 | 148 | *A7m, medial area 7(PEp)* | −5, −63, 51 | 6, −65, 51 |
| | | PCun_L(R)_4_2 | 149 | 150 | *A5m, medial area 5(PEm)* | −8, −47, 57 | 7, −47, 58 |
| | | PCun_L(R)_4_3 | 151 | 152 | *dmPOS, dorsomedial parietooccipital sulcus(PEr)* | −12, −67, 25 | 16, −64, 25 |
| | | PCun_L(R)_4_4 | 153 | 154 | *A31, area 31 (Lc1)* | −6, −55, 34 | 6, −54, 35 |
| | PoG, Postcentral Gyrus | PoG_L(R)_4_1 | 155 | 156 | *A1/2/3ulhf, area 1/2/3(upper limb, head and face region)* | −50, −16, 43 | 50, −14, 44 |
| | | PoG_L(R)_4_2 | 157 | 158 | *A1/2/3tonIa, area 1/2/3(tongue and larynx region)* | −56, −14, 16 | 56, −10, 15 |
| | | PoG_L(R)_4_3 | 159 | 160 | *A2, area 2* | −46, −30, 50 | 48, −24, 48 |

© 2024 Xu M et al. *JAMA Network Open*

| | | | | | | | |
|---|---|---|---|---|---|---|---|
| | | PoG_L(R)_4_4 | 161 | 162 | *A1/2/3tru, area1/2/3(trunk region)* | −21, −35, 68 | 20, −33, 69 |
| Insular Lobe | INS, Insular Gyrus | INS_L(R)_6_1 | 163 | 164 | *G, hypergranular insula* | −36, −20, 10 | 37, −18, 8 |
| | | INS_L(R)_6_2 | 165 | 166 | *vIa, ventral agranular insula* | −32, 14, −13 | 33, 14, −13 |
| | | INS_L(R)_6_3 | 167 | 168 | *dIa, dorsal agranular insula* | −34, 18, 1 | 36, 18, 1 |
| | | INS_L(R)_6_4 | 169 | 170 | *vId/vIg, ventral dysgranular and granular insula* | −38, −4, −9 | 39, −2, −9 |
| | | INS_L(R)_6_5 | 171 | 172 | *dIg, dorsal granular insula* | −38, −8, 8 | 39, −7, 8 |
| | | INS_L(R)_6_6 | 173 | 174 | *dId, dorsal dysgranular insula* | −38, 5, 5 | 38, 5, 5 |
| Limbic Lobe | CG, Cingulate Gyrus | CG_L(R)_7_1 | 175 | 176 | *A23d, dorsal area 23* | −4, −39, 31 | 4, −37, 32 |
| | | CG_L(R)_7_2 | 177 | 178 | *A24rv, rostroventral area 24* | −3, 8, 25 | 5, 22, 12 |
| | | CG_L(R)_7_3 | 179 | 180 | *A32p, pregenual area 32* | −6, 34, 21 | 5, 28, 27 |
| | | CG_L(R)_7_4 | 181 | 182 | *A23v, ventral area 23* | −8, −47, 10 | 9, −44, 11 |
| | | CG_L(R)_7_5 | 183 | 184 | *A24cd, caudodorsal area 24* | −5, 7, 37 | 4, 6, 38 |
| | | CG_L(R)_7_6 | 185 | 186 | *A23c, caudal area 23* | −7, −23, 41 | 6, −20, 40 |
| | | CG_L(R)_7_7 | 187 | 188 | *A32sg, subgenual area 32* | −4, 39, −2 | 5, 41, 6 |
| Occipital Lobe | MVOcC, MedioVentral Occipital Cortex | MVOcC _L(R)_5_1 | 189 | 190 | *cLinG, caudal lingual gyrus* | −11, −82, −11 | 10, −85, −9 |
| | | MVOcC _L(R)_5_2 | 191 | 192 | *rCunG, rostral cuneus gyrus* | −5, −81, 10 | 7, −76, 11 |
| | | MVOcC _L(R)_5_3 | 193 | 194 | *cCunG, caudal cuneus gyrus* | −6, −94, 1 | 8, −90, 12 |
| | | MVOcC _L(R)_5_4 | 195 | 196 | *rLinG, rostral lingual gyrus* | −17, −60, −6 | 18, −60, −7 |
| | | MVOcC _L(R)_5_5 | 197 | 198 | *vmPOS,ventromedial parietooccipital sulcus* | −13, −68, 12 | 15, −63, 12 |
| | LOcC, lateral Occipital Cortex | LOcC_L(R)_4_1 | 199 | 200 | *mOccG, middle occipital gyrus* | −31, −89, 11 | 34, −86, 11 |
| | | LOcC _L(R)_4_2 | 201 | 202 | *V5/MT+, area V5/MT+* | −46, −74, 3 | 48, −70, −1 |
| | | LOcC _L(R)_4_3 | 203 | 204 | *OPC, occipital polar cortex* | −18, −99, 2 | 22, −97, 4 |

© 2024 Xu M et al. *JAMA Network Open*

| | | | | | | | |
|---|---|---|---|---|---|---|---|
| | | LOcC_L(R)_4_4 | 205 | 206 | *iOccG, inferior occipital gyrus* | −30, −88, −12 | 32, −85, −12 |
| | | LOcC _L(R)_2_1 | 207 | 208 | *msOccG, medial superior occipital gyrus* | −11, −88, 31 | 16, −85, 34 |
| | | LOcC _L(R)_2_2 | 209 | 210 | *lsOccG, lateral superior occipital gyrus* | −22, −77, 36 | 29, −75, 36 |
| Subcortical Nuclei | Amyg, Amygdala | Amyg_L(R)_2_1 | 211 | 212 | *mAmyg, medial amygdala* | −19, −2, −20 | 19, −2, −19 |
| | | Amyg_L(R)_2_2 | 213 | 214 | *lAmyg, lateral amygdala* | −27, −4, −20 | 28, −3, −20 |
| | Hipp, Hippocampus | Hipp_L(R)_2_1 | 215 | 216 | *rHipp, rostral hippocampus* | −22, −14, −19 | 22, −12, −20 |
| | | Hipp_L(R)_2_2 | 217 | 218 | *cHipp, caudal hippocampus* | −28, −30, −10 | 29, −27, −10 |
| | BG, Basal Ganglia | BG_L(R)_6_1 | 219 | 220 | *vCa, ventral caudate* | −12, 14, 0 | 15, 14, −2 |
| | | BG_L(R)_6_2 | 221 | 222 | *GP, globus pallidus* | −22, −2, 4 | 22, −2, 3 |
| | | BG_L(R)_6_3 | 223 | 224 | *NAC, nucleus accumbens* | −17, 3, −9 | 15, 8, −9 |
| | | BG_L(R)_6_4 | 225 | 226 | *vmPu, ventromedial putamen* | −23, 7, −4 | 22, 8, −1 |
| | | BG_L(R)_6_5 | 227 | 228 | *dCa, dorsal caudate* | −14, 2, 16 | 14, 5, 14 |
| | | BG_L(R)_6_6 | 229 | 230 | *dlPu, dorsolateral putamen* | −28, −5, 2 | 29, −3, 1 |
| | Tha, Thalamus | Tha_L(R)_8_1 | 231 | 232 | *mPFtha, medial pre-frontal thalamus* | −7, −12, 5 | 7, −11, 6 |
| | | Tha_L(R)_8_2 | 233 | 234 | *mPMtha, pre-motor thalamus* | −18, −13, 3 | 12, −14, 1 |
| | | Tha_L(R)_8_3 | 235 | 236 | *Stha, sensory thalamus* | −18, −23, 4 | 18, −22, 3 |
| | | Tha_L(R)_8_4 | 237 | 238 | *rTtha, rostral temporal thalamus* | −7, −14, 7 | 3, −13, 5 |
| | | Tha_L(R)_8_5 | 239 | 240 | *PPtha, posterior parietal thalamus* | −16, −24, 6 | 15, −25, 6 |
| | | Tha_L(R)_8_6 | 241 | 242 | *Otha, occipital thalamus* | −15, −28, 4 | 13, −27, 8 |
| | | Tha_L(R)_8_7 | 243 | 244 | *cTtha, caudal temporal thalamus* | −12, −22, 13 | 10, −14, 14 |
| | | Tha_L(R)_8_8 | 245 | 246 | *lPFtha, lateral pre-frontal thalamus* | −11, −14, 2 | 13, −16, 7 |

© 2024 Xu M et al. *JAMA Network Open*

**eTable 2**. Statistical analysis of sociodemographic and clinical characteristics among heathy controls without childhood trauma (HC), adolescent MDD with and without childhood trauma (CT+/CT−). Kruskal-Wallis's test (*H*) was used to analysis group differences of age, body mass index (BMI), and clinical scales. $\chi^2$ test was performed to analyze interactions between groups and categorical variables when each count ≥ 5, *i.e.*, gender, environmental variables, behavioral variables, and medication status; otherwise, Fisher's exact test was performed. MAD, median absolute deviation; NA, not applicable.

| **Variables** | **HCs (*n* = 91)** | **CT− (*n* = 62)** | **CT+ (*n* = 106)** | **Statistical analysis** | ***P* value** |
|---|---|---|---|---|---|
| **Demographic variables** | | | | | |
| Age (median [MAD], years) | 14.5 [2.0] | 16.2 [1.0] | 16.1 [1.4] | $H_{2,256}$ = 9.761 | .008 |
| Gender (Male/Female) | 35/56 | 22/40 | 22/84 | $\chi_2^2$ = 8.196 | .017 |
| BMI (median [MAD], kg/m$^2$) | 20.3 [2.4] | 20.1 [2.2] | 20.1 [1.9] | $H_{2,256}$ = 0.536 | .765 |
| **Environmental variables** | | | | | |
| Parents divorced (Yes/No) | 5/86 | 11/51 | 20/86 | $\chi_2^2$ = 8.323 | .016 |
| School bullying (Yes/No) | 5/86 | 14/48 | 56/50 | $\chi_2^2$ = 54.942 | < .001 |
| Major life events (Yes/No) | 13/78 | 14/48 | 47/59 | $\chi_2^2$ = 23.105 | < .001 |
| CTQ (median [MAD]) | 33.0 [4.0] | 34.5 [3.5] | 46.5 [5.5] | $H_{2,256}$ = 150.049 | < .001 |
| **Behavioral variables** | | | | | |
| Suicide attempt (Yes/No) | 0/91 | 14/48 | 41/65 | NA | < .001 |
| Non-suicidal self-injury (Yes/No) | 2/89 | 32/30 | 71/35 | NA | < .001 |
| Smoking (Yes/No) | 3/88 | 2/60 | 15/91 | NA | .006 |
| Drinking (Yes/No) | 7/84 | 5/57 | 15/91 | $\chi_2^2$ = 2.674 | .263 |
| **Clinical variables** | | | | | |
| HAMA (median [MAD]) | NA | 12.5 [4.5] | 16.0 [5.0] | $H_{1,166}$ = 5.875 | .015 |
| HAMD-17 (median [MAD]) | NA | 12.5 [4.0] | 19.0 [4.0] | $H_{1,166}$ = 4.419 | .036 |
| Medication history (Yes/No) | NA | 26/36 | 44/62 | $\chi_1^2$ = 0.003 | .957 |

**eTable 3**. Statistical analysis of sociodemographic and clinical characteristics among heathy controls without school bullying (HC), adolescent MDD with and without school bullying (SB+/SB−). Kruskal-Wallis's test (*H*) was used to analysis group differences of age, body mass index (BMI), and clinical scales. $\chi^2$ test was performed to analyze interactions between groups and categorical variables when each count ≥ 5, *i.e.*, gender, environmental variables, behavioral variables, and medication status; otherwise, Fisher's exact test was performed. MAD, median absolute deviation; NA, not applicable.

| **Variables** | **HCs ($n$ = 93)** | **SB− ($n$ = 98)** | **SB+ ($n$ = 70)** | **Statistical analysis** | ***P* value** |
|---|---|---|---|---|---|
| **Demographic variables** | | | | | |
| Age (median [MAD], years) | 14.5 [1.8] | 16.2 [0.9] | 16.1 [1.4] | $H_{2,258}$ = 12.346 | .002 |
| Gender (Male/Female) | 37/56 | 29/69 | 15/55 | $\chi_2^2$ = 6.440 | .040 |
| BMI (median [MAD], kg/m$^2$) | 20.3 [2.4] | 19.7 [2.3] | 20.3 [1.7] | $H_{2,258}$ = 3.214 | .201 |
| **Environmental variables** | | | | | |
| Parents divorced (Yes/No) | 3/90 | 13/85 | 18/52 | NA | < .001 |
| Childhood trauma (Yes/No) | 7/86 | 50/48 | 56/14 | $\chi_2^2$ = 89.260 | < .001 |
| Major life events (Yes/No) | 11/82 | 28/70 | 33/37 | $\chi_2^2$ = 25.011 | < .001 |
| CTQ (median [MAD]) | 34.0 [4.0] | 39.0 [6.0] | 45.0 [7.0] | $H_{2,258}$ = 60.455 | < .001 |
| **Behavioral variables** | | | | | |
| Suicide attempt (Yes/No) | 0/93 | 28/70 | 27/43 | NA | < .001 |
| Non-suicidal self-injury (Yes/No) | 1/92 | 54/44 | 49/21 | NA | < .001 |
| Smoking (Yes/No) | 3/90 | 8/90 | 9/61 | NA | < .071 |
| Drinking (Yes/No) | 7/86 | 10/88 | 10/60 | $\chi_2^2$ = 1.971 | .037 |
| **Clinical variables** | | | | | |
| HAMA (median [MAD]) | NA | 13.0 [4.0] | 16.0 [5.0] | $H_{1,166}$ = 4.060 | .044 |
| HAMD-17 (median [MAD]) | NA | 13.0 [4.0] | 19.0 [4.0] | $H_{1,166}$ = 1.172 | .279 |
| Medication history (Yes/No) | NA | 42/56 | 28/42 | $\chi_1^2$ = 0.137 | .711 |

© 2024 Xu M et al. *JAMA Network Open*

**eTable 4**. Statistical analysis of sociodemographic and clinical characteristics among heathy controls without major life events (HC), adolescent MDD with and without major life events (MLE+/MLE−). Kruskal-Wallis's test (*H*) was used to analysis group differences of age, body mass index (BMI), and clinical scales. $\chi^2$ test was performed to analyze interactions between groups and categorical variables when each count ≥ 5, *i.e.*, gender, environmental variables, behavioral variables, and medication status; otherwise, Fisher's exact test was performed. MAD, median absolute deviation; NA, not applicable.

| **Variables** | **HCs (*n* = 87)** | **MLE− (*n* = 107)** | **MLE+ (*n* = 61)** | **Statistical analysis** | ***P* value** |
|---|---|---|---|---|---|
| **Demographic variables** | | | | | |
| Age (median [MAD], years) | 14.8 [2.0] | 16.1 [1.3] | 16.3 [1.2] | $H_{2,252}$ = 10.637 | .005 |
| Gender (Male/Female) | 33/54 | 27/80 | 17/44 | $\chi_2^2$ = 3.876 | .144 |
| BMI (median [MAD], kg/m$^2$) | 20.0 [2.3] | 20.0 [2.4] | 20.2 [1.5] | $H_{2,252}$ = 2.054 | .358 |
| **Environmental variables** | | | | | |
| Parents divorced (Yes/No) | 3/84 | 12/95 | 19/42 | NA | < .001 |
| Childhood trauma (Yes/No) | 9/78 | 59/48 | 47/14 | $\chi_2^2$ = 71.948 | < .001 |
| School bullying (Yes/No) | 5/82 | 37/70 | 33/28 | $\chi_2^2$ = 42.749 | < .001 |
| CTQ (median [MAD]) | 34.0 [4.0] | 40.0 [5.0] | 45.0 [7.0] | $H_{2,252}$ = 51.059 | < .001 |
| **Behavioral variables** | | | | | |
| Suicide attempt (Yes/No) | 0/87 | 33/74 | 39/22 | NA | < .001 |
| Non-suicidal self-injury (Yes/No) | 1/86 | 65/42 | 38/23 | NA | < .001 |
| Smoking (Yes/No) | 2/85 | 6/101 | 11/50 | NA | .001 |
| Drinking (Yes/No) | 6/81 | 10/97 | 10/51 | $\chi_2^2$ = 3.678 | .159 |
| **Clinical variables** | | | | | |
| HAMA (median [MAD]) | NA | 15.0 [5.0] | 14.0 [4.0] | $H_{1,166}$ = 1.005 | .0316 |
| HAMD-17 (median [MAD]) | NA | 15.0 [5.0] | 17.0 [3.0] | $H_{1,166}$ = 0.915 | .339 |
| Medication history (Yes/No) | NA | 43/64 | 27/34 | $\chi_1^2$ = 0.266 | .606 |

© 2024 Xu M et al. *JAMA Network Open*

**eTable 5**. Statistical analysis of sociodemographic and clinical characteristics among heathy controls without suicidal attempt (HC), adolescent MDD with and without suicide attempt (SA+/SA−). Kruskal-Wallis's test (*H*) was used to analysis group differences of age, body mass index (BMI), and clinical scales. $\chi^2$ test was performed to analyze interactions between groups and categorical variables when each count ≥ 5, *i.e.*, gender, environmental variables, behavioral variables, and medication status; otherwise, Fisher's exact test was performed. MAD, median absolute deviation. NA, not applicable.

| **Variables** | **HCs (*n* = 101)** | **SA− (*n* = 113)** | **SA+ (*n* = 55)** | **Statistical analysis** | ***P* value** |
|---|---|---|---|---|---|
| **Demographic variables** | | | | | |
| Age (median [MAD], years) | 14.8 [2.0] | 16.3 [1.2] | 16.0 [1.1] | $H_{2,266} = 11.021$ | .004 |
| Gender (Male/Female) | 40/61 | 36/77 | 8/47 | $\chi^2_2 = 10.448$ | .005 |
| BMI (median [MAD], kg/m$^2$) | 20.3 [2.4] | 20.1 [2.2] | 19.8 [1.6] | $H_{2,266} = 1.182$ | .554 |
| **Environmental variables** | | | | | |
| Parents divorced (Yes/No) | 5/96 | 16/97 | 15/40 | $\chi^2_2 = 15.408$ | < .001 |
| Childhood trauma (Yes/No) | 8/93 | 43/70 | 27/28 | $\chi^2_2 = 37.079$ | < .001 |
| School bullying (Yes/No) | 10/91 | 65/48 | 41/14 | $\chi^2_2 = 77.143$ | < .001 |
| Major life events (Yes/No) | 14/87 | 39/74 | 22/33 | $\chi^2_2 = 16.362$ | < .001 |
| CTQ (median [MAD]) | 34.0 [4.0] | 41.0 [6.0] | 44.0 [7.0] | $H_{2,256} = 52.191$ | < .001 |
| **Behavioral variables** | | | | | |
| Non-suicidal self-injury (Yes/No) | 2/99 | 59/54 | 44/11 | NA | < .001 |
| Smoking (Yes/No) | 3/98 | 9/104 | 8/47 | NA | < .001 |
| Drinking (Yes/No) | 10/91 | 9/104 | 11/44 | $\chi^2_2 = 5.664$ | .059 |
| **Clinical variables** | | | | | |
| HAMA (median [MAD]) | NA | 13.0 [4.0] | 18.0 [5.0] | $H_{1,166} = 11.534$ | < .001 |
| HAMD-17 (median [MAD]) | NA | 13.0 [3.0] | 21.0 [4.0] | $H_{1,166} = 15.918$ | < .001 |
| Medication history (Yes/No) | NA | 45/68 | 25/30 | $\chi^2_1 = 0.483$ | .487 |

© 2024 Xu M et al. *JAMA Network Open*

**eTable 6**. Statistical analysis of sociodemographic and clinical characteristics among heathy controls without non−suicidal self−injurious behavior (HC), adolescent MDD with and without non−suicidal self−injurious behavior (NSSI+/NSSI−). Kruskal-Wallis's test (*H*) was used to analysis group differences of age, body mass index (BMI), and clinical scales. $\chi^2$ test was performed to analyze interactions between groups and categorical variables when each count ≥ 5, *i.e.*, gender, environmental variables, behavioral variables, and medication status; otherwise, Fisher's exact test was performed. MAD, median absolute deviation; NA, not applicable

| **Variables** | **HCs (*n* = 99)** | **NSSI− (*n* = 65)** | **NSSI+ (*n* = 103)** | **Statistical analysis** | ***P* value** |
|---|---|---|---|---|---|
| **Demographic variables** | | | | | |
| Age (median [MAD], years) | 14.8 [2.0] | 16.5 [1.0] | 15.9 [1.2] | $H_{2,264}$ = 14.750 | < .001 |
| Gender (Male/Female) | 40/59 | 29/36 | 15/88 | $\chi^2_2$ = 22.527 | < .001 |
| BMI (median [MAD], kg/m$^2$) | 20.3 [2.4] | 20.1 [1.7] | 20.1 [2.5] | $H_{2,264}$ = 1.116 | .572 |
| **Environmental variables** | | | | | |
| Parents divorced (Yes/No) | 5/94 | 11/54 | 20/83 | $\chi^2_2$ = 9.804 | .007 |
| Childhood trauma (Yes/No) | 10/89 | 35/30 | 71/32 | $\chi^2_2$ = 74.891 | < .001 |
| School bullying (Yes/No) | 7/92 | 21/44 | 49/54 | $\chi^2_2$ = 40.855 | < .001 |
| Major life events (Yes/No) | 13/86 | 23/42 | 38/65 | $\chi^2_2$ = 16.750 | < .001 |
| CTQ (median [MAD]) | 34.0 [4.0] | 40.0 [6.0] | 43.0 [6.0] | $H_{2,264}$ = 49.664 | < .001 |
| **Behavioral variables** | | | | | |
| Suicide attempt (Yes/No) | 0/99 | 11/54 | 44/59 | NA | < .001 |
| Smoking (Yes/No) | 3/96 | 6/59 | 11/92 | NA | < .001 |
| Drinking (Yes/No) | 10/89 | 5/60 | 15/88 | $\chi^2_2$ = 2.090 | .352 |
| **Clinical variables** | | | | | |
| HAMA (median [MAD]) | NA | 12.0 [4.0] | 16.0 [5.0] | $H_{1,166}$ = 8.545 | .003 |
| HAMD-17 (median [MAD]) | NA | 12.0 [4.0] | 19.0 [4.0] | $H_{1,166}$ = 16.771 | < .001 |
| Medication history (Yes/No) | NA | 22/43 | 48/55 | $\chi^2_1$ = 2.668 | .102 |

© 2024 Xu M et al. *JAMA Network Open*

**eTable 7.** Statistical analysis of differences between MDD and HCs. For abbreviations of anatomy and brain regions, please refer to eTable 1. Both FDR corrected p-value ($P_{adj}$) and original p-value (*P*) are provided.

| ID | Subregion | Anatomy | *T* | Cohen's *d* | 95% CI | *P* | $P_{adj}$ |
|---|---|---|---|---|---|---|---|
| 22 | A9/46v_R | MFG | 3.125 | 0.389 | [0.142, 0.635] | .002 | .032 |
| 86 | A37dl_R | MTG | 3.249 | 0.405 | [0.158, 0.651] | .001 | .025 |
| 120 | TH_R | PhG | 3.868 | 0.482 | [0.234, 0.729] | < .001 | .004 |
| 121 | rpSTS_L | pSTS | 3.169 | 0.395 | [0.148, 0.641] | .002 | .030 |
| 124 | cpSTS_R | pSTS | 3.273 | 0.408 | [0.161, 0.654] | .001 | .025 |
| 135 | A39c_L | IPL | 4.339 | 0.540 | [0.291, 0.788] | < .001 | .002 |
| 147 | A7m_L | Pcun | 3.259 | 0.406 | [0.159, 0.652] | .001 | .025 |
| 151 | dmPOS_L | Pcun | 3.495 | 0.435 | [0.188, 0.682] | .001 | .015 |
| 163 | G_L | INS | 4.246 | 0.529 | [0.280, 0.776] | < .001 | .002 |
| 164 | G_R | INS | 2.957 | 0.368 | [0.122, 0.614] | .003 | .049 |
| 172 | dIg_R | INS | 3.950 | 0.492 | [0.244, 0.739] | < .001 | .004 |
| 174 | dId_R | INS | 2.940 | 0.366 | [0.120, 0.612] | .004 | .049 |
| 182 | A23v_R | CG | 4.217 | 0.525 | [0.276, 0.773] | < .001 | .002 |
| 195 | rLinG_L | MVOcC | 3.985 | 0.496 | [0.248, 0.743] | < .001 | .004 |
| 197 | vmPOS_L | MVOcC | 4.668 | 0.581 | [0.332, 0.830] | < .001 | .001 |
| 198 | vmPOS_R | MVOcC | 3.263 | 0.406 | [0.159, 0.652] | .001 | .025 |
| 241 | Otha_L | Tha | 4.018 | 0.500 | [0.252, 0.748] | < .001 | .004 |
| 243 | cTtha_L | Tha | 2.938 | 0.366 | [0.119, 0.612] | .004 | .049 |

© 2024 Xu M et al. *JAMA Network Open*

**eTable 8.** The network definition in Brainnetome atlas.

| ID | Description | Subregion | Network | ID | Description | Subregion | Network |
|---|---|---|---|---|---|---|---|
| 1 | A8m | SFG_L_7_1 | FPN | 2 | A8m | SFG_R_7_1 | VAN |
| 3 | A8dl | SFG_L_7_2 | DMN | 4 | A8dl | SFG_R_7_2 | FPN |
| 5 | A9l | SFG_L_7_3 | DMN | 6 | A9l | SFG_R_7_3 | DMN |
| 7 | A6dl | SFG_L_7_4 | DAN | 8 | A6dl | SFG_R_7_4 | DAN |
| 9 | A6m | SFG_L_7_5 | SMN | 10 | A6m | SFG_R_7_5 | SMN |
| 11 | A9m | SFG_L_7_6 | DMN | 12 | A9m | SFG_R_7_6 | FPN |
| 13 | A10m | SFG_L_7_7 | DMN | 14 | A10m | SFG_R_7_7 | DMN |
| 15 | A9/46d | MFG_L_7_1 | VAN | 16 | A9/46d | MFG_R_7_1 | FPN |
| 17 | IFJ | MFG_L_7_2 | FPN | 18 | IFJ | MFG_R_7_2 | FPN |
| 19 | A46 | MFG_L_7_3 | FPN | 20 | A46 | MFG_R_7_3 | FPN |
| 21 | A9/46v | MFG_L_7_4 | FPN | 22 | A9/46v | MFG_R_7_4 | FPN |
| 23 | A8vl | MFG_L_7_5 | DMN | 24 | A8vl | MFG_R_7_5 | FPN |
| 25 | A6vl | MFG_L_7_6 | DAN | 26 | A6vl | MFG_R_7_6 | DAN |
| 27 | A10l | MFG_L_7_7 | LIM | 28 | A10l | MFG_R_7_7 | FPN |
| 29 | A44d | IFG_L_6_1 | FPN | 30 | A44d | IFG_R_6_1 | DAN |
| 31 | IFS | IFG_L_6_2 | FPN | 32 | IFS | IFG_R_6_2 | FPN |
| 33 | A45c | IFG_L_6_3 | DMN | 34 | A45c | IFG_R_6_3 | DMN |
| 35 | A45r | IFG_L_6_4 | DMN | 36 | A45r | IFG_R_6_4 | FPN |
| 37 | A44op | IFG_L_6_5 | VAN | 38 | A44op | IFG_R_6_5 | VAN |
| 39 | A44v | IFG_L_6_6 | VAN | 40 | A44v | IFG_R_6_6 | VAN |
| 41 | A14m | OrG_L_6_1 | DMN | 42 | A14m | OrG_R_6_1 | DMN |
| 43 | A12/47o | OrG_L_6_2 | DMN | 44 | A12/47o | OrG_R_6_2 | DMN |
| 45 | A11l | OrG_L_6_3 | LIM | 46 | A11l | OrG_R_6_3 | FPN |
| 47 | A11m | OrG_L_6_4 | LIM | 48 | A11m | OrG_R_6_4 | LIM |
| 49 | A13 | OrG_L_6_5 | LIM | 50 | A13 | OrG_R_6_5 | LIM |
| 51 | A12/47l | OrG_L_6_6 | DMN | 52 | A12/47l | OrG_R_6_6 | DMN |
| 53 | A4hf | PrG_L_6_1 | SMN | 54 | A4hf | PrG_R_6_1 | SMN |
| 55 | A6cdl | PrG_L_6_2 | DAN | 56 | A6cdl | PrG_R_6_2 | DAN |
| 57 | A4ul | PrG_L_6_3 | SMN | 58 | A4ul | PrG_R_6_3 | SMN |
| 59 | A4t | PrG_L_6_4 | SMN | 60 | A4t | PrG_R_6_4 | SMN |
| 61 | A4tl | PrG_L_6_5 | VAN | 62 | A4tl | PrG_R_6_5 | VAN |
| 63 | A6cvl | PrG_L_6_6 | DAN | 64 | A6cvl | PrG_R_6_6 | DAN |
| 65 | A1/2/3ll | PCL_L_2_1 | VAN | 66 | A1/2/3ll | PCL_R_2_1 | SMN |
| 67 | A4ll | PCL_L_2_2 | SMN | 68 | A4ll | PCL_R_2_2 | SMN |
| 69 | A38m | STG_L_6_1 | LIM | 70 | A38m | STG_R_6_1 | LIM |
| 71 | A41/42 | STG_L_6_2 | SMN | 72 | A41/42 | STG_R_6_2 | SMN |
| 73 | TE1.0/TE1.2 | STG_L_6_3 | SMN | 74 | TE1.0/TE1.2 | STG_R_6_3 | SMN |
| 75 | A22c | STG_L_6_4 | SMN | 76 | A22c | STG_R_6_4 | SMN |
| 77 | A38l | STG_L_6_5 | LIM | 78 | A38l | STG_R_6_5 | LIM |

| | | | | | | | |
|---|---|---|---|---|---|---|---|
| 79 | A22r | STG_L_6_6 | DMN | 80 | A22r | STG_R_6_6 | DMN |
| 81 | A21c | MTG_L_4_1 | DMN | 82 | A21c | MTG_R_4_1 | FPN |
| 83 | A21r | MTG_L_4_2 | DMN | 84 | A21r | MTG_R_4_2 | DMN |
| 85 | A37dl | MTG_L_4_3 | DAN | 86 | A37dl | MTG_R_4_3 | DAN |
| 87 | aSTS | MTG_L_4_4 | DMN | 88 | aSTS | MTG_R_4_4 | DMN |
| 89 | A20iv | ITG_L_7_1 | LIM | 90 | A20iv | ITG_R_7_1 | LIM |
| 91 | A37elv | ITG_L_7_2 | DAN | 92 | A37elv | ITG_R_7_2 | DAN |
| 93 | A20r | ITG_L_7_3 | LIM | 94 | A20r | ITG_R_7_3 | LIM |
| 95 | A20il | ITG_L_7_4 | DMN | 96 | A20il | ITG_R_7_4 | LIM |
| 97 | A37vl | ITG_L_7_5 | DAN | 98 | A37vl | ITG_R_7_5 | DAN |
| 99 | A20cl | ITG_L_7_6 | FPN | 100 | A20cl | ITG_R_7_6 | FPN |
| 101 | A20cv | ITG_L_7_7 | LIM | 102 | A20cv | ITG_R_7_7 | LIM |
| 103 | A20rv | FuG_L_3_1 | LIM | 104 | A20rv | FuG_R_3_1 | LIM |
| 105 | A37mv | FuG_L_3_2 | VIS | 106 | A37mv | FuG_R_3_2 | VIS |
| 107 | A37lv | FuG_L_3_3 | DAN | 108 | A37lv | FuG_R_3_3 | VIS |
| 109 | A35/36r | PhG_L_6_1 | LIM | 110 | A35/36r | PhG_R_6_1 | LIM |
| 111 | A35/36c | PhG_L_6_2 | LIM | 112 | A35/36c | PhG_R_6_2 | VIS |
| 113 | TL | PhG_L_6_3 | VIS | 114 | TL | PhG_R_6_3 | VIS |
| 115 | A28/34 | PhG_L_6_4 | LIM | 116 | A28/34 | PhG_R_6_4 | LIM |
| 117 | TI | PhG_L_6_5 | LIM | 118 | TI | PhG_R_6_5 | LIM |
| 119 | TH | PhG_L_6_6 | VIS | 120 | TH | PhG_R_6_6 | VIS |
| 121 | rpSTS | pSTS_L_2_1 | DMN | 122 | rpSTS | pSTS_R_2_1 | DMN |
| 123 | cpSTS | pSTS_L_2_2 | VAN | 124 | cpSTS | pSTS_R_2_2 | VAN |
| 125 | A7r | SPL_L_5_1 | DAN | 126 | A7r | SPL_R_5_1 | DAN |
| 127 | A7c | SPL_L_5_2 | DAN | 128 | A7c | SPL_R_5_2 | DAN |
| 129 | A5l | SPL_L_5_3 | DAN | 130 | A5l | SPL_R_5_3 | DAN |
| 131 | A7pc | SPL_L_5_4 | SMN | 132 | A7pc | SPL_R_5_4 | SMN |
| 133 | A7ip | SPL_L_5_5 | DAN | 134 | A7ip | SPL_R_5_5 | DAN |
| 135 | A39c | IPL_L_6_1 | VIS | 136 | A39c | IPL_R_6_1 | VIS |
| 137 | A39rd | IPL_L_6_2 | FPN | 138 | A39rd | IPL_R_6_2 | FPN |
| 139 | A40rd | IPL_L_6_3 | DAN | 140 | A40rd | IPL_R_6_3 | DAN |
| 141 | A40c | IPL_L_6_4 | DMN | 142 | A40c | IPL_R_6_4 | FPN |
| 143 | A39rv | IPL_L_6_5 | DAN | 144 | A39rv | IPL_R_6_5 | DMN |
| 145 | A40rv | IPL_L_6_6 | SMN | 146 | A40rv | IPL_R_6_6 | SMN |
| 147 | A7m | PCun_L_4_1 | FPN | 148 | A7m | PCun_R_4_1 | FPN |
| 149 | A5m | PCun_L_4_2 | SMN | 150 | A5m | PCun_R_4_2 | DAN |
| 151 | dmPOS | PCun_L_4_3 | VIS | 152 | dmPOS | PCun_R_4_3 | VIS |
| 153 | A31 | PCun_L_4_4 | DMN | 154 | A31 | PCun_R_4_4 | DMN |
| 155 | A1/2/3ulhf | PoG_L_4_1 | SMN | 156 | A1/2/3ulhf | PoG_R_4_1 | SMN |
| 157 | A1/2/3tonIa | PoG_L_4_2 | SMN | 158 | A1/2/3tonIa | PoG_R_4_2 | SMN |
| 159 | A2 | PoG_L_4_3 | DAN | 160 | A2 | PoG_R_4_3 | SMN |

© 2024 Xu M et al. *JAMA Network Open*

| | | | | | | | |
|---|---|---|---|---|---|---|---|
| 161 | A1/2/3tru | PoG_L_4_4 | SMN | 162 | A1/2/3tru | PoG_R_4_4 | SMN |
| 163 | G | INS_L_6_1 | SMN | 164 | G | INS_R_6_1 | SMN |
| 165 | vIa | INS_L_6_2 | SUB | 166 | vIa | INS_R_6_2 | FPN |
| 167 | dIa | INS_L_6_3 | VAN | 168 | dIa | INS_R_6_3 | VAN |
| 169 | vId/vIg | INS_L_6_4 | VAN | 170 | vId/vIg | INS_R_6_4 | VAN |
| 171 | dIg | INS_L_6_5 | SMN | 172 | dIg | INS_R_6_5 | SMN |
| 173 | dId | INS_L_6_6 | VAN | 174 | dId | INS_R_6_6 | VAN |
| 175 | A23d | CG_L_7_1 | DMN | 176 | A23d | CG_R_7_1 | DMN |
| 177 | A24rv | CG_L_7_2 | VAN | 178 | A24rv | CG_R_7_2 | DMN |
| 179 | A32p | CG_L_7_3 | DMN | 180 | A32p | CG_R_7_3 | VAN |
| 181 | A23v | CG_L_7_4 | DMN | 182 | A23v | CG_R_7_4 | VIS |
| 183 | A24cd | CG_L_7_5 | VAN | 184 | A24cd | CG_R_7_5 | VAN |
| 185 | A23c | CG_L_7_6 | VAN | 186 | A23c | CG_R_7_6 | VAN |
| 187 | A32sg | CG_L_7_7 | DMN | 188 | A32sg | CG_R_7_7 | DMN |
| 189 | cLinG | MVOcC_L_5_1 | VIS | 190 | cLinG | MVOcC_R_5_1 | VIS |
| 191 | rCunG | MVOcC_L_5_2 | VIS | 192 | rCunG | MVOcC_R_5_2 | VIS |
| 193 | cCunG | MVOcC_L_5_3 | VIS | 194 | cCunG | MVOcC_R_5_3 | VIS |
| 195 | rLinG | MVOcC_L_5_4 | VIS | 196 | rLinG | MVOcC_R_5_4 | VIS |
| 197 | vmPOS | MVOcC_L_5_5 | VIS | 198 | vmPOS | MVOcC_R_5_5 | VIS |
| 199 | mOccG | LOcC_L_4_1 | VIS | 200 | mOccG | LOcC_R_4_1 | VIS |
| 201 | V5/MT+ | LOcC_L_4_2 | DAN | 202 | V5/MT+ | LOcC_R_4_2 | VIS |
| 203 | OPC | LOcC_L_4_3 | VIS | 204 | OPC | LOcC_R_4_3 | VIS |
| 205 | iOccG | LOcC_L_4_4 | VIS | 206 | iOccG | LOcC_R_4_4 | VIS |
| 207 | msOccG | LOcC_L_2_1 | VIS | 208 | msOccG | LOcC_R_2_1 | VIS |
| 209 | lsOccG | LOcC_L_2_2 | VIS | 210 | lsOccG | LOcC_R_2_2 | VIS |
| 211 | mAmyg | Amyg_L_2_1 | SUB | 212 | mAmyg | Amyg_R_2_1 | SUB |
| 213 | lAmyg | Amyg_L_2_2 | SUB | 214 | lAmyg | Amyg_R_2_2 | SUB |
| 215 | rHipp | Hipp_L_2_1 | SUB | 216 | rHipp | Hipp_R_2_1 | SUB |
| 217 | cHipp | Hipp_L_2_2 | SUB | 218 | cHipp | Hipp_R_2_2 | SUB |
| 219 | vCa | BG_L_6_1 | SUB | 220 | vCa | BG_R_6_1 | SUB |
| 221 | GP | BG_L_6_2 | SUB | 222 | GP | BG_R_6_2 | SUB |
| 223 | NAC | BG_L_6_3 | SUB | 224 | NAC | BG_R_6_3 | SUB |
| 225 | vmPu | BG_L_6_4 | SUB | 226 | vmPu | BG_R_6_4 | SUB |
| 227 | dCa | BG_L_6_5 | SUB | 228 | dCa | BG_R_6_5 | SUB |
| 229 | dlPu | BG_L_6_6 | SUB | 230 | dlPu | BG_R_6_6 | SUB |
| 231 | mPFtha | Tha_L_8_1 | SUB | 232 | mPFtha | Tha_R_8_1 | SUB |
| 233 | mPMtha | Tha_L_8_2 | SUB | 234 | mPMtha | Tha_R_8_2 | SUB |
| 235 | Stha | Tha_L_8_3 | SUB | 236 | Stha | Tha_R_8_3 | SUB |
| 237 | rTtha | Tha_L_8_4 | SUB | 238 | rTtha | Tha_R_8_4 | SUB |
| 239 | PPtha | Tha_L_8_5 | SUB | 240 | PPtha | Tha_R_8_5 | SUB |
| 241 | Otha | Tha_L_8_6 | SUB | 242 | Otha | Tha_R_8_6 | SUB |

© 2024 Xu M et al. *JAMA Network Open*

| | | | | | | | |
|---|---|---|---|---|---|---|---|
| 243 | cTtha | Tha_L_8_7 | SUB | 244 | cTtha | Tha_R_8_7 | SUB |
| 245 | lPFtha | Tha_L_8_8 | SUB | 246 | lPFtha | Tha_R_8_8 | SUB |

(Abbreviations: VIS, visual network; SMN, somatomotor network; DAN, dorsal attention network; VAN, ventral attention network; LIM, limbic network; FPN, frontoparietal network; DMN, default mode network; SUB, subcortical network)

© 2024 Xu M et al. *JAMA Network Open*

**eTable 9. Demographic characteristics of the matched sample.** Wilcoxon rank sum test (*Z*) was used to analysis group differences of age and body mass index (BMI). $\chi^2$ test was performed to analyze the gender differences. MAD: median absolute deviation.

| **Variables** | **HCs (*n* = 101)** | **MDD (*n* = 168)** | **Statistical analysis** | ***P* value** |
|---|---|---|---|---|
| **Demographic variables** | | | | |
| Age (median [MAD], years) | 14.8 [2.0] | 15.9 [1.6] | $Z = 1.798$ | .072 |
| Gender (Male/Female) | 40/61 | 32/69 | $\chi_1^2 = 1.381$ | .240 |
| BMI (median [MAD], kg/m$^2$) | 20.3 [2.4] | 20.0 [2.3] | $Z = -0.738$ | .461 |

© 2024 Xu M et al. *JAMA Network Open*

**eTable 10.** Statistical analyses of SC−FC coupling differences among healthy controls, adolescent MDDs with and without suicidal attempt (SA+ and SA−). For abbreviations of anatomy and brain regions, please refer to eTable 1. Both FDR corrected p-value ($P_{adj}$) and original p-value ($P$) are provided.

| ID | Subregion | Anatomy | $F$ | Partial $\eta^2$ | 90% CI | $P$ | $P_{adj}$ |
|---|---|---|---|---|---|---|---|
| 111 | A35/36c_L | PhG | 9.571 | 0.069 | [0.025, 0.121] | < .001 | .007 |
| 120 | TH_R | PhG | 7.620 | 0.056 | [0.016, 0.104] | .001 | .017 |
| 121 | rpSTS_L | pSTS | 6.421 | 0.048 | [0.011, 0.093] | .002 | .042 |
| 135 | A39c_L | IPL | 11.046 | 0.079 | [0.031, 0.133] | < .001 | .003 |
| 147 | A7m_L | Pcun | 6.201 | 0.046 | [0.010, 0.091] | .002 | .048 |
| 151 | dmPOS_L | Pcun | 6.547 | 0.048 | [0.012, 0.094] | .002 | .041 |
| 163 | G_L | INS | 9.007 | 0.066 | [0.022, 0.116] | < .001 | .007 |
| 172 | dIg_R | INS | 9.020 | 0.066 | [0.022, 0.116] | < .001 | .007 |
| 182 | A23v_R | CG | 9.050 | 0.066 | [0.022, 0.117] | < .001 | .007 |
| 195 | rLinG_L | MVOcC | 8.619 | 0.063 | [0.002, 0.113] | < .001 | .008 |
| 197 | vmPOS_L | MVOcC | 11.236 | 0.080 | [0.032, 0.135] | < .001 | .003 |
| 241 | Otha_L | Tha | 8.491 | 0.062 | [0.020, 0.112] | < .001 | .008 |

© 2024 Xu M et al. *JAMA Network Open*

**eTable 11.** Post−hoc comparisons of SC−FC coupling differences among healthy controls, adolescent MDDs with and without suicidal attempt (SA+ and SA−). Both FDR corrected p-value ($P_{adj}$) and original p-value ($P$) are provided.

| **Subregion** | **Contrast** | ***T*** | **Cohen's *d*** | **95% CI** | ***P*** | $P_{adj}$ |
|---|---|---|---|---|---|---|
| A35/36c_L | SA− vs. HC | 0.537 | 0.067 | [−0.178, 0.312] | .853 | .853 |
| | SA+ vs. HC | −3.141 | −0.392 | [−0.638, −0.145] | .005 | .008 |
| | SA+ vs. SA− | −4.321 | −0.539 | [−0.787, −0.290] | < .001 | < .001 |
| TH_R | SA− vs. HC | 3.868 | 0.483 | [0.234, 0.730] | < .001 | .001 |
| | SA+ vs. HC | 2.751 | 0.343 | [0.097, 0.589] | .017 | .026 |
| | SA+ vs. SA− | −0.566 | −0.071 | [−0.315, 0.174] | .838 | .838 |
| rpSTS_L | SA− vs. HC | 2.597 | 0.324 | [0.078, 0.570] | .027 | .040 |
| | SA+ vs. HC | 3.536 | 0.441 | [0.193, 0.688] | .001 | .004 |
| | SA+ vs. SA− | 1.654 | 0.206 | [−0.039, 0.451] | .225 | .225 |
| A39c_L | SA− vs. HC | 3.698 | 0.461 | [0.213, 0.709] | .001 | .001 |
| | SA+ vs. HC | 4.544 | 0.567 | [0.317, 0.816] | < .001 | < .001 |
| | SA+ vs. SA− | 1.764 | 0.220 | [−0.025, 0.465] | .184 | .184 |
| A7m_L | SA− vs. HC | 2.770 | 0.346 | [0.099, 0.592] | .017 | .025 |
| | SA+ vs. HC | 3.405 | 0.425 | [0.177, 0.672] | .002 | .007 |
| | SA+ vs. SA− | 1.323 | 0.165 | [−0.08, 0.410] | .384 | .384 |
| dmPOS_L | SA− vs. HC | 3.098 | 0.386 | [0.139, 0.633] | .006 | .009 |
| | SA+ vs. HC | 3.359 | 0.419 | [0.172, 0.666] | .003 | .008 |
| | SA+ vs. SA− | 0.939 | 0.117 | [−0.128, 0.362] | .616 | .616 |
| G_L | SA− vs. HC | 4.135 | 0.516 | [0.267, 0.764] | < .001 | < .001 |
| | SA+ vs. HC | 3.257 | 0.406 | [0.159, 0.653] | .004 | .005 |
| | SA+ vs. SA− | −0.223 | −0.028 | [−0.272, 0.217] | .973 | .973 |
| dIg_R | SA− vs. HC | 3.381 | 0.422 | [0.174, 0.669] | .002 | .004 |
| | SA+ vs. HC | 4.089 | 0.510 | [0.261, 0.758] | < .001 | .001 |
| | SA+ vs. SA− | 1.534 | 0.191 | [−0.054, 0.436] | .277 | .277 |
| A23v_R | SA− vs. HC | 3.882 | 0.484 | [0.236, 0.732] | < .001 | .001 |
| | SA+ vs. HC | 3.729 | 0.465 | [0.217, 0.713] | .001 | .001 |
| | SA+ vs. SA− | 0.598 | 0.075 | [−0.170, 0.319] | .821 | .821 |
| rLinG_L | SA− vs. HC | 3.512 | 0.438 | [0.190, 0.685] | .002 | .002 |
| | SA+ vs. HC | 3.884 | 0.485 | [0.236, 0.732] | < .001 | .001 |
| | SA+ vs. SA− | 1.156 | 0.144 | [−0.101, 0.389] | .481 | .481 |
| vmPOS_L | SA− vs. HC | 4.250 | 0.530 | [0.281, 0.779] | < .001 | < .001 |
| | SA+ vs. HC | 4.236 | 0.529 | [0.279, 0.777] | < .001 | < .001 |
| | SA+ vs. SA− | 0.840 | 0.105 | [−0.140, 0.349] | .679 | .679 |
| Otha_L | SA− vs. HC | 4.115 | 0.513 | [0.264, 0.761] | < .001 | < .001 |
| | SA+ vs. HC | 2.661 | 0.332 | [0.085, 0.578] | .022 | .034 |
| | SA+ vs. SA− | −0.921 | −0.115 | [−0.360, 0.130] | .627 | .627 |

© 2024 Xu M et al. *JAMA Network Open*

**eTable 12.** Statistical analyses of SC−FC coupling differences among healthy controls, adolescent MDDs with and without non−suicidal self−injurious behaviors (NSSI+ and NSSI−). Both FDR corrected p-value ($P_{adj}$) and original p-value ($P$) are provided.

| **ID** | **Subregion** | **Anatomy** | ***F*** | **Partial $\eta^2$** | **90% CI** | ***P*** | **$P_{adj}$** |
|---|---|---|---|---|---|---|---|
| 22 | A9/46v_R | MFG | 8.225 | 0.061 | [0.019, 0.110] | < .001 | .010 |
| 86 | A37dl_R | MTG | 6.036 | 0.045 | [0.010, 0.090] | .003 | .048 |
| 120 | TH_R | PhG | 7.209 | 0.054 | [0.015, 0.101] | .001 | .020 |
| 135 | A39c_L | IPL | 10.367 | 0.075 | [0.028, 0.129] | < .001 | .005 |
| 151 | dmPOS_L | Pcun | 6.322 | 0.047 | [0.011, 0.093] | .002 | .040 |
| 163 | G_L | INS | 10.063 | 0.073 | [0.027, 0.126] | < .001 | .005 |
| 168 | dIa_R | INS | 8.189 | 0.060 | [0.019, 0.110] | < .001 | .010 |
| 172 | dIg_R | INS | 8.846 | 0.065 | [0.022, 0.116] | < .001 | .008 |
| 182 | A23v_R | CG | 8.915 | 0.065 | [0.022, 0.116] | < .001 | .008 |
| 195 | rLinG_L | MVOcC | 7.578 | 0.056 | [0.016, 0.104] | .001 | .016 |
| 197 | vmPOS_L | MVOcC | 11.805 | 0.085 | [0.035, 0.140] | < .001 | .003 |
| 198 | vmPOS_R | MVOcC | 7.002 | 0.052 | [0.014, 0.099] | .001 | .022 |
| 241 | Otha_L | Tha | 9.570 | 0.070 | [0.025, 0.122] | < .001 | .006 |
| 243 | cTtha_L | Tha | 8.283 | 0.061 | [0.019, 0.111] | < .001 | .010 |

© 2024 Xu M et al. *JAMA Network Open*

**eTable 13.** Post−hoc comparisons of SC−FC coupling differences among healthy controls, adolescent MDDs with and without non−suicidal self−injurious behaviors (NSSI+ and NSSI−). Both FDR corrected p-value ($P_{adj}$) and original p-value ($P$) are provided.

| Subregion | Contrast | *T* | Cohen's *d* | 90% CI | *P* | $P_{adj}$ |
|---|---|---|---|---|---|---|
| A9/46v_R | NSSI− vs. HC | 4.029 | 0.505 | [0.255, 0.753] | < .001 | .001 |
| | NSSI+ vs. HC | 1.951 | 0.244 | [−0.002, 0.491] | .127 | .127 |
| | NSSI+ vs. NSSI− | −2.477 | −0.310 | [−0.557, −0.063] | .037 | .055 |
| A37dl_R | NSSI− vs. HC | 3.435 | 0.430 | [0.182, 0.678] | .002 | .006 |
| | NSSI+ vs. HC | 2.414 | 0.302 | [0.055, 0.549] | .043 | .065 |
| | NSSI+ vs. NSSI− | −1.323 | −0.166 | [−0.411, 0.080] | .384 | .384 |
| TH_R | NSSI− vs. HC | 3.215 | 0.403 | [0.154, 0.650] | .004 | .006 |
| | NSSI+ vs. HC | 3.509 | 0.440 | [0.191, 0.688] | .002 | .005 |
| | NSSI+ vs. NSSI− | 0.077 | 0.010 | [−0.236, 0.255] | .997 | .997 |
| A39c_L | NSSI− vs. HC | 4.306 | 0.539 | [0.289, 0.789] | < .001 | < .001 |
| | NSSI+ vs. HC | 3.701 | 0.464 | [0.214, 0.712] | .001 | .001 |
| | NSSI+ vs. NSSI− | −0.948 | −0.119 | [−0.364, 0.127] | .611 | .611 |
| dmPOS_L | NSSI− vs. HC | 3.178 | 0.398 | [0.150, 0.646] | .005 | .008 |
| | NSSI+ vs. HC | 3.141 | 0.393 | [0.145, 0.641] | .005 | .008 |
| | NSSI+ vs. NSSI− | −0.270 | −0.034 | [−0.279, 0.212] | .961 | .961 |
| G_L | NSSI− vs. HC | 4.309 | 0.540 | [0.289, 0.789] | < .001 | < .001 |
| | NSSI+ vs. HC | 3.514 | 0.440 | [0.191, 0.688] | .002 | .002 |
| | NSSI+ vs. NSSI− | −1.149 | −0.144 | [−0.390, 0.102] | .485 | .485 |
| dIa_R | NSSI− vs. HC | 3.779 | 0.473 | [0.224, 0.722] | .001 | .002 |
| | NSSI+ vs. HC | 1.010 | 0.127 | [−0.119, 0.372] | .571 | .571 |
| | NSSI+ vs. NSSI− | −3.186 | −0.399 | [−0.647, −0.151] | .005 | .007 |
| dIg_R | NSSI− vs. HC | 2.344 | 0.294 | [0.046, 0.540] | .052 | .078 |
| | NSSI+ vs. HC | 4.205 | 0.527 | [0.276, 0.776] | < .001 | < .001 |
| | NSSI+ vs. NSSI− | 1.787 | 0.224 | [−0.023, 0.47] | .176 | .176 |
| A23v_R | NSSI− vs. HC | 3.832 | 0.480 | [0.231, 0.728] | < .001 | .001 |
| | NSSI+ vs. HC | 3.666 | 0.459 | [0.210, 0.707] | .001 | .001 |
| | NSSI+ vs. NSSI− | −0.453 | −0.057 | [−0.302, 0.189] | .893 | .893 |
| rLinG_L | NSSI− vs. HC | 2.513 | 0.315 | [0.067, 0.561] | .034 | .050 |
| | NSSI+ vs. HC | 3.879 | 0.486 | [0.236, 0.734] | < .001 | .001 |
| | NSSI+ vs. NSSI− | 1.255 | 0.157 | [−0.089, 0.403] | .422 | .422 |
| vmPOS_L | NSSI− vs. HC | 3.526 | 0.442 | [0.193, 0.690] | .001 | .002 |
| | NSSI+ vs. HC | 4.768 | 0.597 | [0.346, 0.847] | < .001 | < .001 |
| | NSSI+ vs. NSSI− | 1.050 | 0.132 | [−0.114, 0.377] | .546 | .546 |
| vmPOS_R | NSSI− vs. HC | 2.181 | 0.273 | [0.026, 0.520] | .076 | .115 |
| | NSSI+ vs. HC | 3.742 | 0.469 | [0.219, 0.717] | .001 | .002 |
| | NSSI+ vs. NSSI− | 1.484 | 0.186 | [−0.060, 0.432] | .300 | .300 |

© 2024 Xu M et al. *JAMA Network Open*

| | | | | | | |
|---|---|---|---|---|---|---|
| Otha_L | NSSI− vs. HC | 4.359 | 0.546 | [0.295, 0.795] | < .001 | < .001 |
| | NSSI+ vs. HC | 2.833 | 0.355 | [0.107, 0.602] | .014 | .021 |
| | NSSI+ vs. NSSI− | −1.921 | −0.241 | [−0.487, 0.006] | .135 | .135 |
| cTtha_L | NSSI− vs. HC | 4.022 | 0.504 | [0.255, 0.753] | < .001 | .001 |
| | NSSI+ vs. HC | 1.820 | 0.228 | [−0.018, 0.474] | .165 | .165 |
| | NSSI+ vs. NSSI− | −2.607 | −0.326 | [−0.573, −0.079] | .026 | .039 |

© 2024 Xu M et al. *JAMA Network Open*

**eTable 14.** Statistical analyses of SC−FC coupling differences among healthy controls, adolescent MDDs with and without childhood trauma (CT+ and CT−). Both FDR corrected p-value ($P_{adj}$) and original p-value (*P*) are provided.

| **ID** | **Subregion** | **Anatomy** | ***F*** | **Patial $\eta^2$** | **90% CI** | ***P*** | **$P_{adj}$** |
|---|---|---|---|---|---|---|---|
| 120 | TH_R | PhG | 7.723 | 0.059 | [0.017, 0.109] | .001 | .023 |
| 135 | A39c_L | IPL | 10.117 | 0.076 | [0.028, 0.130] | < .001 | .007 |
| 163 | G_L | INS | 8.360 | 0.063 | [0.020, 0.115] | < .001 | .017 |
| 172 | dIg_R | INS | 7.340 | 0.056 | [0.016, 0.105] | .001 | .028 |
| 182 | A23v_R | CG | 7.040 | 0.054 | [0.014, 0.102] | .001 | .033 |
| 195 | rLinG_L | MVOcC | 8.243 | 0.063 | [0.019, 0.114] | < .001 | .017 |
| 197 | vmPOS_L | MVOcC | 11.424 | 0.085 | [0.034, 0.141] | < .001 | .004 |
| 241 | Otha_L | Tha | 8.902 | 0.067 | [0.022, 0.119] | < .001 | .015 |

© 2024 Xu M et al. *JAMA Network Open*

**eTable 15.** Post−hoc comparisons of SC−FC coupling differences among healthy controls, adolescent MDDs with and without childhood trauma (CT+ and CT−). Both FDR corrected p-value ($P_{adj}$) and original p-value (*P*) are provided.

| **Subregion** | **Contrast** | ***T*** | **Cohen's *d*** | **95% CI** | ***P*** | $P_{adj}$ |
|---|---|---|---|---|---|---|
| TH_R | CT− vs. HC | 3.842 | 0.489 | [0.235, 0.742] | < .001 | .001 |
| | CT+ vs. HC | 3.015 | 0.384 | [0.132, 0.635] | .008 | .012 |
| | CT+ vs. CT− | −1.212 | −0.154 | [−0.404, 0.096] | .447 | .447 |
| A39c_L | CT− vs. HC | 4.140 | 0.527 | [0.273, 0.780] | < .001 | < .001 |
| | CT+ vs. HC | 3.933 | 0.500 | [0.247, 0.753] | < .001 | < .001 |
| | CT+ vs. CT− | −0.555 | −0.071 | [−0.320, 0.179] | .844 | .844 |
| G_L | CT− vs. HC | 3.223 | 0.410 | [0.158, 0.662] | .004 | .006 |
| | CT+ vs. HC | 3.966 | 0.505 | [0.251, 0.758] | < .001 | .001 |
| | CT+ vs. CT− | 0.559 | 0.071 | [−0.178, 0.321] | .842 | .842 |
| dIg_R | CT− vs. HC | 2.951 | 0.376 | [0.124, 0.627] | .010 | .015 |
| | CT+ vs. HC | 3.742 | 0.476 | [0.223, 0.729] | .001 | .002 |
| | CT+ vs. CT− | 0.633 | 0.081 | [−0.169, 0.330] | .802 | .802 |
| A23v_R | CT− vs. HC | 3.170 | 0.403 | [0.151, 0.655] | .005 | .007 |
| | CT+ vs. HC | 3.532 | 0.449 | [0.196, 0.702] | .001 | .004 |
| | CT+ vs. CT− | 0.146 | 0.019 | [−0.231, 0.268] | .988 | .988 |
| rLinG_L | CT− vs. HC | 3.830 | 0.487 | [0.234, 0.740] | < .001 | .001 |
| | CT+ vs. HC | 3.418 | 0.435 | [0.182, 0.687] | .002 | .003 |
| | CT+ vs. CT− | −0.755 | −0.096 | [−0.346, 0.154] | .731 | .731 |
| vmPOS_L | CT− vs. HC | 4.203 | 0.535 | [0.281, 0.788] | < .001 | < .001 |
| | CT+ vs. HC | 4.379 | 0.557 | [0.302, 0.811] | < .001 | < .001 |
| | CT+ vs. CT− | −0.141 | −0.018 | [−0.267, 0.232] | .989 | .989 |
| Otha_L | CT− vs. HC | 3.194 | 0.406 | [0.154, 0.658] | .005 | .007 |
| | CT+ vs. HC | 4.138 | 0.527 | [0.272, 0.780] | < .001 | < .001 |
| | CT+ vs. CT− | 0.782 | 0.100 | [−0.150, 0.349] | .714 | .714 |

© 2024 Xu M et al. *JAMA Network Open*

**eTable 16.** Statistical analyses of SC−FC coupling differences among healthy controls, adolescent MDDs with and without major life events (MLE+ and MLE−). Both FDR corrected p-value ($P_{adj}$) and original p-value ($P$) are provided.

| ID | Subregion | Anatomy | $F$ | Patial $\eta^2$ | 90% CI | $P$ | $P_{adj}$ |
|---|---|---|---|---|---|---|---|
| 13 | A10m_L | SFG | 8.92 | 0.068 | [0.023, 0.121] | < .001 | .007 |
| 23 | A8vl_L | MFG | 7.429 | 0.058 | [0.016, 0.108] | .001 | .017 |
| 41 | A14m_L | OrG | 6.977 | 0.054 | [0.014, 0.103] | .001 | .021 |
| 42 | A14m_R | OrG | 5.839 | 0.046 | [0.010, 0.092] | .003 | .036 |
| 86 | A37dl_R | MTG | 9.503 | 0.073 | [0.026, 0.127] | < .001 | .007 |
| 120 | TH_R | PhG | 6.573 | 0.051 | [0.013, 0.099] | .002 | .024 |
| 121 | rpSTS_L | pSTS | 5.795 | 0.046 | [0.009, 0.091] | .003 | .036 |
| 124 | cpSTS_R | pSTS | 6.047 | 0.047 | [0.010, 0.094] | .003 | .033 |
| 130 | A5l_R | SPL | 6.013 | 0.047 | [0.010, 0.094] | .003 | .033 |
| 135 | A39c_L | IPL | 9.500 | 0.073 | [0.026, 0.127] | < .001 | .007 |
| 147 | A7m_L | Pcun | 6.919 | 0.054 | [0.014, 0.103] | .001 | .021 |
| 151 | dmPOS_L | Pcun | 6.733 | 0.053 | [0.013, 0.101] | .001 | .022 |
| 153 | A31_L | Pcun | 6.751 | 0.053 | [0.013, 0.101] | .001 | .022 |
| 163 | G_L | INS | 7.309 | 0.057 | [0.016, 0.106] | .001 | .017 |
| 169 | vId/vIg_L | INS | 5.687 | 0.045 | [0.009, 0.090] | .004 | .038 |
| 172 | dIg_R | INS | 8.939 | 0.069 | [0.023, 0.122] | < .001 | .007 |
| 182 | A23v_R | CG | 8.625 | 0.066 | [0.022, 0.119] | < .001 | .008 |
| 187 | A32sg_L | CG | 8.427 | 0.065 | [0.021, 0.117] | < .001 | .009 |
| 195 | rLinG_L | MVOcC | 7.987 | 0.062 | [0.019, 0.113] | < .001 | .012 |
| 197 | vmPOS_L | MVOcC | 12.398 | 0.093 | [0.039, 0.151] | < .001 | .002 |
| 198 | vmPOS_R | MVOcC | 5.939 | 0.047 | [0.010, 0.093] | .003 | .034 |
| 214 | lAmyg_R | Amyg | 6.092 | 0.048 | [0.011, 0.094] | .003 | .033 |
| 234 | mPMtha_R | Tha | 7.757 | 0.060 | [0.018, 0.111] | .001 | .013 |
| 241 | Otha_L | Tha | 10.077 | 0.077 | [0.028, 0.132] | < .001 | .007 |
| 243 | cTtha_L | Tha | 6.086 | 0.048 | [0.011, 0.094] | .003 | .033 |

© 2024 Xu M et al. *JAMA Network Open*

**eTable 17.** Post−hoc comparisons of SC−FC coupling differences among healthy controls, adolescent MDDs with and without major life events (MLE+ and MLE−). Both FDR corrected p-value ($P_{adj}$) and original p-value ($P$) are provided.

| **Subregion** | **Constrast** | ***T*** | **Cohen's *d*** | **95% CI** | ***P*** | $P_{adj}$ |
|---|---|---|---|---|---|---|
| A10m_L | MLE− vs. HC | −0.773 | −0.099 | [−0.351, 0.153] | .720 | .720 |
| | MLE+ vs. HC | 2.743 | 0.352 | [0.098, 0.605] | .018 | .027 |
| | MLE+ vs. MLE− | 4.193 | 0.538 | [0.281, 0.793] | < .001 | < .001 |
| A8vl_L | MLE− vs. HC | −1.268 | −0.163 | [−0.414, 0.089] | .415 | .415 |
| | MLE+ vs. HC | 2.034 | 0.261 | [0.008, 0.513] | .106 | .160 |
| | MLE+ vs. MLE− | 3.853 | 0.494 | [0.239, 0.749] | < .001 | .001 |
| A14m_L | MLE− vs. HC | −0.885 | −0.114 | [−0.365, 0.138] | .650 | .650 |
| | MLE+ vs. HC | 2.266 | 0.291 | [0.038, 0.543] | .063 | .094 |
| | MLE+ vs. MLE− | 3.728 | 0.478 | [0.223, 0.733] | .001 | .002 |
| A14m_R | MLE− vs. HC | −1.240 | −0.159 | [−0.411, 0.093] | .431 | .431 |
| | MLE+ vs. HC | 1.697 | 0.218 | [−0.035, 0.47] | .208 | .313 |
| | MLE+ vs. MLE− | 3.410 | 0.437 | [0.183, 0.691] | .002 | .007 |
| A37dl_R | MLE− vs. HC | 2.239 | 0.287 | [0.034, 0.540] | .067 | .067 |
| | MLE+ vs. HC | 4.316 | 0.554 | [0.297, 0.809] | < .001 | < .001 |
| | MLE+ vs. MLE− | 2.897 | 0.372 | [0.118, 0.625] | .011 | .017 |
| TH_R | MLE− vs. HC | 3.430 | 0.440 | [0.185, 0.694] | .002 | .006 |
| | MLE+ vs. HC | 3.071 | 0.394 | [0.140, 0.647] | .007 | .010 |
| | MLE+ vs. MLE− | 0.092 | 0.012 | [−0.240, 0.263] | .995 | .995 |
| rpSTS_L | MLE− vs. HC | 2.742 | 0.352 | [0.098, 0.605] | .018 | .027 |
| | MLE+ vs. HC | 3.293 | 0.422 | [0.168, 0.676] | .003 | .010 |
| | MLE+ vs. MLE− | 1.102 | 0.141 | [−0.111, 0.393] | .514 | .514 |
| cpSTS_R | MLE− vs. HC | 2.024 | 0.260 | [0.007, 0.512] | .109 | .109 |
| | MLE+ vs. HC | 3.472 | 0.445 | [0.19, 0.700] | .002 | .005 |
| | MLE+ vs. MLE− | 2.090 | 0.268 | [0.015, 0.520] | .094 | .109 |
| A5l_R | MLE− vs. HC | 1.058 | 0.136 | [−0.116, 0.387] | .541 | .541 |
| | MLE+ vs. HC | 3.233 | 0.415 | [0.160, 0.669] | .004 | .012 |
| | MLE+ vs. MLE− | 2.833 | 0.363 | [0.110, 0.617] | .014 | .021 |
| A39c_L | MLE− vs. HC | 3.967 | 0.509 | [0.253, 0.764] | < .001 | .001 |
| | MLE+ vs. HC | 3.899 | 0.500 | [0.244, 0.755] | < .001 | .001 |
| | MLE+ vs. MLE− | 0.534 | 0.069 | [−0.183, 0.320] | .855 | .855 |
| A7m_L | MLE− vs. HC | 2.982 | 0.383 | [0.128, 0.636] | .009 | .013 |
| | MLE+ vs. HC | 3.604 | 0.462 | [0.207, 0.717] | .001 | .003 |
| | MLE+ vs. MLE− | 1.226 | 0.157 | [−0.095, 0.409] | .439 | .439 |
| dmPOS_L | MLE− vs. HC | 3.602 | 0.462 | [0.207, 0.717] | .001 | .003 |
| | MLE+ vs. HC | 2.809 | 0.360 | [0.107, 0.614] | .015 | .022 |
| | MLE+ vs. MLE− | −0.413 | −0.053 | [−0.304, 0.199] | .910 | .910 |

© 2024 Xu M et al. *JAMA Network Open*

| | | | | | | |
|---|---|---|---|---|---|---|
| A31_L | MLE− vs. HC | 0.640 | 0.082 | [−0.170, 0.334] | .798 | .798 |
| | MLE+ vs. HC | 3.216 | 0.413 | [0.158, 0.666] | .004 | .006 |
| | MLE+ vs. MLE− | 3.260 | 0.418 | [0.164, 0.672] | .004 | .006 |
| G_L | MLE− vs. HC | 3.294 | 0.423 | [0.168, 0.676] | .003 | .005 |
| | MLE+ vs. HC | 3.580 | 0.459 | [0.204, 0.714] | .001 | .004 |
| | MLE+ vs. MLE− | 0.863 | 0.111 | [−0.141, 0.362] | .664 | .664 |
| vId/vIg_L | MLE− vs. HC | 1.177 | 0.151 | [−0.101, 0.403] | .468 | .468 |
| | MLE+ vs. HC | 3.198 | 0.41 | [0.156, 0.664] | .004 | .013 |
| | MLE+ vs. MLE− | 2.662 | 0.341 | [0.088, 0.594] | .023 | .034 |
| dIg_R | MLE− vs. HC | 3.531 | 0.453 | [0.198, 0.707] | .001 | .002 |
| | MLE+ vs. HC | 4.029 | 0.517 | [0.261, 0.772] | < .001 | .001 |
| | MLE+ vs. MLE− | 1.159 | 0.149 | [−0.103, 0.400] | .479 | .479 |
| A23v_R | MLE− vs. HC | 3.615 | 0.464 | [0.209, 0.718] | .001 | .002 |
| | MLE+ vs. HC | 3.863 | 0.496 | [0.240, 0.750] | < .001 | .001 |
| | MLE+ vs. MLE− | 0.865 | 0.111 | [−0.141, 0.363] | .663 | .663 |
| A32sg_L | MLE− vs. HC | −1.801 | −0.231 | [−0.483, 0.021] | .171 | .194 |
| | MLE+ vs. HC | 1.737 | 0.223 | [−0.030, 0.475] | .194 | .194 |
| | MLE+ vs. MLE− | 4.060 | 0.521 | [0.265, 0.776] | < .001 | .001 |
| rLinG_L | MLE− vs. HC | 3.426 | 0.44 | [0.185, 0.694] | .002 | .003 |
| | MLE+ vs. HC | 3.754 | 0.482 | [0.226, 0.736] | .001 | .002 |
| | MLE+ vs. MLE− | 0.935 | 0.120 | [−0.132, 0.372] | .619 | .619 |
| vmPOS_L | MLE− vs. HC | 4.587 | 0.589 | [0.331, 0.845] | < .001 | < .001 |
| | MLE+ vs. HC | 4.390 | 0.563 | [0.306, 0.819] | < .001 | < .001 |
| | MLE+ vs. MLE− | 0.471 | 0.060 | [−0.191, 0.312] | .885 | .885 |
| vmPOS_R | MLE− vs. HC | 3.398 | 0.436 | [0.181, 0.690] | .002 | .007 |
| | MLE+ vs. HC | 2.583 | 0.331 | [0.078, 0.584] | .028 | .042 |
| | MLE+ vs. MLE− | −0.472 | −0.061 | [−0.312, 0.191] | .884 | .884 |
| lAmyg_R | MLE− vs. HC | −1.169 | −0.150 | [−0.402, 0.102] | .473 | .473 |
| | MLE+ vs. HC | 1.823 | 0.234 | [−0.019, 0.486] | .164 | .247 |
| | MLE+ vs. MLE− | 3.488 | 0.448 | [0.192, 0.702] | .002 | .005 |
| mPMtha_R | MLE− vs. HC | 1.181 | 0.152 | [−0.100, 0.403] | .466 | .466 |
| | MLE+ vs. HC | 3.664 | 0.470 | [0.215, 0.725] | .001 | .003 |
| | MLE+ vs. MLE− | 3.230 | 0.414 | [0.160, 0.668] | .004 | .006 |
| Otha_L | MLE− vs. HC | 2.977 | 0.382 | [0.128, 0.635] | .009 | .013 |
| | MLE+ vs. HC | 4.485 | 0.575 | [0.318, 0.831] | < .001 | < .001 |
| | MLE+ vs. MLE− | 2.312 | 0.297 | [0.044, 0.549] | .056 | .056 |
| cTtha_L | MLE− vs. HC | 2.421 | 0.311 | [0.057, 0.563] | .043 | .064 |
| | MLE+ vs. HC | 3.476 | 0.446 | [0.191, 0.700] | .002 | .005 |
| | MLE+ vs. MLE− | 1.669 | 0.214 | [−0.038, 0.466] | .219 | .219 |

© 2024 Xu M et al. *JAMA Network Open*

**eTable 18.** Statistical analyses of SC−FC coupling differences among healthy controls, adolescent MDDs with and without major school bullying (SB+ and SB−). Both FDR corrected p-value ($P_{adj}$) and original p-value ($P$) are provided.

| **ID** | **Subregion** | **Anatomy** | ***F*** | **Patial $\eta^2$** | **90% CI** | ***P*** | **$P_{adj}$** |
|---|---|---|---|---|---|---|---|
| 120 | TH_R | PhG | 8.055 | 0.061 | [0.019, 0.111] | < .001 | .013 |
| 135 | A39c_L | IPL | 10.583 | 0.078 | [0.030, 0.133] | < .001 | .005 |
| 151 | dmPOS_L | Pcun | 7.320 | 0.056 | [0.015, 0.104] | .001 | .022 |
| 163 | G_L | INS | 9.332 | 0.070 | [0.024, 0.122] | < .001 | .007 |
| 172 | dIg_R | INS | 10.109 | 0.075 | [0.028, 0.129] | < .001 | .005 |
| 182 | A23v_R | CG | 8.703 | 0.065 | [0.021, 0.117] | < .001 | .008 |
| 195 | rLinG_L | MVOcC | 9.089 | 0.068 | [0.023, 0.120] | < .001 | .007 |
| 197 | vmPOS_L | MVOcC | 13.064 | 0.095 | [0.042, 0.153] | < .001 | .001 |
| 241 | Otha_L | Tha | 8.979 | 0.067 | [0.023, 0.119] | < .001 | .007 |

**eTable 19.** Post−hoc comparisons of SC−FC coupling differences among healthy controls, adolescent MDDs with and without major life events (SB+ and SB−). Both FDR corrected p-value ($P_{adj}$) and original p-value ($P$) are provided.

| **Subregion** | **Constrast** | ***T*** | **Cohen's *d*** | **95% CI** | ***P*** | $P_{adj}$ |
|---|---|---|---|---|---|---|
| TH_R | SB− vs. HC | 3.720 | 0.471 | [0.219, 0.723] | .001 | .002 |
| | SB+ vs. HC | 3.509 | 0.445 | [0.193, 0.696] | .002 | .002 |
| | SB+ vs. SB− | 0.011 | 0.001 | [−0.247, 0.250] | < .001 | < .001 |
| A39c_L | SB− vs. HC | 4.322 | 0.548 | [0.294, 0.800] | < .001 | < .001 |
| | SB+ vs. HC | 3.941 | 0.500 | [0.247, 0.751] | < .001 | < .001 |
| | SB+ vs. SB− | −0.149 | −0.019 | [−0.267, 0.230] | .988 | .988 |
| dmPOS_L | SB− vs. HC | 3.359 | 0.426 | [0.174, 0.677] | .003 | .004 |
| | SB+ vs. HC | 3.535 | 0.448 | [0.196, 0.699] | .001 | .004 |
| | SB+ vs. SB− | 0.446 | 0.057 | [−0.192, 0.305] | .896 | .896 |
| G_L | SB− vs. HC | 3.942 | 0.500 | [0.247, 0.751] | < .001 | .001 |
| | SB+ vs. HC | 3.850 | 0.488 | [0.235, 0.740] | < .001 | .001 |
| | SB+ vs. SB− | 0.169 | 0.021 | [−0.227, 0.270] | .984 | .984 |
| dIg_R | SB− vs. HC | 4.496 | 0.570 | [0.316, 0.823] | < .001 | < .001 |
| | SB+ vs. HC | 2.830 | 0.359 | [0.108, 0.609] | .014 | .021 |
| | SB+ vs. SB− | −1.669 | −0.212 | [−0.460, 0.038] | .219 | .219 |
| A23v_R | SB− vs. HC | 3.702 | 0.469 | [0.217, 0.721] | .001 | .001 |
| | SB+ vs. HC | 3.822 | 0.484 | [0.232, 0.736] | < .001 | .001 |
| | SB+ vs. SB− | 0.404 | 0.051 | [−0.197, 0.300] | .914 | .914 |
| rLinG_L | SB− vs. HC | 4.243 | 0.538 | [0.284, 0.790] | < .001 | < .001 |
| | SB+ vs. HC | 2.990 | 0.379 | [0.128, 0.629] | .009 | .013 |
| | SB+ vs. SB− | −1.195 | −0.151 | [−0.400, 0.098] | .458 | .458 |
| vmPOS_L | SB− vs. HC | 4.246 | 0.538 | [0.285, 0.791] | < .001 | < .001 |
| | SB+ vs. HC | 4.881 | 0.619 | [0.364, 0.872] | < .001 | < .001 |
| | SB+ vs. SB− | 1.056 | 0.134 | [−0.115, 0.382] | .542 | .542 |
| Otha_L | SB− vs. HC | 4.217 | 0.535 | [0.281, 0.787] | < .001 | < .001 |
| | SB+ vs. HC | 2.970 | 0.376 | [0.125, 0.627] | .009 | .014 |
| | SB+ vs. SB− | −1.189 | −0.151 | [−0.399, 0.098] | .461 | .461 |

© 2024 Xu M et al. *JAMA Network Open*

**eTable 20.** Partial Spearman correlation coefficients between SC−FC coupling and HAMD-17.

| **ID** | **Subregion** | **Anatomy** | ***r*** | **95% CI** | ***P*** |
|---|---|---|---|---|---|
| 6 | A9l_R | SFG | −0.223 | [−0.364, −0.072] | .004 |
| 14 | A10m_R | SFG | −0.155 | [−0.300, −0.003] | .050 |
| 18 | IFJ_R | MFG | −0.211 | [−0.352, −0.060] | .007 |
| 40 | A44v_R | IFG | −0.186 | [−0.330, −0.035] | .018 |
| 62 | A4tl_R | PrG | 0.156 | [0.004, 0.301] | .048 |
| 86 | A37dl_R | MTG | −0.163 | [−0.308, −0.011] | .039 |
| 100 | A20cl_R | ITG | −0.211 | [−0.353, −0.060] | .007 |
| 130 | A5l_R | SPL | −0.183 | [−0.327, −0.032] | .020 |
| 132 | A7pc_R | SPL | −0.171 | [−0.315, −0.019] | .030 |
| 138 | A39rd_R | IPL | −0.163 | [−0.308, −0.011] | .039 |
| 188 | A32sg_R | CG | −0.162 | [−0.307, −0.010] | .040 |
| 191 | rCunG_L | MVOcC | 0.162 | [0.010, 0.307] | .040 |
| 192 | rCunG_R | MVOcC | 0.208 | [0.056, 0.350] | .008 |
| 194 | cCunG_R | MVOcC | 0.183 | [0.031, 0.326] | .020 |
| 198 | vmPOS_R | MVOcC | 0.198 | [0.046, 0.340] | .012 |
| 205 | iOccG_L | LOcC | 0.156 | [0.004, 0.301] | .048 |
| 210 | lsOccG_R | LOcC | 0.183 | [0.032, 0.327] | .020 |
| 212 | mAmyg_R | Amyg | 0.156 | [0.004, 0.302] | .048 |

© 2024 Xu M et al. *JAMA Network Open*

**eTable 21.** Partial Spearman correlation coefficients between SC−FC coupling and HAMA.

| **ID** | **Subregion** | **Anatomy** | ***r*** | **95% CI** | ***P*** |
|---|---|---|---|---|---|
| 6 | A9l_R | SFG | −0.215 | [−0.357, −0.064] | .006 |
| 14 | A10m_R | SFG | −0.181 | [−0.324, −0.029] | .022 |
| 16 | A9/46d_R | MFG | −0.158 | [−0.304, −0.006] | .045 |
| 18 | IFJ_R | MFG | −0.247 | [−0.386, −0.097] | .002 |
| 40 | A44v_R | IFG | −0.271 | [−0.408, −0.122] | < .001 |
| 42 | A14m_R | OrG | −0.188 | [−0.331, −0.036] | .017 |
| 62 | A4tl_R | PrG | 0.208 | [0.056, 0.350] | .008 |
| 72 | A41/42_R | STG | 0.156 | [0.004, 0.302] | .047 |
| 99 | A20cl_L | ITG | −0.186 | [−0.329, −0.034] | .018 |
| 120 | TH_R | PhG | −0.181 | [−0.325, −0.029] | .022 |
| 126 | A7r_R | SPL | −0.170 | [−0.314, −0.018] | .031 |
| 137 | A39rd_L | IPL | −0.195 | [−0.338, −0.043] | .013 |
| 138 | A39rd_R | IPL | −0.173 | [−0.317, −0.021] | .029 |
| 144 | A39rv_R | IPL | −0.173 | [−0.317, −0.021] | .028 |
| 153 | A31_L | PCun | −0.173 | [−0.317, −0.021] | .028 |
| 166 | vIa_R | INS | −0.160 | [−0.305, −0.008] | .042 |
| 167 | dIa_L | INS | −0.157 | [−0.302, −0.005] | .046 |
| 170 | vId/vIg_R | INS | −0.188 | [−0.331, −0.037] | .017 |
| 187 | A32sg_L | CG | −0.177 | [−0.321, −0.025] | .025 |
| 188 | A32sg_R | CG | −0.203 | [−0.345, −0.051] | .010 |

© 2024 Xu M et al. *JAMA Network Open*

## REFERENCES


1. Goñi J, van den Heuvel MP, Avena-Koenigsberger A, et al. Resting-brain functional connectivity predicted by analytic measures of network communication. *Proceedings of the National Academy of Sciences*. 2014/01/14 2014;111(2):833-838. doi:10.1073/pnas.1315529111
2. Bernstein DP, Ahluvalia T, Pogge D, Handelsman L. Validity of the Childhood Trauma Questionnaire in an adolescent psychiatric population. *J Am Acad Child Adolesc Psychiatry*. Mar 1997;36(3):340-8. doi:10.1097/00004583-199703000-00012
3. Huang D, Liu Z, Cao H, Yang J, Wu Z, Long Y. Childhood trauma is linked to decreased temporal stability of functional brain networks in young adults. *J Affect Disord*. Jul 1 2021;290:23-30. doi:10.1016/j.jad.2021.04.061
4. Van Dam NT, Rando K, Potenza MN, Tuit K, Sinha R. Childhood maltreatment, altered limbic neurobiology, and substance use relapse severity via trauma-specific reductions in limbic gray matter volume. *JAMA Psychiatry*. Aug 2014;71(8):917-25. doi:10.1001/jamapsychiatry.2014.680
5. Posner K, Brown GK, Stanley B, et al. The Columbia-Suicide Severity Rating Scale: initial validity and internal consistency findings from three multisite studies with adolescents and adults. *Am J Psychiatry*. Dec 2011;168(12):1266-77. doi:10.1176/appi.ajp.2011.10111704
6. Ulmer Yaniv A, Salomon R, Waidergoren S, Shimon-Raz O, Djalovski A, Feldman R. Synchronous caregiving from birth to adulthood tunes humans' social brain. *Proceedings of the National Academy of Sciences*. 2021/04/06 2021;118(14):e2012900118. doi:10.1073/pnas.2012900118
7. Esteban O, Markiewicz CJ, Blair RW, et al. fMRIPrep: a robust preprocessing pipeline for functional MRI. *Nat Methods*. 2019/01/01 2019;16(1):111-116. doi:10.1038/s41592-018-0235-4
8. Cieslak M, Cook PA, He X, et al. QSIPrep: an integrative platform for preprocessing and reconstructing diffusion MRI data. *Nat Methods*. 2021/07/01 2021;18(7):775-778. doi:10.1038/s41592-021-01185-5
9. Gorgolewski K, Burns C, Madison C, et al. Nipype: A Flexible, Lightweight and Extensible Neuroimaging Data Processing Framework in Python. Original Research. 2011-August-22 2011;5(13)doi:10.3389/fninf.2011.00013
10. Abraham A, Pedregosa F, Eickenberg M, et al. Machine learning for neuroimaging with scikit-learn. Methods. 2014-February-21 2014;8doi:10.3389/fninf.2014.00014
11. Garyfallidis E, Brett M, Amirbekian B, et al. Dipy, a library for the analysis of diffusion MRI data. *Front Neuroinform*. 2014;8:8. doi:10.3389/fninf.2014.00008
12. Tournier JD, Smith R, Raffelt D, et al. MRtrix3: A fast, flexible and open software framework for medical image processing and visualisation. *NeuroImage*. 2019/11/15/ 2019;202:116137. doi:https://doi.org/10.1016/j.neuroimage.2019.116137
13. Tustison NJ, Avants BB, Cook PA, et al. N4ITK: Improved N3 Bias Correction. *IEEE Transactions on Medical Imaging*. 2010;29(6):1310-1320. doi:10.1109/TMI.2010.2046908
14. Zhang Y, Brady M, Smith S. Segmentation of brain MR images through a hidden Markov random field model and the expectation-maximization algorithm. *IEEE Transactions on Medical Imaging*. 2001;20(1):45-57. doi:10.1109/42.906424
15. Fonov VS, Evans AC, McKinstry RC, Almli CR, Collins DL. Unbiased nonlinear average age-appropriate brain templates from birth to adulthood. *NeuroImage*. 2009/07/01/ 2009;47:S102. doi:https://doi.org/10.1016/S1053-8119(09)70884-5
16. Avants BB, Epstein CL, Grossman M, Gee JC. Symmetric diffeomorphic image registration with cross-correlation: Evaluating automated labeling of elderly and neurodegenerative brain. *Medical Image Analysis*. 2008/02/01/


© 2024 Xu M et al. *JAMA Network Open*

2008;12(1):26-41. doi:https://doi.org/10.1016/j.media.2007.06.004
17. Jenkinson M, Bannister P, Brady M, Smith S. Improved Optimization for the Robust and Accurate Linear Registration and Motion Correction of Brain Images. *NeuroImage*. 2002/10/01/ 2002;17(2):825-841. doi:https://doi.org/10.1006/nimg.2002.1132
18. Cox RW, Hyde JS. Software tools for analysis and visualization of fMRI data. 1997;10(4-5):171-178. doi:https://doi.org/10.1002/(SICI)1099-1492(199706/08)10:4/5<171::AID-NBM453>3.0.CO;2-L
19. Wang S, Peterson DJ, Gatenby JC, Li W, Grabowski TJ, Madhyastha TM. Evaluation of Field Map and Nonlinear Registration Methods for Correction of Susceptibility Artifacts in Diffusion MRI. Original Research. 2017-February-21 2017;11doi:10.3389/fninf.2017.00017
20. Treiber JM, White NS, Steed TC, et al. Characterization and Correction of Geometric Distortions in 814 Diffusion Weighted Images. *PLOS ONE*. 2016;11(3):e0152472. doi:10.1371/journal.pone.0152472
21. Jenkinson M, Smith S. A global optimisation method for robust affine registration of brain images. *Medical Image Analysis*. 2001/06/01/ 2001;5(2):143-156. doi:https://doi.org/10.1016/S1361-8415(01)00036-6
22. Greve DN, Fischl B. Accurate and robust brain image alignment using boundary-based registration. *NeuroImage*. 2009/10/15/ 2009;48(1):63-72. doi:https://doi.org/10.1016/j.neuroimage.2009.06.060
23. Lanczos C. Evaluation of Noisy Data. *Journal of the Society for Industrial and Applied Mathematics Series B Numerical Analysis*. 1964/01/01 1964;1(1):76-85. doi:10.1137/0701007
24. Power JD, Mitra A, Laumann TO, Snyder AZ, Schlaggar BL, Petersen SE. Methods to detect, characterize, and remove motion artifact in resting state fMRI. *NeuroImage*. 2014/01/01/ 2014;84:320-341. doi:https://doi.org/10.1016/j.neuroimage.2013.08.048
25. Veraart J, Novikov DS, Christiaens D, Ades-Aron B, Sijbers J, Fieremans E. Denoising of diffusion MRI using random matrix theory. *Neuroimage*. Nov 15 2016;142:394-406. doi:10.1016/j.neuroimage.2016.08.016
26. Andersson JLR, Graham MS, Zsoldos E, Sotiropoulos SN. Incorporating outlier detection and replacement into a non-parametric framework for movement and distortion correction of diffusion MR images. *Neuroimage*. Nov 1 2016;141:556-572. doi:10.1016/j.neuroimage.2016.06.058
27. Behrens TE, Berg HJ, Jbabdi S, Rushworth MF, Woolrich MW. Probabilistic diffusion tractography with multiple fibre orientations: What can we gain? *Neuroimage*. Jan 1 2007;34(1):144-55. doi:10.1016/j.neuroimage.2006.09.018
28. Zamani Esfahlani F, Faskowitz J, Slack J, Mišić B, Betzel RF. Local structure-function relationships in human brain networks across the lifespan. *Nature Communications*. 2022/04/19 2022;13(1):2053. doi:10.1038/s41467-022-29770-y
29. Estrada E, Hatano N. Communicability in complex networks. *Physical review E, Statistical, nonlinear, and soft matter physics*. Mar 2008;77(3 Pt 2):036111. doi:10.1103/PhysRevE.77.036111
30. Hilgetag CC, Burns GA, O'Neill MA, Scannell JW, Young MP. Anatomical connectivity defines the organization of clusters of cortical areas in the macaque monkey and the cat. *Philosophical transactions of the Royal Society of London Series B, Biological sciences*. Jan 29 2000;355(1393):91-110. doi:10.1098/rstb.2000.0551
31. Seguin C, van den Heuvel MP, Zalesky A. Navigation of brain networks. 2018;115(24):6297-6302. doi:doi:10.1073/pnas.1801351115
32. Goñi J, Avena-Koenigsberger A, Velez de Mendizabal N, van den Heuvel MP, Betzel RF, Sporns O. Exploring the Morphospace of Communication Efficiency in Complex Networks. *PLOS ONE*. 2013;8(3):e58070. doi:10.1371/journal.pone.0058070
33. Betzel RF, Griffa A, Avena-Koenigsberger A, et al. Multi-scale community organization of the human structural connectome and its relationship with resting-state functional connectivity. *Network Science*. 2013;1(3):353-373. doi:10.1017/nws.2013.19

© 2024 Xu M et al. *JAMA Network Open*

34. Lambiotte R, Sinatra R, Delvenne JC, Evans TS, Barahona M, Latora V. Flow graphs: Interweaving dynamics and structure. *Physical Review E*. 07/25/ 2011;84(1):017102. doi:10.1103/PhysRevE.84.017102
35. Ho D, Imai K, King G, Stuart EA. MatchIt: Nonparametric Preprocessing for Parametric Causal Inference. *Journal of Statistical Software*. 06/14 2011;42(8):1 - 28. doi:10.18637/jss.v042.i08
36. Váša F, Seidlitz J, Romero-Garcia R, et al. Adolescent Tuning of Association Cortex in Human Structural Brain Networks. *Cereb Cortex*. Jan 1 2018;28(1):281-294. doi:10.1093/cercor/bhx249
37. Alexander-Bloch AF, Shou H, Liu S, et al. On testing for spatial correspondence between maps of human brain structure and function. *Neuroimage*. Sep 2018;178:540-551. doi:10.1016/j.neuroimage.2018.05.070
38. Baller EB, Valcarcel AM, Adebimpe A, et al. Developmental coupling of cerebral blood flow and fMRI fluctuations in youth. *Cell Rep*. Mar 29 2022;38(13):110576. doi:10.1016/j.celrep.2022.110576

© 2024 Xu M et al. *JAMA Network Open*
