# Supplementary material for: Reconfiguration of Structural and Functional Connectivity Coupling in Patient Subgroups With Adolescent Depression
Source: JAMA Netw Open. 2024 Mar 12;7(3):e241933. doi: 10.1001/jamanetworkopen.2024.1933 (PMC10933730; doi:10.1001/jamanetworkopen.2024.1933)
Supplement: Supplement 2. — Data Sharing Statement [file jamanetwopen-e241933-s002.pdf]

Data Sharing Statement

Xu. Reconfiguration of Structural and Functional Connectivity Coupling in Patient Subgroups With Adolescent Depression. *JAMA Netw Open*. Published March 12, 2024. doi:10.1001/jamanetworkopen.2024.1933

Data
Data available: Yes
Data types: Data dictionary
How to access data: Data will be made available on request. Please contact jsui@bnu.edu.cn.
When available: With publication

Supporting Documents
Document types: Statistical/analytic code
How to access documents: Statistical/analytic code will be made available on request. Please contact the corresponding author
When available: With publication

Additional Information
Who can access the data: Researchers whose proposed use of the data has been approved.
Types of analyses: For research/non-commercial purpose.
Mechanisms of data availability: After approval of a proposal.
